# Effect of Ligand Substituents on Spectroscopic and Catalytic Properties of Water-Compatible Cp*Ir-(pyridinylmethyl)sulfonamide-Based Transfer Hydrogenation Catalysts

**DOI:** 10.1021/acs.inorgchem.3c04040

**Published:** 2024-02-12

**Authors:** Rosalind L. Booth, Adrian C. Whitwood, Anne-K. Duhme-Klair

**Affiliations:** Department of Chemistry, University of York, York YO10 5DD, U.K.

## Abstract

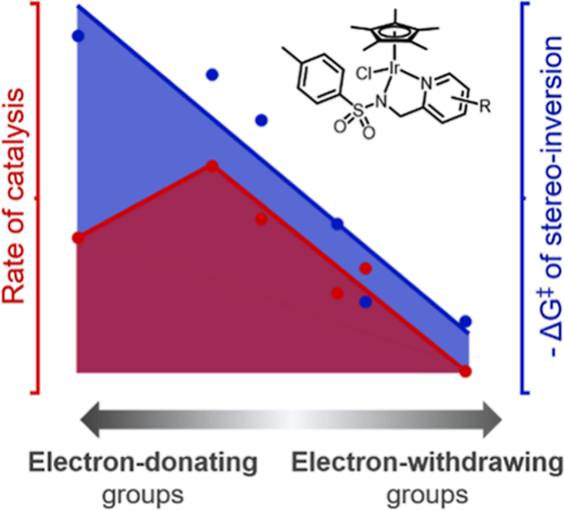

Transition-metal-based hydrogenation catalysts have applications ranging from high-value chemical synthesis to medicinal chemistry. A series of (pyridinylmethyl)sulfonamide ligands substituted with electron-withdrawing and -donating groups were synthesized to study the influence of the electronic contribution of the bidentate ligand in Cp*Ir piano-stool complexes. A variable-temperature NMR investigation revealed a strong correlation between the electron-donating ability of the substituent and the rate of stereoinversion of the complexes. This correlation was partially reflected in the catalytic activity of the corresponding catalysts. Complexes with electron-withdrawing substituents followed the trend observed in the variable-temperature NMR study, thereby confirming the rate-determining step to be donation of the hydride ligand. Strongly electron-donating groups, on the other hand, caused a change in the rate-determining step in the formation of the iridium-hydride species. These results demonstrate that the activity of these catalysts can be tuned systematically via changes in the electronic contribution of the bidentate (pyridinylmethyl)sulfonamide ligands.

## Abstract 2

A series of (pyridinylmethyl)sulfonamide ligands with substituents on the pyridine ring were synthesized to study the influence of the electronic contribution of the bidentate ligand in Cp*Ir piano-stool complexes. Correlations between the electron-donating ability of the substituent, the rate of stereoinversion of the complexes, and the rate of catalysis provided insight into the catalytic mechanism and demonstrated that these catalysts can be tuned systematically via changes in the electronic contribution of the bidentate ligand.

## Introduction

**Figure 1:**
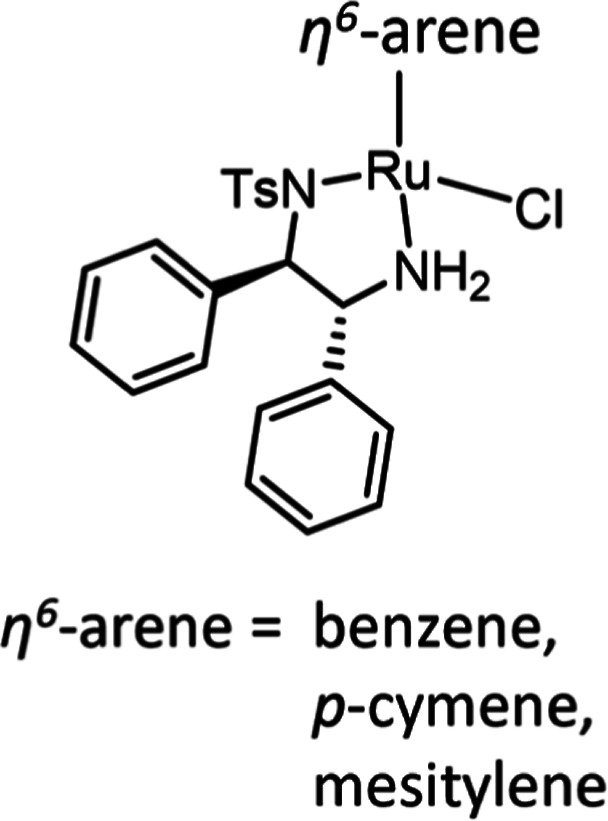
Structure of the Ru(II)-TsDPEN precatalyst reported by Noyori and coworkers.^4^

The synthesis of chiral amines is key in the manufacture of pharmaceuticals, as the basis of several valuable chiral intermediates and building blocks.^1^ One method of accessing these compounds is through asymmetric transfer hydrogenation of pro-chiral imine precursors using transition-metal catalysts.^2,3^ The discovery of Ru(II)-TsDPEN [TsDPEN = *N*-(*p*-toluenesulfonyl)-1,2-diphenyl-ethylenediamine] catalysts (Figure 1) for the reduction of aromatic ketones by Noyori and Ikariya,^4^ later expanded to imines,^5^ was an important breakthrough with reported enantioselectivities of up to 99%.^5,6^ The chiral TsDPEN ligand is proposed to influence enantioselectivity by controlling the configuration at the metal center of the complex which, together with a favorable C–H···π interaction to orientate the substrate, determines which face of the sp^2^ carbon the hydride is added to.^7^ The addition of the TsDPEN ligand has been extended to Ir and Rh piano-stool complexes with similarly high enantioselectivity.^8^

**Figure 2:**
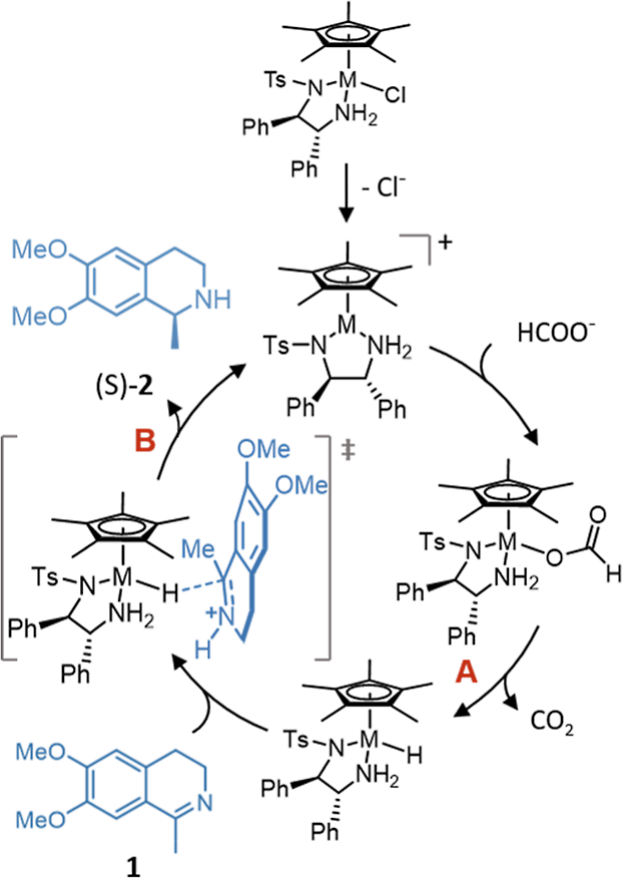
Mechanism for the transfer hydrogenation of protonated imines by Noyori–Ikariya-type^4^ catalysts (M = Rh and Ir). Step A in the cycle is the formation of the metal-hydride species by β-hydride elimination and step B is hydride donation.

The catalytic mechanism for the transfer hydrogenation of ketones and imines has been the subject of a number of studies. The mechanistic model shown in Figure 2 was proposed following extensive analysis of experimental results of imine reduction catalyzed by [(η^6^-arene)Ru(TsDPEN)Cl]- and [Cp*Rh(TsDPEN)Cl]-derived catalysts and was supported by DFT calculations.^9−11^ It differs from the proposed ketone reduction mechanism which reasoned that the reaction proceeds via a six-membered transition state,^10,12−16^ where a proton is transferred from a protonated amine group of the ligand and the hydride from the metal center in a concerted step.^12,17,18^ Since the imine nitrogen is more easily protonated than the oxygen of its ketone counterpart, it has been recognized that in aqueous media, the transfer of the proton and hydride is more likely to occur in a sequential, rather than concerted, manner.^16,19−23^ Two key steps in the catalytic cycle are the formation of the metal-hydride species by β-hydride elimination (step A; Figure 2), followed by hydride donation to the substrate (step B; Figure 2). Studies of related Ir piano-stool catalysts have noted close competition in the relative rates of the formation of the metal-hydride species and donation of the hydride ligand, with factors such as pH found to control which step was rate-determining.^24^

Initially, these catalysts were mainly utilized for the transfer hydrogenation of ketones, aldehydes, and imines in organic solvents;^25,26^ however, their stability in air and water^19,27^ has sparked interest in applying these catalysts in more sustainable processes.^28^ Additionally, related complexes have been employed for a number of other applications including medicinal chemistry^29−31^ and artificial metalloenzymes.^32^

The Duhme-Klair group have reported a related Cp*Ir catalyst incorporated within an artificial metalloenzyme, with a (pyridinylmethyl)sulfonamide ligand replacing the chiral TsDPEN ligand.^33^ This nonchiral ligand is not stereodirecting; instead, enantioselectivity in catalysis is derived from the secondary coordination sphere supplied by the protein scaffold. One step in optimizing the catalytic performance of artificial metalloenzymes is through enhancing the electronic properties of the ligands that surround the catalytic metal center.^34−36^

**Figure 3:**
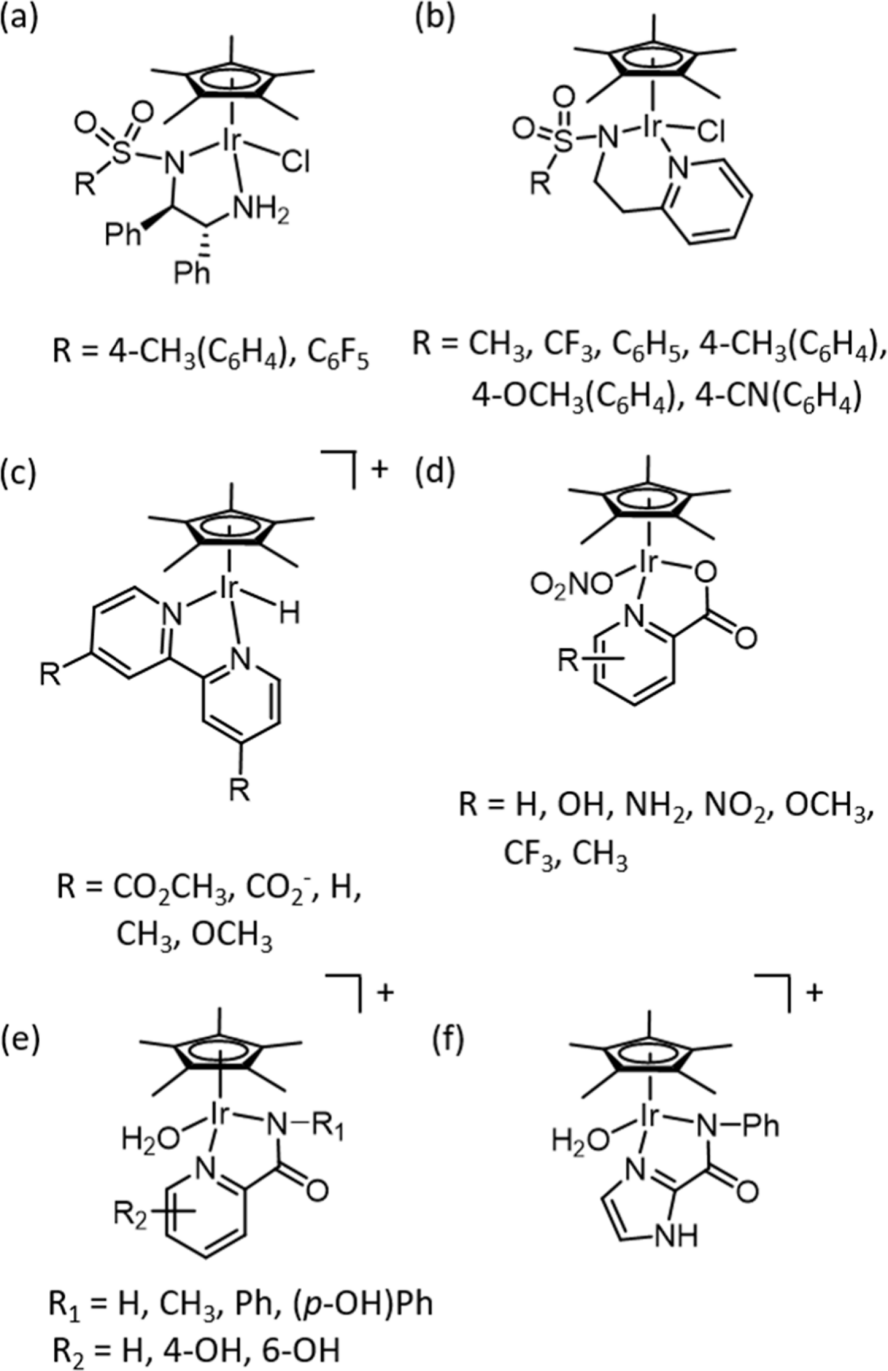
Examples of related piano-stool iridium complexes used to investigate the effect of the electronic contribution of the bidentate ligand in transfer hydrogenation catalysts (a,b), study of the “hydricity” of the metal complex (c), and catalysts for water oxidation (d) and CO_2_ hydrogenation (e,f).

Several studies have investigated whether the catalytic performance of Cp*Ir catalysts can be enhanced by altering the electronic properties of the bidentate ligand. The majority of these works have focused on the addition of electron-withdrawing or -donating groups neighboring the sulfonamide nitrogen,^19,27^ mostly targeting transfer hydrogenation of ketones. In one report,^19^ the effect of adding electron-withdrawing substituents at the sulfonyl group of (aminoethyl)sulfonamide ligands (Figure 3a) revealed that the addition of strongly electron-withdrawing groups improved the selectivity and activity of these catalysts. This was in contradiction to a previous report with Ru(II)-based catalysts of the same ligand type and was also opposed by a study of [Cp*Ir(pyridinylethylsulfonamide)Cl]-derived catalysts (Figure 3b).^4,27^ These studies reported that electron-donating substituents at the sulfonyl group were advantageous in improving catalyst performance, and there was a strong correlation between catalytic conversions and the Hammett parameters, a measure of the electron-withdrawing and -donating ability of the substituents. These contrasting reports perhaps highlight the significant influence that reaction conditions have on catalysis.

A seminal approach to rationalizing catalyst performance with electron-withdrawing or -donating substituents on the pyridine-containing ligands utilized [Cp*Ir(bipyridine)H] complexes to demonstrate how substituent groups on pyridine influenced the “hydricity” of these catalysts (Figure 3c).^37^ “Hydricity” is formally the free energy required to break a M–H bond and, hence, acts as a measure of the hydride donor ability of a metal complex.^38^ The measurement of the free energy of hydride donation varies with both pH and composition of the solution, but it was found that hydricity values correlate strongly with the Hammett parameters of substituents on the bipyridine rings, with electron-donating groups lowering the free energy required to break the Ir–hydride bond and vice versa for electron-withdrawing groups.

The aforementioned study, together with several others of pyridine-containing transition-metal catalysts,^39−44^ is focused largely on substituents at the position *para* to the *metal*-coordinating nitrogen. In these examples, the resonance effects of the substituent outweigh the inductive effects. While in some cases reactivity trends of catalysts correlate well with specific Hammett parameters (e.g., σ_p_),^40,43,45^ other examples are poorly described by these parameters,^41,46^ particularly with regards to the nonadditivity of substituent effects.^47^ Some deviation in trends from traditional Hammett parameters is perhaps not surprising since they are derived from the effect of a substituent on the ionization of benzoic acid, a system significantly different from the influence of a substituent on a metal-coordinating pyridine ligand.

Far fewer studies have examined the effect of substituents at the *ortho-* or *meta-* positions on the pyridine ring. One study which did examine the effect of substituting the pyridine ligand at different positions compared catalysts for iridium-catalyzed water oxidation and showed that substituent position did influence catalytic activity and correlated somewhat with relevant Hammett parameters but not for all substituents (Figure 3d).^48^ Likewise, complexes with closely related picolinamidate-type ligands (Figure 3e,f) have been investigated with a range of substituents at both the pyridine ring and the amide nitrogen. These studies corroborate the finding that increasing electron-donating ability of the ligand benefits catalytic activity.^49,50^

To date, investigations examining the effect of the electronic contributions of the ligands on transfer hydrogenation catalysts have disproportionately targeted the reduction of ketones over imines. Furthermore, most of these investigations have focused on modifications at the sulfonamide nitrogen site. Our aim was to investigate the effect of electron-withdrawing and -donating substituents at the pyridine ring of the ligand, the alternative metal-coordinating nitrogen site.

**Figure 4:**
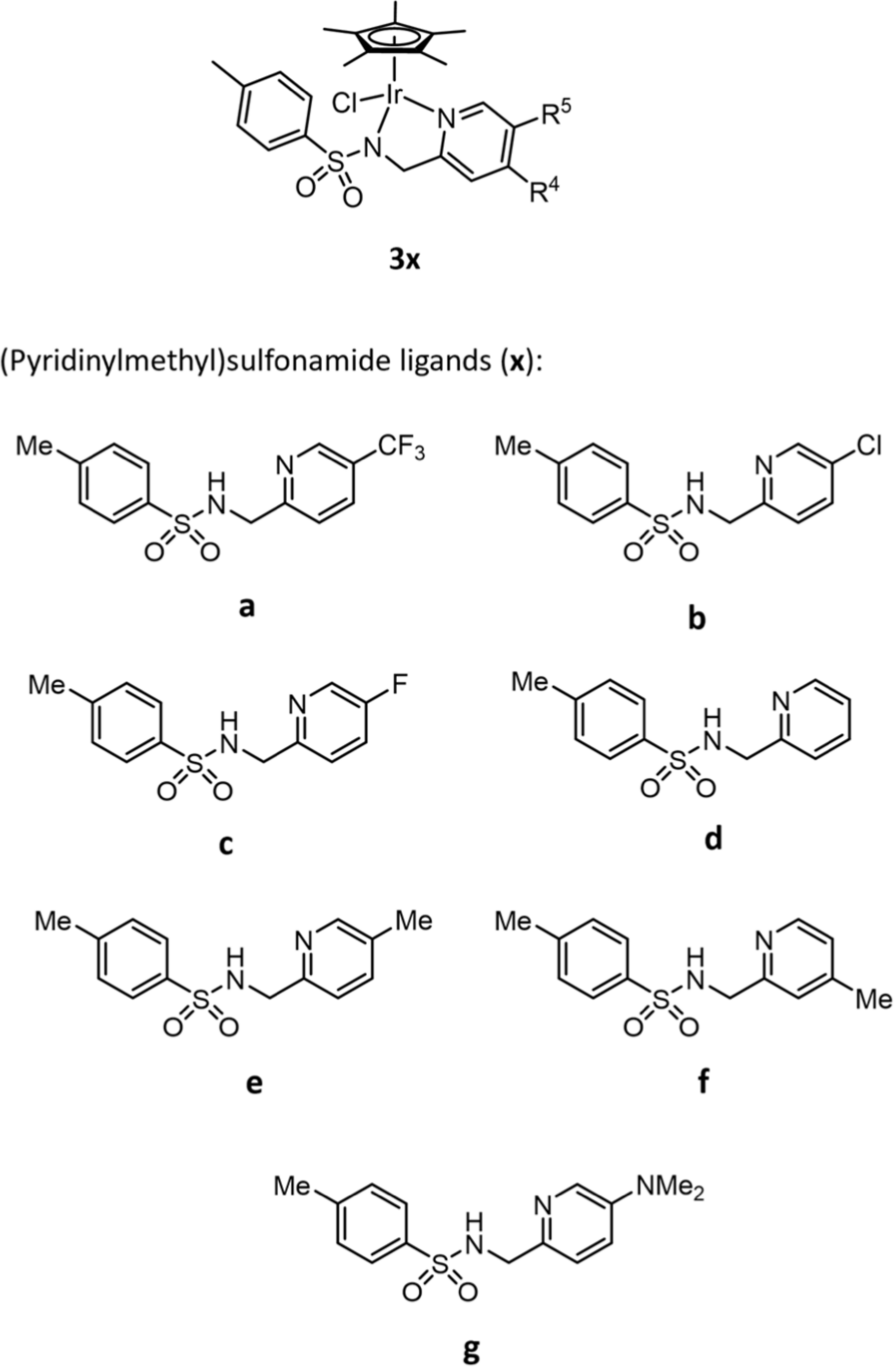
Structure of the complexes (**3x**) investigated in this study, composed of ligands **a**–**g**.

Analyzing the crystal structure of our artificial metalloenzyme indicated that substituents in the *meta-*position of the pyridine ring would be best accommodated by our protein scaffold. Consequently, we selected a range of ligands with electron-withdrawing and -donating substituents at the *meta-*position (Figure 4), with an additional ligand **f** with a methyl substituent at the *para-*position to compare if the position of the substituent would additionally affect the catalyst.

## Results and Discussion

### Synthesis and Characterization

The majority of (pyridinylmethyl)sulfonamide ligands could be prepared from commercially available pyridine methyl amine precursors by reacting with tosyl chloride and a base, such as triethylamine or *N*,*N*-diisopropylethylamine.^33^ The dimethylamine-substituted pyridine precursor was not commercially available and was prepared by a method adapted from the literature.^51^

The corresponding iridium complexes were prepared by dissolving two equivalents of the corresponding ligand and one equivalent of [Cp*IrCl_2_]_2_ in dichloromethane before the slow addition of two equivalents of NaOH. Following sonication for 20 min, the solutions were washed with water before the complexes were isolated from the organic layer as orange crystals.

**Figure 5:**
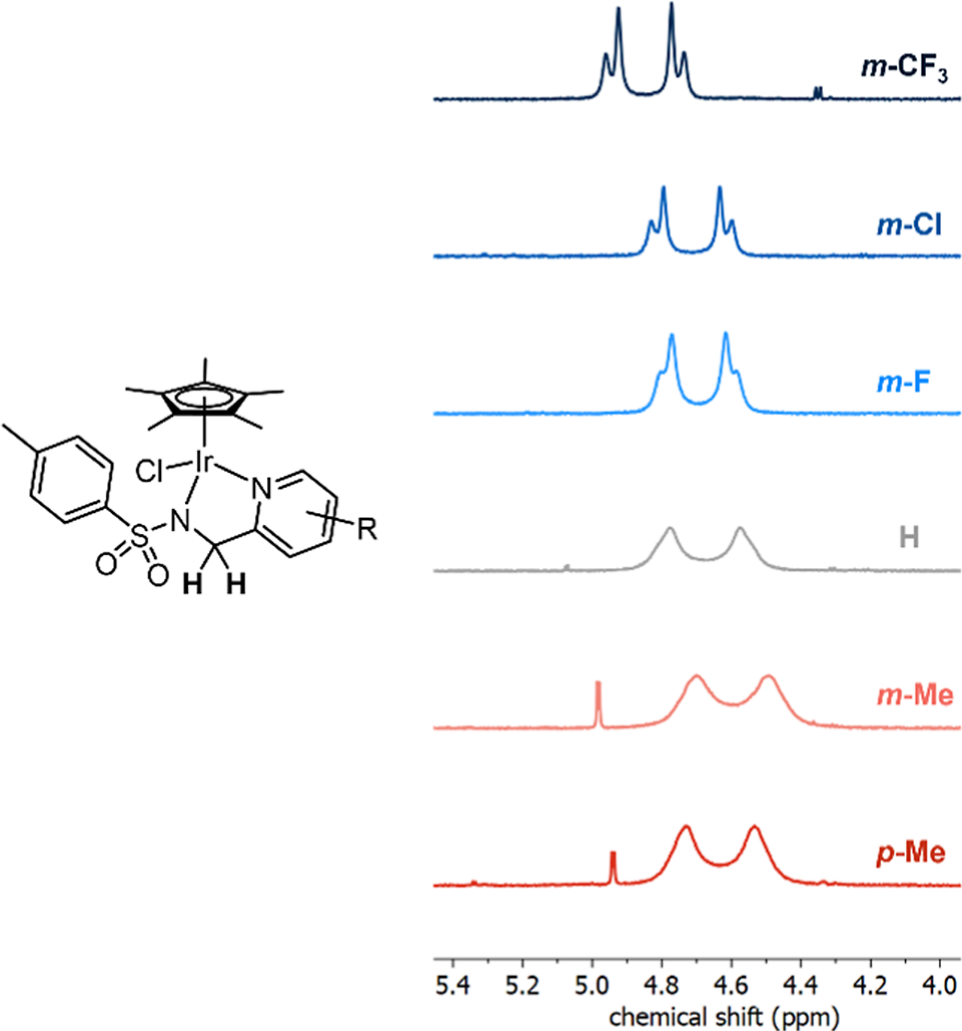
^1^H NMR spectra at 323 K in dimethylformamide-*d*_7_ (DMF-*d*_7_) of complexes **3a**–**f** are shown in the 4.0–5.2 ppm region to illustrate how the peak shape of the resonances assigned to the two diastereotopic CH_2_ protons varies with the substituent on the pyridine ring.

Due to the chirality of the complexes, two resonances in the ^1^H NMR spectra could be assigned to the diastereotopic protons of the CH_2_ position of the (pyridinylmethyl)sulfonamide ligands (Figure 5). For the complexes bearing an electron-withdrawing substituent on the pyridine ring, these two resonances are well resolved into doublets caused by ^2^*J* coupling between the two diastereotopic protons, with ^2^*J* coupling constants of 16.5–18.0 Hz, measured in chloroform-*d* at room temperature. In contrast, the complexes of the unsubstituted pyridine ring or those bearing electron-donating groups show two broad resonances in this region. Broadening of these resonances is due to the inversion of the stereocenter of the complex on the NMR time scale, as each diastereotopic proton exchanges between the two positions.

### Spectroscopic Studies

The observation that the resonances associated with the CH_2_ protons in the room-temperature ^1^H NMR spectra broadened with the addition of electron-donating groups relative to electron-withdrawing groups prompted us to further investigate the stereoinversion of these complexes. The rate at which the chiral center at iridium inverts can indirectly provide information about relative bonding interactions at the iridium metal center. This can assist in rationalizing and predicting relative catalytic activities of these catalysts since it is expected that the chemical changes occurring during stereoinversion, namely, the exchange of the labile monodentate ligand, resemble those in the activation of the precatalyst as well as the final step of the catalytic cycle, the hydride donation. Hence, where hydride donation is the rate-determining step in catalysis, variable-temperature ^1^H NMR spectroscopic measurements could be informative in catalyst design.

Thermodynamic parameters can be obtained from the relative rates of stereoinversion calculated by analysis of the line shape of the resonances between 4.2 and 4.9 ppm in the ^1^H NMR spectra obtained at multiple temperatures using the Bloch^52,53^ and the Eyring and Gibbs free energy equations (these are described in the Supporting Information).

**Figure 6:**
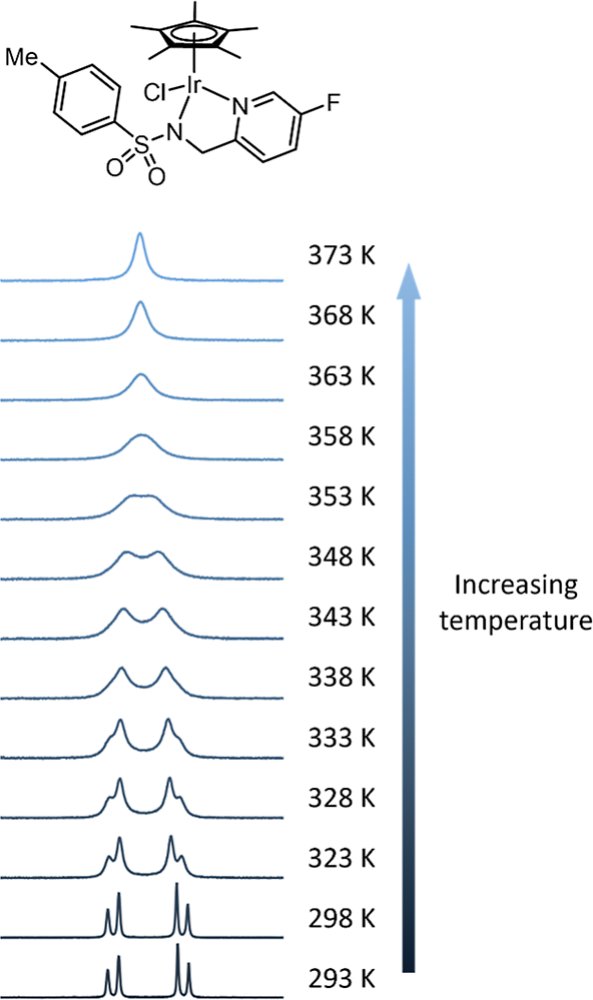
^1^H NMR spectra in DMF-*d*_7_ for complex **3c** acquired in the temperature range of 293–373 K.

^1^H NMR spectra were recorded in DMF-*d*_7_ (Figure 6) as this solvent provided the right temperature range for measurements. It should be noted that DMF is a coordinating solvent that is potentially able to exchange with the labile chlorido ligand, hence these measurements cannot provide the iridium–chloride bond strengths but can nevertheless reveal important information about general trends in iridium–ligand interactions.

As expected from the spectra at room temperature, the rate constants for stereoinversion increase with the electron-donating ability of the pyridine substituent. At temperatures where *k* was measurable for all complexes, the rate constant, *k*, increases sixfold between the complex with the strongest electron-withdrawing group, CF_3_, compared to that of the strongest electron-donating group, NMe_2_.

**Figure 7:**
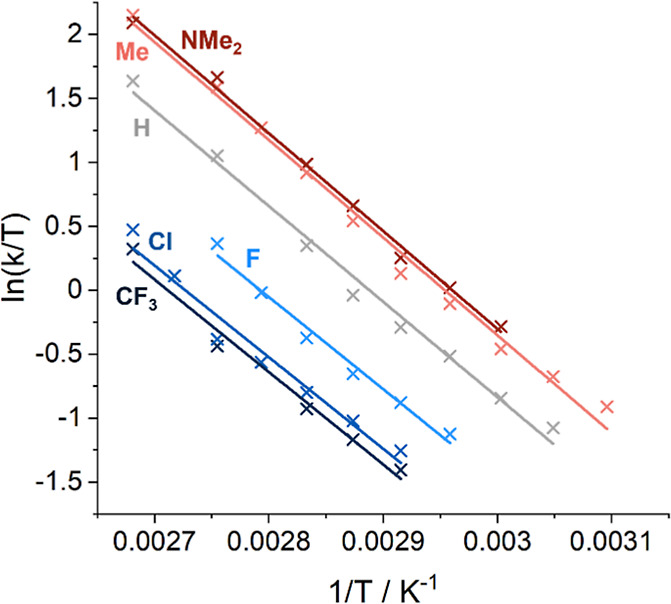
Plot of ln(*k*/*T*) vs 1/*T* to determine Δ*H*^⧧^ and Δ*S*^⧧^ labeled with the substituent, *R*, at the *meta-*position.

Figure 7 shows the natural log of rate/temperature plotted against the inverse temperature for complexes **3a**–**3g**. The gradient of the line is inversely proportional to the enthalpy of activation, as derived from the Eyring equation, while the entropy of activation can be calculated from the intercept. The similarity of the gradients obtained for complexes **3a**–**3g** suggests that Δ*H*^⧧^ values for all complexes are very similar. The difference in the observed rate of stereoinversion is therefore due to a larger variation in Δ*S*^⧧^ across the series (Table 1), which becomes increasingly more negative for complexes with electron-withdrawing substituents on the (pyridinylmethyl)sulfonamide ligand. A negative entropy of activation is indicative of an associative mechanism in the formation of the transition state, as previously observed in the solvolysis of Os(II)-based half-sandwich complexes.^54,55^ This assertion is further reinforced by the fact that the values of Δ*H*^⧧^ are significantly smaller than reported bond dissociation enthalpies for iridium–ligand bonds, for example, the iridium–hydride bond in Cp*Ir piano-stool complexes,^56^ and consistent with the presence of a large excess of coordinating DMF solvent molecules. A more negative Δ*S*^⧧^ results in a higher Δ*G*^⧧^ barrier and hence a slower rate of stereoinversion for complexes with electron-withdrawing substituents.

**Table 1:** Thermodynamic Parameters Derived from Variable-Temperature NMR Experiments

substituent	**σ**_*x*_	Δ*H*^⧧^ (kJ mol^–^^1^)	Δ*S*^⧧^ (J mol^–^^1^ K^–^^1^)	Δ*G*^⧧^ (at *T* = 298 K) (kJ mol^–^^1^)	Δ*G*^⧧^ (at *T* = 313 K) (kJ mol^–^^1^)
*m***-CF**_**3**_	0.43	60.1	–34.6	70.4	70.9
*m***-Cl**	0.37	59.5	–35.2	70.0	70.6
*m***-F**	0.34	59.9	–30.3	68.9	69.4
**H**	0	62.1	–18.1	67.5	67.8
**4-Me**	–0.07	63.5	–10.0	66.5	66.6
**5-Me**	–0.17	60.6	–20.6	66.8	67.1
**NMe**_**2**_	–0.16	63.6	–9.3	66.4	66.5

**Figure 8:**
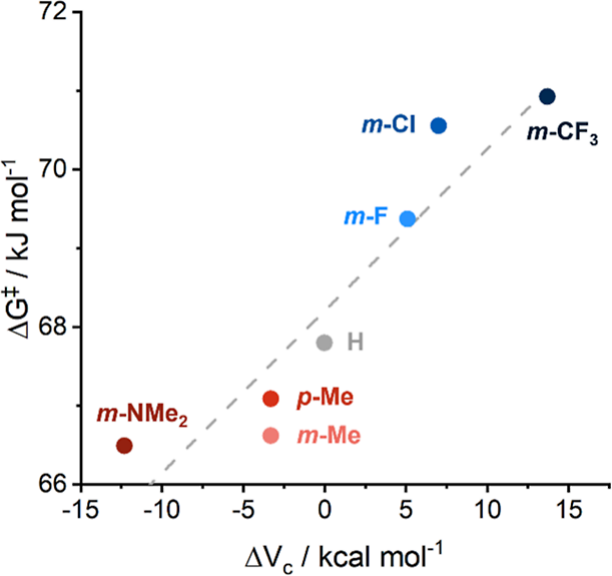
Plot of Δ*G*^⧧^ (at 313 K) for each of the iridium complexes **3a**–**3g** against the parameter Δ*V*_c_, a theoretical measure of the electron-donating or electron-withdrawing ability of the, respective, substituent shown.

The calculated Δ*G*^⧧^ values show a good correlation with Hammett parameters (see the Supporting Information) and with Δ*V*_c_ (Figure 8), a theoretical alternative to experimentally derived Hammett parameters. The Δ*V*_c_ parameter is formally the difference in the calculated molecular electrostatic potential at the nucleus of the *para* carbon of substituted benzene and a carbon atom in benzene. The calculated parameters correlate closely with experimentally derived Hammett parameters and have been demonstrated to adequately predict the cumulative effect of multiple substituents. These parameters also apply well to many other organic π-conjugated systems besides six-membered aromatic rings.^57^

### Catalytic Studies

**Figure 9:**
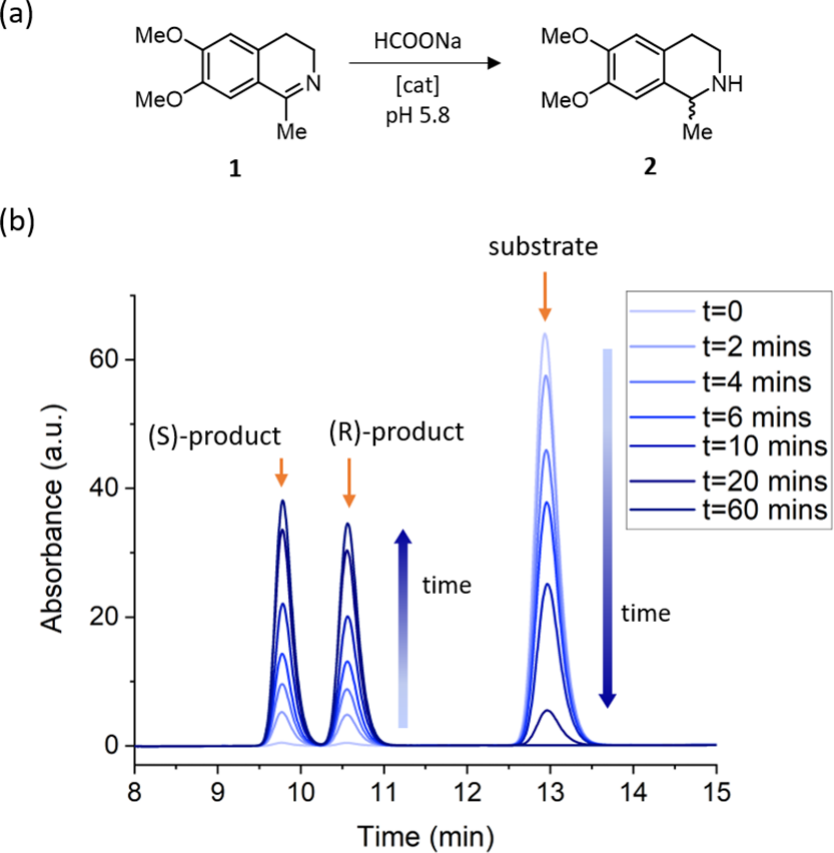
(a) Catalytic transformation investigated in this study and (b) example HPLC traces with peaks corresponding to the substrate and the two chiral products, retention times *t*_S_ = 9.7 min and *t*_R_ = 10.6 min, increasing over time during catalysis and the signal for the substrate, *t*_sub_ = 13.0 min, decreasing over time.

The catalytic activities of complexes **3x** were evaluated for the transfer hydrogenation of dehydrosalsolidine (Figure 9a) at 40 °C in aqueous buffer at pH 5.8, using sodium formate as the hydride source and a catalyst loading of 0.25 mol %.^33^ Samples were taken from the reaction solution at selected time points and quenched in a solution of glutathione, which deactivated the catalyst, halting the reaction. The quenched samples were then analyzed by high-performance liquid chromatography (HPLC) (Figure 9b).

**Figure 10:**
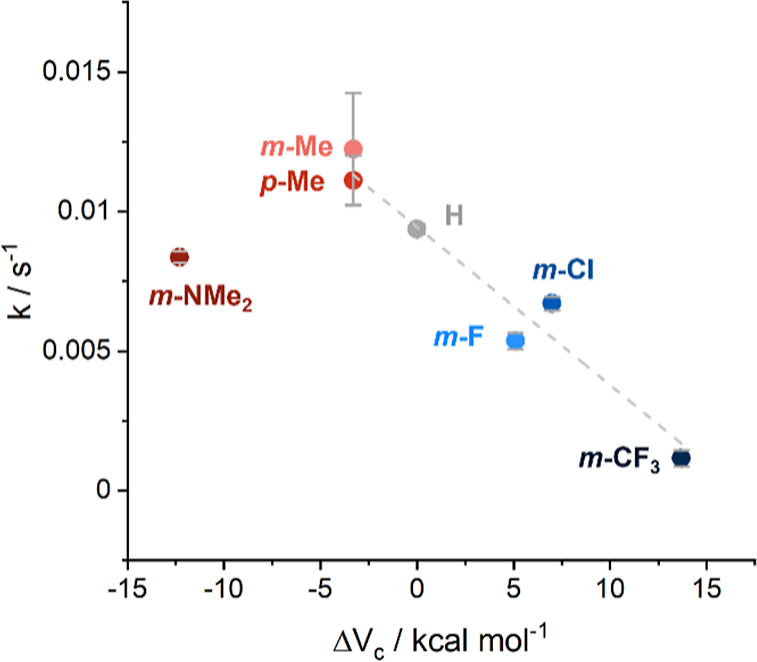
Plot of the first-order rate constants for each catalyst **3a**–**3g** against the parameter Δ*V*_c_, a theoretical measure of the electron-donating or -withdrawing ability of the pyridine substituent. The dashed line indicates a linear fit of the points, excluding *m-*Cl and *m-*NMe_2_.

The rates of the reaction showed a distinct trend across the series of catalysts, with catalysts bearing electron-donating substituents catalyzing the imine reduction better than the derivatives with electron-withdrawing groups. The strongly electron-withdrawing trifluoromethyl group significantly reduced the catalytic ability of the corresponding complex **3a**. The methyl-substituted derivatives **3e** and **3f** proved to be the best catalysts, despite the dimethylamino-substituent being a more strongly electron-donating group (Figure 10 and Table S2). The ability of the NMe_2_ group to accept a proton, thus converting the substituent to an electron-withdrawing group, could account for the lower catalytic activity; however, in the catalytic buffer (pH 5.8), a significant degree of protonation is unlikely. While the p*K*_a_ value for the exact structure could not be found in the literature, the conjugate acid of the closely related *N*,*N*-dimethylaniline has a reported p*K*_a_ of 5.07 at 25 °C in water^58^ and the more electron-withdrawing nature of the pyridine ring compared to a phenyl ring is expected to decrease the p*K*_a_, as will the effect of the coordination of the pyridine nitrogen donor to iridium. Additionally, the p*K*_a_ value will be lower under the catalytic conditions employed (40 °C) as p*K*_a_ decreases with increasing temperature. Since our catalytic reaction is buffer controlled at pH 5.8, it is expected that only a very small proportion, if any, of the protonated species may be present and so does not account for the significantly reduced catalytic activity.

As expected, no enantioselectivity was observed for any of the racemic catalysts.

**Figure 11:**
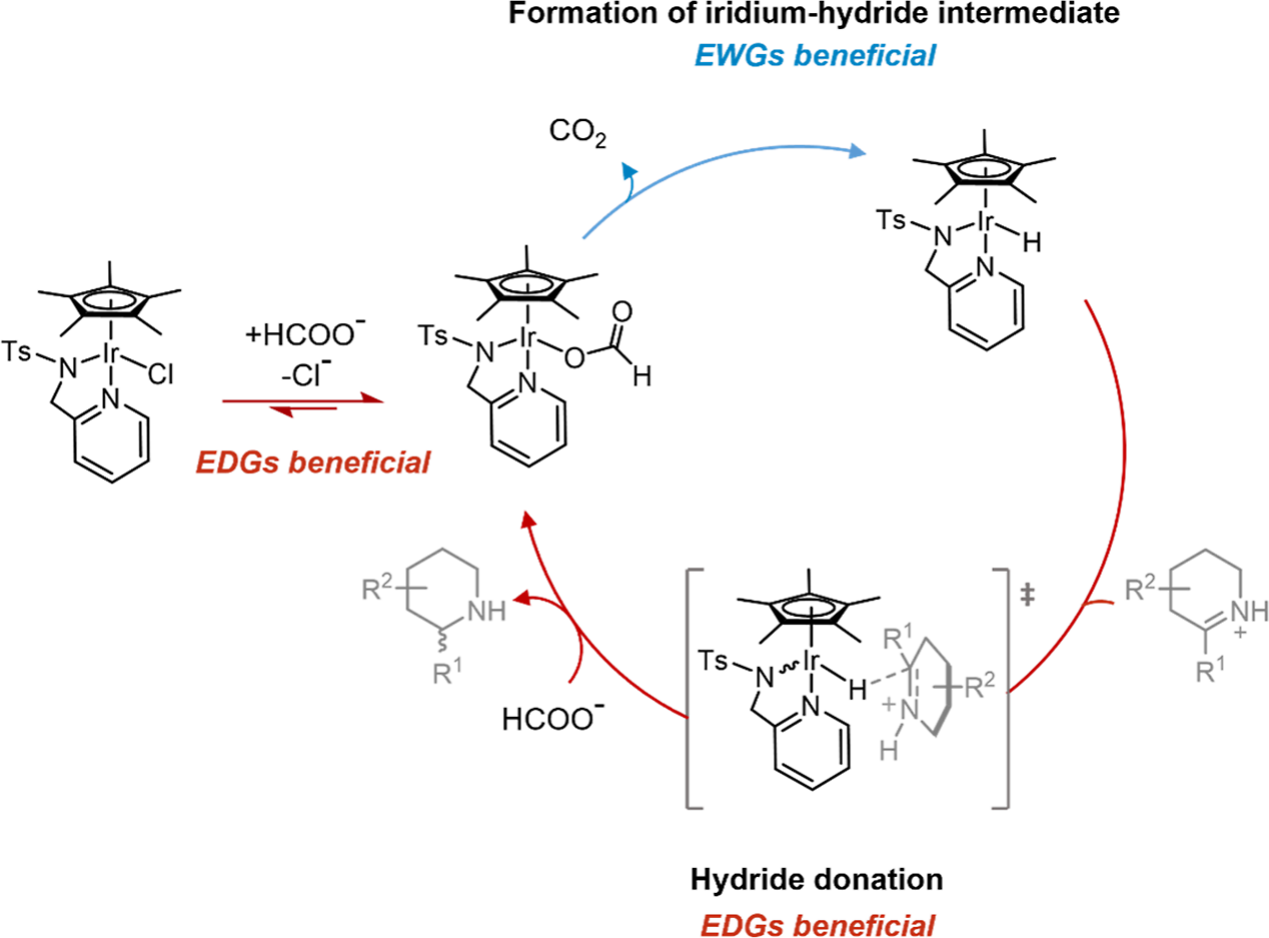
Adapted catalytic cycle for the transfer hydrogenation of imines by [Cp*Ir((pyridinylmethyl)sulfonamide)Cl]-derived catalysts under acidic aqueous conditions.

The behavior of catalysts **3a**–**3g** in water (Supporting Information, Section S9) has led us to propose a catalytic cycle (Figure 11) that differs from the transfer hydrogenation mechanism that dominates in organic solvents (Figure 2). While it was possible to isolate the 16-electron state following addition of silver hexafluorophosphate to remove the chlorido ligand, as confirmed by NMR spectroscopy (Figure S9) and X-ray crystallography (Figure S11), we found no evidence for the presence of the 16-electron intermediate in aqueous solution upon dissolving complex **3d** in a D_2_O/methanol-*d*_4_ mixture. The distinct proton NMR spectrum of the 16-electron intermediate was not observed. A hydrolyzed 18-electron species in which a water molecule occupies one coordination site was also not apparent. This was surprising since aquated species have been proposed for a number of similar catalysts in water.^59,60^ In order to investigate whether the primary species in solution is the 18-electron species with a chlorido- or water ligand-bound, ^1^H NMR spectra of complex **3d** were recorded in the presence of increasing amounts of NaCl to drive the equilibrium of the two species toward formation of the chloride-bound species, as has previously demonstrated in a number of other studies.^61^ All spectra show only one set of signals, and the small shifts observed in the resonances are consistent with the gradual increase in ionic strength, suggesting that the same species is present in all samples from the NaCl-free solution to the saturated NaCl solution (Figure S10), unless very fast ligand exchange occurs under these conditions. These observations suggest that compound **3d** is stable in water. Interestingly, **3d** crystallized from the saturated NaCl solution and the crystal structure confirmed that the chlorido ligand remained coordinated to the iridium center, as seen in the compound previously isolated from organic solvents.^62^ A water of crystallization, which is also present in the structure, does not interact with the metal center (Figure S11). We therefore propose that instead of the formation of the 16-electron species at the initiation of the catalytic cycle, the displacement of the chlorido ligand takes place in the presence of another anionic ligand, in this case, formate (Figure 11). This is consistent with our spectroscopic study which suggested that a change in stereochemistry at iridium occurs by an associative mechanism rather than dissociative. Furthermore, the results from our NMR spectroscopic study informed which steps in the mechanism are likely to be favored by electron-donating and electron-withdrawing groups, which are indicated in Figure 11.

Plotting the pseudo first-order rate constants for each catalyst against conventional Hammett parameters (σ_m_ and σ_p_) did not produce a clear linear trend (see the Supporting Information). Instead, using calculated parameters, Δ*V*_c_, produces a better-defined correlation with our results (Figure 10). It was also noted that, while the unsubstituted catalyst **3d** and catalysts **3a**–**3c** bearing electron-withdrawing substituents produce good linear correlations for pseudo first-order rate plot (ln([substrate]) against time), catalysts **3e**–**3g** showed a systematic deviation of data points from the line of the linear fit, tending more toward zero-order relationships (Supporting Information; Figures S5 and S6). This suggests a change in the rate-determining step in the catalytic cycle depending on the electron-donating or -withdrawing nature of the substituent on the pyridine ring. For catalysts with electron-withdrawing substituents, a pseudo first-order rate constant indicates that hydride donation is the rate-determining step. Increasing the electron-donating ability of the substituent is expected to improve the rate of this step, according to our variable-temperature spectroscopic analysis; however, at some point, further increasing the electron-donating ability of the substituent causes a change in the rate-determining step to β-hydride elimination, which is zero-order with respect to the substrate.

This change in rate-determining step explains the large deviation from the trend for catalyst **3g** with a *meta*-dimethylamine substituent, where the limiting rate of β-hydride elimination results in significantly poorer catalytic rate than that might be expected from our variable-temperature NMR study. Comparable two-phase Hammett behavior, with a change in the rate-determining step, has previously been reported for similar iridium catalysts for dehydrogenation^24^ and ruthenium catalysts for C–H functionalization.^42^

## Summary and Conclusions

A series of three-legged piano stool complexes with (pyridinylmethyl)sulfonamide ligands were synthesized with the aim of modulating the activity of Cp*Ir-based transfer hydrogenation catalysts via the addition of electron-donating and -withdrawing groups to the pyridine ring. Variable-temperature ^1^H NMR spectroscopic investigations indicated a clear positive correlation between the electron-donating ability of the substituent on the (pyridinylmethyl)sulfonamide ligand and the rate of stereoinversion at the chiral iridium center. Similarly, a positive correlation was observed between the electron-donating ability of the pyridine substituent and the catalytic activity of the respective imine reduction catalyst, as long as the hydride donation from the iridium-hydride intermediate remains rate-determining. The strongly electron-donating dimethylamino substituent, however, caused a change in the rate-determining step, from hydride-donation to hydride-complex formation, and hence deviated from this trend by leading to a decrease in catalytic activity. Interestingly, it was also found that the experimental first-order rate constants did not produce an acceptable correlation with conventional Hammett parameters, indicating that these parameters are poor descriptors for this pyridyl-based ligand system. Instead, the theoretical parameter Δ*V*_c_ produced a better trend.

Overall, these results demonstrate that for the reduction of imines in water by [Cp*Ir((pyridinylmethyl)sulfonamide)Cl]-derived catalysts, there is a fine balance between the relative rates of hydride complex formation and hydride donation. Variable-temperature NMR emerged as a useful tool in predicting the relative rates of the hydride donation step; however, this does not apply to cases where the hydride donation is not rate determining. Nevertheless, the current study has revealed clear trends in stereoinversion barriers, catalytic reaction rates, and theoretically derived Δ*V*_c_ parameters that may serve as guides in the optimization of the catalytic transfer hydrogenation rates of inorganic imine reduction catalysts, artificial metalloenzymes, and metallodrug candidates that function in aqueous media.

## Supporting Information Available

Synthetic methods and characterization data for ligands and complexes, variable-temperature NMR data, additional catalytic data and plots, and general materials and HPLC methods (PDF)
