# Supplementary material for: Effect of Ligand Substituents on Spectroscopic and Catalytic Properties of Water-Compatible Cp*Ir-(pyridinylmethyl)sulfonamide-Based Transfer Hydrogenation Catalysts
Source: Inorg Chem. 2024 Feb 12;63(8):3815–23. doi: 10.1021/acs.inorgchem.3c04040 (PMC10900292; doi:10.1021/acs.inorgchem.3c04040)
Supplement: Supplementary file 1 — ic3c04040_si_001.pdf [file ic3c04040_si_001.pdf]

Supporting Information

The effect of ligand substituents on spectroscopic and catalytic properties of water-compatible Cp*Ir-(pyridinylmethyl)sulfonamide-based transfer hydrogenation catalysts

*Rosalind L. Booth, Adrian C. Whitwood, Anne-K. Duhme-Klair**

Department of Chemistry, University of York, York, YO10 5DD, United Kingdom

*anne.duhme-klair@york.ac.uk

CONTENTS

1. MATERIALS 2

2. INSTRUMENTATION 2

3. SYNTHESIS OF LIGANDS 3

4. SYNTHESIS OF COMPLEXES 7

5. VARIABLE TEMPERATURE NMR SPECTRA OF COMPLEXES **3a-3g** 11

6. LINE SHAPE ANALYSIS 12

7. CATALYTIC ACTIVITY TESTING 14

8. ALTERNATIVE HAMMETT PLOTS 18

9. SPECTROSCOPIC EXPERIMENTS TO IDENTIFY THE WATER-STABLE SPECIES (FOR RATIONALISING THE PROPOSED CATALYTIC CYCLE) 19

10. X-RAY CRYSTALLOGRAPHY DATA 21

11. ^{1}H AND ^{13}C NMR SPECTRA 24

12. REFERENCES 39

1. MATERIALS

Materials were obtained from the following commercial suppliers: Acros, Alfa-Aesar, Fluorochem, Fisher Scientific, Sigma-Aldrich, Tokyo Chemical Industry Ltd. Analytical thin layer chromatography (TLC) was performed using Merck silica gel 60 F253 aluminum-backed plates using specific solvent systems and visualized under an ultraviolet lamp. Column chromatography was carried out using Fluka Silica, pore size 60 Å, 220-440 mesh, 35-75 μm.

2. INSTRUMENTATION

Characterisation data for NMR including ^{1}H, ^{13}C, COSY, DEPT135, HMQC and HMBC spectra were recorded on Jeol ECS 400 and ECX 400 MHz instruments (400 MHz for ^{1}H and 101 MHz for ^{13}C) at ambient temperature, unless otherwise stated. ^{13}C NMR spectra are proton decoupled. Variable temperature NMR experiments were carried out by H. Fish and were measured using a Bruker AV500b instrument (500 MHz for ^{1}H). ^{1}H NMR data are reported as follows: chemical shift (number of protons, multiplicity, coupling constants, *assignment*). ^{13}C NMR data are reported as follows: chemical shift (*assignment*). Chemical shifts are reported in parts per million (ppm), relative to the residual solvent peaks to the nearest 0.01 ppm for ^{1}H NMR spectra and 0.1 ppm for ^{13}C NMR spectra. Multiplicity is reported as follows: s=singlet, d=doublet, t=triplet, q=quartet, m=multiplet, br=broad. Coupling constants (J) are given in Hz and are quoted to the nearest 0.5 Hz. All NMR spectra were processed MestReNova analysis software.

High-resolution ESI mass spectra were recorded on a Bruker microTOF electrospray mass spectrometer and liquid injection field desorption ionisation (LIFDI) on a Waters GCT Premier TOF mass spectrometer by K. Heaton, R. Cercola and A. Lopez. Melting points were measured using a Stuart Scientific SMP3 melting point apparatus. Infrared spectra were recorded using a Perkin Elmer FT-IR spectrum 2 spectrometer at ambient temperature. Data is reported as peaks of interest. Elemental analysis was measured using an Exeter CE-440 elemental analyzer, carried out by G. McAllister and S. Hicks.

Non-chiral analytical HPLC was performed using an Athena C18-WP column (100 Å, 4.6 x 25 mm, CNW). Chiral analytical HPLC was performed with a Lux Cellulose-4 column (Phenomenex, 250 x 4.6 mm, 5 μm). Measurements were taken using an Agilent 1260 Infinity II Quaternary System equipped with a 1260 Quaternary pump G7111B, 1260 Vial sampler, G7129A, multicolumn thermostat G7116A oven and a 1260 multiwavelength detector G7165A.

3. SYNTHESIS OF LIGANDS

GENERAL METHOD FOR SYNTHESIS OF PYRIDINYLMETHYLSULFONAMIDE LIGANDS

The method was adapted from the literature.[1]

The relevant substituted 2-(aminomethyl)pyridine (1 equiv.) was dissolved in anhydrous dichloromethane with an appropriate base (triethylamine or *N, N*-diisopropylethylamine) (2 equiv.). A separate solution of 4-toluenesulfonyl chloride (1.1 equiv.) in dichloromethane was added dropwise to the 2-(aminomethyl)pyridine solution at 0 °C. Following complete addition of the 4-toluenesulfonyl chloride solution (final volume of DCM approximately 30 mL/mol of substituted 2-(aminomethyl)pyridine), the reaction mixture was stirred at room temperature for 16 hours. The solution was concentrated to approximately one-third volume before being washed three time with distilled water and once with brine. The organic layer was dried over $MgSO_4$ and the remaining solvent removed in vacuo. The crude product was purified by column chromatography.

Ligand **a**:

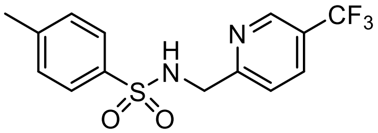

Column chromatography conditions: 2:3 ethyl acetate: petroleum ether 40-60 °C

Yield: 72%. ^{1}H NMR (400 MHz, DMSO-d_6) δ: 8.76-8.73 (1H, m), 8.34 (1H, t, J^3= 6.5 Hz), 8.08 (1H, dd, J^3= 8.5 Hz, J^4= 2.5 Hz), 7.59 (2H, d, J^3= 8.0 Hz), 7.52 (1H, d, J^3= 8.5 Hz), 7.28 (2H, d, J^3= 8.0 Hz), 4.15 (2H, d, J^3= 6.5 Hz), 2.31 (3H, s). ^{13}C{^{1}H} NMR (100 MHz, DMSO-d_6) δ: 162.5, 146.0, 143.2, 138.1, 134.6, 130.1, 127.1, 124.3 (d, $J^1_{C\text{-}F}$= 272 Hz), 124.1 (q, $J^2_{C\text{-}F}$= 32 Hz), 122.5, 48.2, 21.4. IR (cm^{-1}): 3160 (br), 2981 (br), 1327 (m). HRMS (ESI): $[M+H]^+$ calcd. meas. m/z= 331.0723; m/z= 331.0711, mean error 2.8 ppm; $[M+Na]^+$ calcd. m/z= 353.0542; meas. m/z= 353.0545, mean error -1.0 ppm. Elemental analysis calcd. for [$C_{13}H_{14}N_2O_2S$]: %C 50.91, %H 3.97, %N 8.48; meas. for [$C_{13}H_{14}N_2O_2S$]: %C 50.88, %H 4.33, %N 8.40.

Ligand **b**:

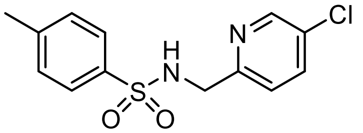

Column chromatography conditions: 2:3 ethyl acetate: petroleum ether 40-60 °C

Yield: 41%. ^{1}H NMR (400 MHz, DMSO-d_6) δ: 8.42 (1H, dd, J^4= 2.5 Hz, J^5= 0.5 Hz), 8.21 (1H, t, J^3= 6.0 Hz), 7.81 (1H, dd, J^3= 8.5 Hz, J^4= 2.5 Hz), 7.60 (2H, d, J^3= 7.5 Hz), 7.36-7.28 (3H, m), 4.02 (2H, d, J^3= 6.0 Hz), 2.33 (3H, s). ^{13}C{^{1}H} NMR (100 MHz, DMSO-d_6) δ: 156.5, 147.7, 143.2, 138.2, 137.0, 130.1, 130.1, 127.1, 123.7, 47.8, 21.5. IR (cm^{-1}): 3128 (br), 2877 (w), 1329 (m), 1158 (m). HRMS (ESI): [M+H]$^+$ calcd. m/z= 297.0459, meas. m/z= 297.0466, mean error -1.4 ppm; [M+Na]$^+$ calcd. m/z= 319.0278; meas. m/z= 319.0284, mean error -2.2 ppm; [M+K]$^+$ calcd. m/z= 335.0018; meas. m/z= 335.0013, mean error -0.4 ppm. Elemental analysis calcd. for [$C_{13}H_{13}ClN_2O_2S$]: %C 52.61, %H 4.42, %N 9.44; meas. for [$C_{13}H_{13}ClN_2O_2S$]: %C 52.76, %H 4.84, %N 9.39.

Ligand **c**:

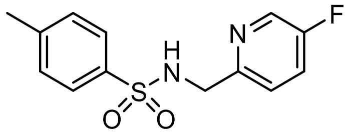

Column chromatography conditions: 2:3 ethyl acetate: petroleum ether 40-60 °C

Yield: 67%. ^{1}H NMR (400 MHz, DMSO-d_6) δ: 8.38 (1H, d, J^3= 3.0 Hz), 8.20 (1H, t, J^3= 6.0 Hz), 7.66-7.58 (3H, m), 7.37 (1H, dd, J^3= 8.5 Hz, $J^4_{H\text{-}F}$= 4.5 Hz), 7.32 (2H, d, J^3= 8.0 Hz), 4.02 (2H, d, J^3= 6.0 Hz), 2.33 (3H, s). ^{13}C{^{1}H} NMR (100 MHz, DMSO-d_6) δ: 158.8 (d, $J^1_{C\text{-}F}$= 252.0 Hz), 154.2 (d, $J^4_{C\text{-}F}$= 3.5 Hz), 143.2, 138.2, 137.1 (dd, $J^2_{C\text{-}F}$= 23.5 Hz, 18.0 Hz), 130.1, 127.1, 124.1 (dd, $J^2_{C\text{-}F}$= 19.0 Hz, 11.5 Hz), 123.7 (d, $J^3_{C\text{-}F}$= 4.0 Hz), 47.8 (t, $J^5_{C\text{-}F}$= 9.0 Hz), 21.5. IR (cm^{-1}): 3103 (br), 2931 (br), 1515 (m), 1324 (m). HRMS (ESI): [M+H]$^+$ calcd. m/z= 281.0755, meas. m/z= 281.0759, mean error -1.4 ppm; [M+Na]$^+$ calcd. m/z= 303.0574; meas. m/z= 303.0580, mean error -0.8 ppm; [M+K]$^+$ calcd. m/z= 319.0313; meas. m/z= 319.0308, mean error 0.6 ppm. Elemental analysis calcd. for [$C_{13}H_{13}FN_2O_2S$]: %C 55.70, %H 4.67, %N 9.99; meas. for [$C_{13}H_{13}FN_2O_2S$]: %C 55.73, %H 4.96, %N 10.01.

Ligand **d**:

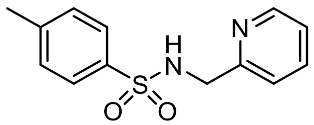

Column chromatography conditions: 1:1 ethyl acetate: petroleum ether 40-60 °C

Yield: 50%. ^{1}H NMR (400 MHz, DMSO-d_6) δ: 8.39 (1H, ddd, J^3= 5.0 Hz, , J^4= 2.0 Hz, J^5= 1.0 Hz), 8.16 (1H, br), 7.68 (1H, apparent td, J^3= 7.5 Hz, J^4= 2.0 Hz), 7.66 (2H, d, J^3= 8.0 Hz), 7.36-7.29 (3H, m), 7.19 (1H, ddd, , J^3= 7.5 Hz, J^3= 5.0 Hz, J^4= 1.0 Hz), 4.03 (2H, s), 2.33 (3H, s). ^{13}C{^{1}H} NMR (100 MHz, DMSO-d_6) δ: 157.7, 149.3, 143.2, 138.2, 137.2, 130.1, 127.1, 122.9, 122.1, 48.5, 21.5. IR (cm^{-1}): 3053 (br), 2880 (br), 1599 (m), 1328 (m). HRMS (ESI): [M+H]$^+$ calcd. m/z= 263.0849; meas. m/z= 263.0847, mean error 0.8 ppm; [M+Na]$^+$ calcd. m/z= 285.0668;

meas. m/z= 285.0665, mean error 1.8 ppm. Elemental analysis calcd. for [$C_{13}H_{14}N_2O_2S$]: %C 59.52, %H 5.38, %N 10.68; meas. for [$C_{13}H_{14}N_2O_2S$]: %C 57.65, %H 5.44, %N 10.46.

Ligand **e**:

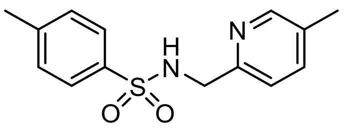

Column chromatography conditions: 3:2 ethyl acetate: petroleum ether 40-60 °C

Yield: 62%. ^{1}H NMR (400 MHz, DMSO-d_6) δ: 8.23-8.8.20 (1H, m), 8.09 (1H, br), 7.63 (2H, d, J^3= 8.0 Hz), 7.49 (1H, dd, J^3= 8.0 Hz, J^4= 2.0 Hz), 7.32 (2H, d, J^3= 8.0 Hz), 7.19 (1H, d, J^3= 8.0 Hz), 3.95 (2H, br), 2.34 (3H, s), 2.20 (3H, s). ^{13}C{^{1}H} NMR (100 MHz, DMSO-d_6) δ: 154.8, 149.4, 143.1, 138.2, 137.5, 132.0, 130.1, 127.1, 121.7, 48.2, 21.5, 18.1. IR (cm^{-1}): 3043 (br), 2846 (br), 1313 (m), 1303 (m). HRMS (ESI): [M+H]$^+$ calcd. m/z= 277.1005, meas. m/z= 277.1004, mean error 0.6 ppm; [M+Na]$^+$ calcd. m/z= 299.0825; meas. m/z= 299.0823, mean error -1.5 ppm. Elemental analysis calcd. for [$C_{14}H_{16}N_2O_2S$]: %C 60.85, %H 5.84, %N 10.14; meas. for [$C_{14}H_{16}N_2O_2S$]: %C 60.77, %H 6.08, %N 10.17.

Ligand **f**:

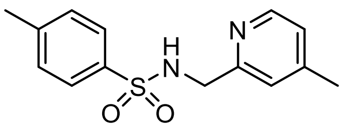

Column chromatography conditions: 3:2 ethyl acetate: petroleum ether 40-60 °C

Yield: 68%. ^{1}H NMR (400 MHz, DMSO-d_6) δ: 8.23 (1H, dd, J^3= 5.0 Hz, J^5= 0.5 Hz), 8.11 (1H, br), 7.62 (2H, d, J^3= 8.0 Hz), 7.32 (2H, d, J^3= 8.0 Hz), 7.05 (1H, s), 7.02 (1H, d, J^3= 5.0 Hz), 3.97 (2H, s), 2.33 (3H, s), 2.20 (3H, s). ^{13}C{^{1}H} NMR (100 MHz, DMSO-d_6) δ: 157.4, 149.0, 147.8, 143.2, 138.3, 130.1, 127.1, 123.7, 122.8, 48.4, 21.5, 21.0. IR (cm^{-1}): 3054 (br), 2853 (br), 1610 (m), 1325 (s), 1158 (s). HRMS (ESI): [M+H]$^+$ calcd. m/z= 277.1005, meas. m/z= 277.1007, mean error -1.3 ppm; [M+Na]$^+$ calcd. m/z= 299.0825; meas. m/z= 299.0828, mean error -0.8 ppm; [M+K]$^+$ calcd. m/z= 315.0564; meas. m/z= 315.0565, mean error -0.8 ppm. Elemental analysis calcd. for [$C_{14}H_{16}N_2O_2S$]: %C 60.85, %H 5.84, %N 10.14; meas. for [$C_{14}H_{16}N_2O_2S$]: %C 60.47, %H 5.84, %N 9.94.

Ligand **g**:

(5-Dimethylaminepyridin-2-yl)methylamine was synthesized by a method adapted from the literature:[2]

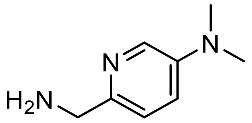

(5-Chloropyridin-2-yl)methylamine (0.255 g, 1.8 mmol) and dimethylamine hydrochloride salt (2.208 g, 27 mmol) was dissolved in water (6 mL) in a microwave vessel. The solution was placed on ice and sodium hydroxide (1.802 g, 45 mmol) was added gradually. The solution was stirred well before being transferred to a CEM Discover microwave reactor and irradiated for 20 minutes intervals with conditions restricted to 190 °C, 250 psi. The reaction was stopped once a brown precipitate started to appear. Water was removed by evaporation under vacuum. The remaining residue was loaded onto a silica plug and eluted with 8% methanol in chloroform. The crude product was not purified further.

Yield: 41%. ^{1}H NMR (400 MHz, DMSO-d$_6$) δ: 8.05 (1H, d, J^4= 3.0 Hz), 7.11 (1H, d, J^3 = 8.5 Hz), 6.95 (1H, dd, J^3= 8.5 Hz, J^4= 3.0 Hz), 3.92 (2H, s), 2.93 (6H, s). HRMS (ESI): $[M+H]^+$ calcd. m/z= 152.1182, meas. m/z= 152.1186, mean error -2.9 ppm.

The dimethylamine-substituted ligand was then prepared by the general method using the synthesized (5-dimethylaminepyridin-2-yl)methylamine.

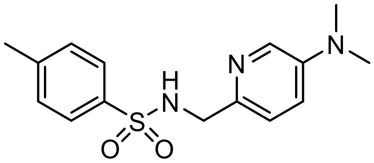

Column chromatography conditions: 1:1 ethyl acetate: petroleum ether 40-60 °C

Yield: 21%. ^{1}H NMR (400 MHz, DMSO-d$_6$) δ: 7.94 (1H, t, J^3= 6.0 Hz), 7.88 (1H, d, J^4= 3.0 Hz), 7.61 (2H, d, J^3= 8.0 Hz), 7.31 (2H, d, J^3= 8.0 Hz), 7.06 (1H, d, J^3= 8.5 Hz), 6.98 (1H, dd, J^3= 8.5 Hz, J^4= 3.0 Hz), 3.87 (2H, d, J^3= 6.0 Hz), 2.84 (6H, s), 2.33 (3H, s). ^{13}C{^{1}H} NMR (100 MHz, DMSO-d$_6$) δ: 145.8, 144.5, 142.9, 138.3, 134.1, 130.0, 127.1, 122.3, 119.9, 48.1, 40.3, 21.5. HRMS (ESI): $[M+H]^+$ calcd. m/z= 306.1271, meas. m/z= 306.1274, mean error -1.3 ppm; $[M+Na]^+$ calcd. m/z= 328.1090; meas. m/z= 328.1094, mean error -1.9 ppm.

4. SYNTHESIS OF COMPLEXES

$[Cp^*IrCl_2]_2$ was prepared based by a method modified from the literature.[3]

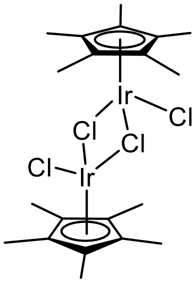

Iridium(III) chloride trihydrate (0.846 g, 2.40 mmol) was dissolved in degassed anhydrous methanol (25 mL) under a nitrogen atmosphere. 1, 2, 3, 4, 5-Pentamethylcyclopentadiene (0.90 mL, 5.75 mmol) was added, and the reaction mixture heated to reflux for 36 hours. The mixture was cooled to room temperature and the product isolated as a bright red/orange precipitate by filtration. The isolated product was rinsed with ice cold methanol. Further product was recovered by reducing the volume of the filtrate and cooling over ice.

Yield: 85%. ^{1}H NMR (400 MHz, $CDCl_3$) δ: 1.59 (s, *H1*). $^{13}C\{^{1}H\}$ NMR (100 MHz, $CDCl_3$) δ: 86.3, 9.5. IR (cm^{-1}): 2988 (w), 2967 (w), 2912 (w), 1448 (m). HRMS (LIFDI): $[M\text{-}Cl]^+$ calcd. m/z= 398.01746, meas. m/z= 398.01661, mean error 2.13 ppm. Elemental analysis calcd. for $[C_{20}H_{30}Cl_4Ir_2]$: %C 30.15, %H 3.80, %N 0.00; meas. for $[C_{13}H_{14}N_2O_2S]$: %C 29.82, %H 3.88, %N 0.00.

GENERAL METHOD FOR THE SYNTHESIS OF COMPLEXES

Method was adapted from literature.[1]

$[Cp^*IrCl_2]_2$ (1 equiv.) and the corresponding ligand (2 equiv.) were dissolved in dry DCM (30 mL/mol $[Cp^*IrCl_2]_2$). NaOH (2 M in methanol, 2 equiv.) was added and the solution sonicated for 20 minutes. Distilled water (30 mL/mol $[Cp^*IrCl_2]_2$) was added, and the mixture stirred vigorously for 30 seconds. The organic layer was removed by syringe and the volume reduced to approximately 1/3rd volume in vacuo. The product was isolated by slow addition of diethyl ether, promoting the formation of the product as orange crystals, which were isolated by filtration.

Complex **3a**:

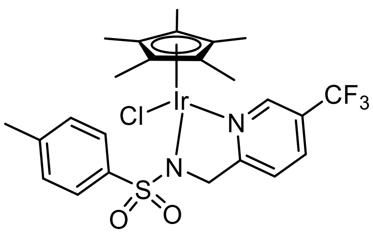

Yield: 56%. ^{1}H NMR (400 MHz, $CDCl_3$) δ: 8.79 (1H, s), 7.91 (1H, dd, J^3= 8.5 Hz, J^4= 2.0 Hz), 7.88 (2H, d, J^3= 8.0 Hz), 7.34 (1H, d, J^3= 8.5 Hz), 7.10 (2H, d, J^3= 8.0 Hz), 4.94 and 4.66 (2H, 2 x d, J^2= 18.0 Hz), 2.28 (3H, s), 1.72 (15H, s). $^{13}C\{^1H\}$ NMR (100 MHz, $CDCl_3$) δ: 168.7, 148.2, 141.0, 139.5, 134.7, 129.0, 128.3, 128.0, 122.2 (d, $J^1{}_{C\text{-}F}$= 272.5 Hz), 120.8, 87.1, 57.9, 21.5, 9.6. HRMS (ESI): $[M-Cl]^+$ calcd. m/z= 657.1369; meas. m/z= 657.1402, mean error -2.4 ppm. Elemental analysis calcd. for [$C_{24}H_{27}ClF_3IrN_2O_2S$]: %C 41.64, %H 3.93, %N 4.05; meas. for [$C_{24}H_{27}ClF_3IrN_2O_2S$]: %C 41.30, %H 3.94, %N 3.98.

Complex **3b**:

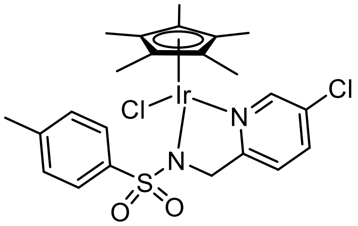

Yield: 99%. ^{1}H NMR (400 MHz, $CDCl_3$) δ: 8.48 (1H, dd, J^4= 2.0 Hz, J^5= 1.0 Hz), 7.87 (2H, d, J^3= 8.0 Hz), 7.64 (1H, dd, J^3= 8.5 Hz, J^4= 2.0 Hz), 7.14 (1H, dd, J^3= 8.5 Hz, J^5= 1.0 Hz), 7.09 (2H, d, J^3= 8.0 Hz), 4.83 and 4.54 (2H, 2 x d, J^2= 17.5 Hz), 2.28 (3H, s), 1.72 (15H, s). $^{13}C\{^1H\}$ NMR (100 MHz, $CDCl_3$) δ: 163.1, 149.8, 140.8, 139.7, 137.9, 132.2, 128.9, 128.3, 120.9, 86.9, 57.3, 21.4, 9.6. HRMS (ESI): $[M-Cl]^+$ calcd. m/z= 623.1105; meas. m/z= 623.1107, mean error -3.4 ppm. Elemental analysis calcd. for [$C_{23}H_{27}Cl_2IrN_2O_2S$]: %C 41.94, %H 4.13, %N 4.25; meas. for [$C_{23}H_{27}Cl_2IrN_2O_2S$]: %C 41.50, %H 4.22, %N 4.86.

Complex **3c**:

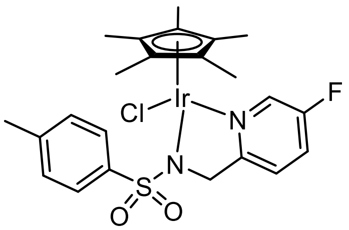

Yield: 48%. ^{1}H NMR (400 MHz, $CDCl_3$) δ: 8.41 (1H, apparent t, J^3= 2.5 Hz), 7.87 (2H, d, J^3= 8.0 Hz), 7.44 (1H, ddd, J^3= 9.0 Hz, J^3= 6.5 Hz, J^4= 2.5 Hz), 7.18 (1H, dd, J^3= 9.0 Hz, J^4= 5.0 Hz),

7.08 (2H, d, J^3= 8.0 Hz), 4.81 and 4.53 (2H, 2 x br d, J^2= 16.5 Hz), 2.28 (3H, s), 1.73 (15H, s). $^{13}C\{^1H\}$ NMR (100 MHz, $CDCl_3$) δ: 161.0 (d, J^4= 3.5 Hz), 160.4, 140.8, 139.8, 139.6 (d, J^2= 23 Hz), 128.9, 128.3, 125.5 (d, J^2= 19 Hz), 121.0 (d, J^3= 5.5 Hz), 86.9, 57.1, 21.4, 9.7. HRMS (ESI): $[M-Cl]^+$ calcd. m/z= 607.1401; meas. m/z= 607.1428, mean error -1.7 ppm. Elemental analysis calcd. for [$C_{23}H_{27}ClFIrN_2O_2S$]: %C 43.02, %H 4.24, %N 4.36; meas. for [$C_{23}H_{27}ClFIrN_2O_2S$]: %C 43.07, %H 4.37, %N 4.17.

Complex **3d**:

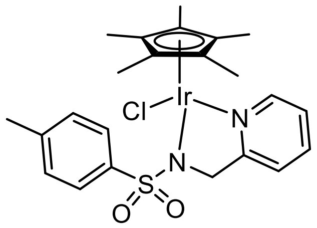

Yield: 64%. ^1H NMR (400 MHz, $CDCl_3$) δ: 8.52 (1H, dq, J^3= 5.5 Hz, J^4= 0.5 Hz), 7.87 (2H, d, J^3= 8.0 Hz), 7.65 (1H, td, J^3= 8.0 Hz, J^4= 1.5 Hz), 7.21 (1H, t, J^3= 6.5 Hz), 7.16 (1H, d, J^3= 8.0 Hz), 7.07 (2H, d, J^3= 8.0 Hz), 4.78 and 4.58 (2H, br), 2.27 (3H, s), 1.72 (15H, s). $^{13}C\{^1H\}$ NMR (100 MHz, $CDCl_3$) δ: 164.5, 151.2, 140.6, 139.8, 137.9, 128.9, 128.4, 124.7, 120.5, 86.6, 57.8, 21.5, 9.7. HRMS (ESI): $[M-Cl]^+$ calcd. m/z= 589.1495; meas. m/z= 589.1495, mean error -0.1 ppm. Elemental analysis calcd. for [$C_{23}H_{28}ClIrN_2O_2S$]: %C 44.26, %H 4.52, %N 4.49; meas. for [$C_{23}H_{28}ClIrN_2O_2S$]: %C 43.83, %H 4.61, %N 4.99.

Complex **3e**:

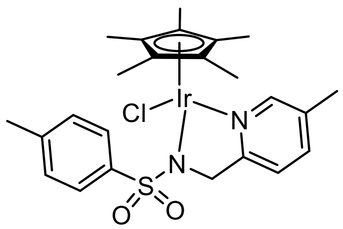

Yield: 64%. ^1H NMR (400 MHz, $CDCl_3$) δ: 8.32 (1H, s), 7.87 (2H, d, J^3= 8.0 Hz), 7.45 (1H, d, J^3= 8.0 Hz), 7.16-7.03 (3H, m), 4.76 and 4.50 (2H, br), 2.32 (3H, s), 2.27 (3H, s), 1.72 (15H, s). $^{13}C\{^1H\}$ NMR (100 MHz, $CDCl_3$) δ: 161.7, 151.0, 138.8, 134.6, 129.8, 128.9, 128.4, 126.6, 119.8, 86.5, 57.4, 21.4, 18.1, 9.7. HRMS (ESI): $[M-Cl]^+$ calcd. m/z= 603.1652; meas. m/z= 603.1670, mean error -1.4 ppm. Elemental analysis calcd. for [$C_{24}H_{30}ClIrN_2O_2S$]: %C 45.17, %H 4.74, %N 4.39; meas. for [$C_{24}H_{30}ClIrN_2O_2S$]: %C 44.07, %H 4.49, %N 4.14.

Complex **3f**:

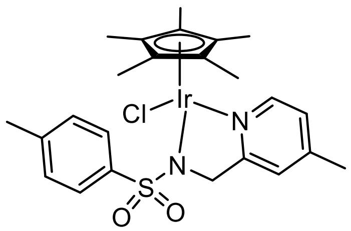

Yield: 74%. ^{1}H NMR (400 MHz, $CDCl_3$) δ: 8.34 (1H, d, J^3= 6.0 Hz), 7.89 (2H, d, J^3= 8.0 Hz), 7.08 (2H, d, J^3= 8.0 Hz), 7.01 (1H, d, J^3= 6.0 Hz), 6.98 (1H, s), 4.70 and 4.48 (2H, br), 2.32 (3H, s), 2.28 (3H, s), 1.72 (15H, s). $^{13}C\{^{1}H\}$ NMR (100 MHz, $CDCl_3$) δ: 163.9, 150.4, 150.1, 140.5, 139.9, 128.9, 128.4, 125.9, 121.1, 86.4, 57.6, 21.4, 21.1, 9.7. HRMS (ESI): $[M\text{-}Cl]^+$ calcd. m/z= 603.1652; meas. m/z= 603.1659, mean error -2.7 ppm. Elemental analysis calcd. for [$C_{24}H_{30}ClIrN_2O_2S$]: %C 45.17, %H 4.74, %N 4.39; meas. for [$C_{24}H_{30}ClIrN_2O_2S$]: %C 44.95, %H 4.89, %N 4.36.

Complex **3g**:

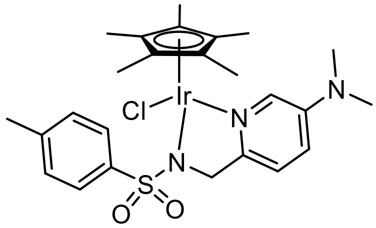

Note: It was not possible to isolate this complex by crystallization. Instead, the solvent was removed from the organic layer to produce an orange powder.

Yield: quant. ^{1}H NMR (400 MHz, $CDCl_3$) δ: 7.92 (1H, m), 7.88 (2H, d, J^3= 7.0 Hz), 7.07 (2H, d, J^3= 7.0 Hz), 6.93 (2H, m), 4.67 and 4.41 (2H, 2 x br), 2.96 (6H, s), 2.27 (3H, s), 1.73 (15H, s). $^{13}C\{^{1}H\}$ NMR (100 MHz, $CDCl_3$) δ: 151.3, 146.7, 140.4, 140.1, 135.2, 128.8, 128.4, 121.2, 119.8, 86.3, 56.8, 40.1, 21.4, 9.7. HRMS (ESI): $[M\text{-}Cl]^+$ calcd. m/z= 632.1917; meas. m/z= 632.1919, mean error -0.8 ppm. Elemental analysis calcd. for [$C_{25}H_{33}ClIrN_3O_2S$]: %C 45.00, %H 4.98, %N 6.30; meas. for [$C_{25}H_{33}ClIrN_3O_2S$]: %C 44.57, %H 4.79, %N 6.15.

5. VARIABLE TEMPERATURE NMR SPECTRA OF COMPLEXES **3a**-**3g**

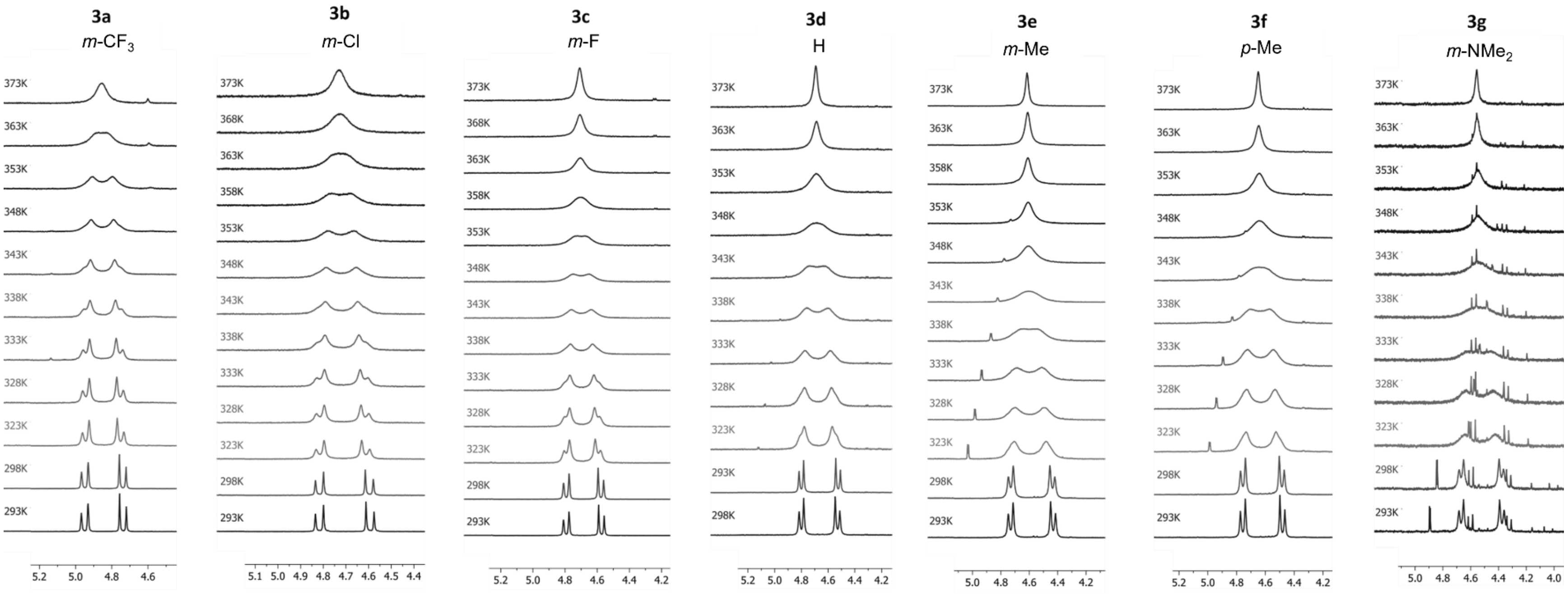

Figure S1. Variable temperature ^{1}H NMR spectra of complexes **3a**-**3g** in DMF-d_7.

6. LINE SHAPE ANALYSIS

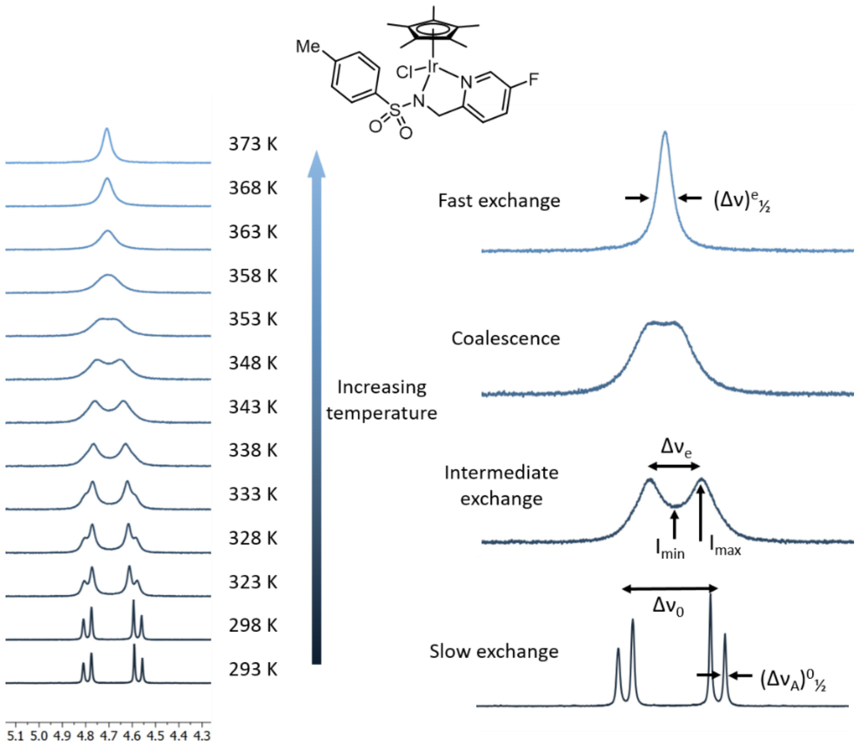

Figure S2. Variable temperature NMR spectra of complex **3c** indicating the different exchange regions considered in line shape analysis.

The approximations used for line shape analysis were as follows[4]:

Fast exchange:

$$k = \frac{\pi \Delta \nu_0{}^2}{\sqrt{2}} \frac{1}{\left[(\Delta \nu_{1/2})^e - (\Delta \nu_A)^0_{1/2}\right]}$$

Coalescence:

$$k = \frac{\pi \Delta \nu_0}{\sqrt{2}}$$

Intermediate exchange:

$$k = \frac{\pi}{\sqrt{2}} \left[(\Delta \nu_A)^e_{1/2} - (\Delta \nu_A)^0_{1/2}\right]$$

Slow exchange:

$$k = \frac{\pi \Delta \nu_0}{\sqrt{2}} \left(r + (r^2 - r)^{1/2}\right)^{-1/2}$$

$$\text{where } r = \frac{I_{max}}{I_{min}}$$

Table S1. Rates of interconversion derived from line shape analysis of ^{1}H NMR spectra of complexes **3a-3g** in DMF-d$_7$. Shading represents light grey = intermediate exchange region, mid grey = coalescence temperature and dark grey = fast exchange region. n.d. = values not determined.

**Temperature / K**	**Rate / s^{-1}**						
	***m*-CF$_3$**	***m*-Cl**	***m*-F**	**H**	***m*-Me**	***p*-Me**	***m*-NMe$_2$**
323	n.d.	n.d.	n.d.	n.d.	130.1	102.8	n.d.
328	n.d.	n.d.	n.d.	111.9	167.4	130.6	n.d.
333	n.d.	n.d.	n.d.	143.4	211.0	168.5	251.0
338	n.d.	n.d.	109.9	202.3	304.5	242.5	345.4
343	84.3	97.9	142.7	256.9	391.3	304.9	442.0
348	108.4	125.3	181.5	335.1	598.3	427.8	674.1
353	139.8	159.0	243.1	500.9	884.6	642.8	946.7
358	n.d.	204.2	351.5	n.d.	1279.9	n.d.	n.d.
363	235.0	248.2	523.1	1041.0	1778.6	1270.0	1918.2
368	n.d.	411.4	n.d.	n.d.	n.d.	n.d.	n.d.
373	515.5	598.4	n.d.	1917.7	3205.0	2329.9	3018.4

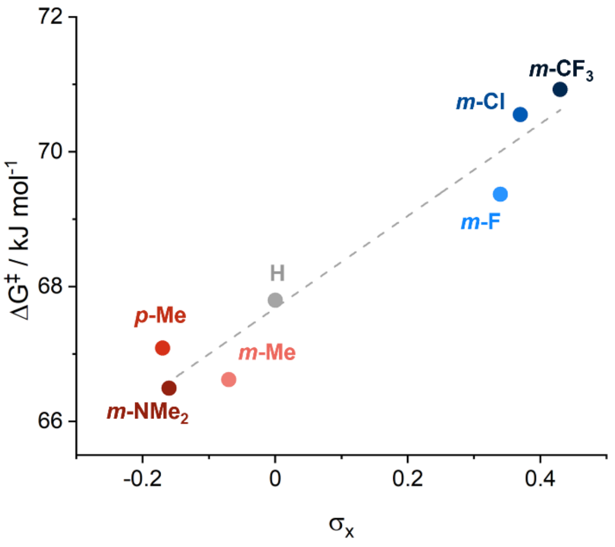

Figure S3. Plot of ΔG‡ (at 313 K) for each complex **3a-3g** against Hammett parameter σ_x, where σ_x represents either σ_m or σ_p as appropriate.

7. CATALYTIC ACTIVITY TESTING

METHOD

The following solutions were prepared:

- 0.0521 mol dm^{-3} 6,7-dimethoxy-1-methyl-3,4-dihydroisoquinoline in pH 6.0 buffer (0.6 mol dm^{-3} MES monohydrate, 3 mol dm^{-3} sodium formate)
- 0.00313 mol dm^{-3} catalyst in DMF

The solutions were incubated at 40 °C for 10 minutes. The reaction was initiated by the addition of 0.2 mL catalyst solution to a vial containing 4.8 mL of the substrate solution in a water bath at 40 °C with a magnetic stirrer bar stirring at 400 rpm (final concentration 50 mM substrate, 0.125 M catalyst). 25 µl Samples were taken at selected time intervals and quenched by addition to 975 µl of a L-glutathione solution (50 µl of 250 mM L-glutathione in water, 300 µl water and 625 µl MeOH). Samples were filtered through 0.22 µm nylon membranes. The samples were analyzed by HPLC.

The reaction mixtures were visually inspected at the start and end of the reaction. All of the solutions remained transparent and no precipitate was visible indicating sufficient solubility of catalysts **3a**-**3g** in solution.

HPLC ANALYSIS – NON-CHIRAL

Athena C18-WP column (100 Å, 4.6 x 25 mm, CNW); solvent A: H_20 + 0.1% TFA, solvent B: MeOH + 0.1% TFA. 10% B ramping to 50% B at 20 min, 90% B 20.5-24 min, 10% B 24.5-35 min; flow rate 1 mL min^{-1}, 35 °C. Retention times: product = 11.4 min, substrate = 12.9 min, caffeine = 15.2 min.

Conversion of substrate to product was calculated from the peak areas using the calibration curves for the substrate and product. The first order rate constants were derived from plotting ln([substrate]) against time. Data points up to 90% conversion were used to plot a line of least squares except in cases where fewer than 4 data points were in this range; in this case data points up to 95% conversion were included. Due to significantly lower activity, data points for the catalyst with the CF_3 substituent in the time range 0 to 480 s was used for the least squares fit with 480 s the first data point to exceed 50% conversion. The first order rate constants were extracted from the gradient (gradient = -k). Three repeats were carried out for each catalyst and the average rate constant calculated from the gradients of each repetition.

HPLC ANALYSIS – CHIRAL

Samples were filtered through 0.22 µm nylon membranes. The samples were analyzed by HPLC (Agilent 1260) using a Lux Cellulose-4 column (Phenomenex, 250 mm x 4.6 mm, 5µm); solvent A: H_20 + 20 mM $(NH_4)HCO_3$ adjusted to pH 8.75 using DEA, solvent B: MeCN. 10% B ramping to 13% B at 12 mins, ramping to 40% B at 12-25 mins, ramping back to 10% B 20-25 mins and maintaining 10% B 25-30 mins; flow rate 1.5 mL min^{-1}, 35 °C. Retention times (*S*)-product = 9.6 min, (*R*)-product = 10.6 min, substrate = 13.0 mins.

CALIBRATION DATA

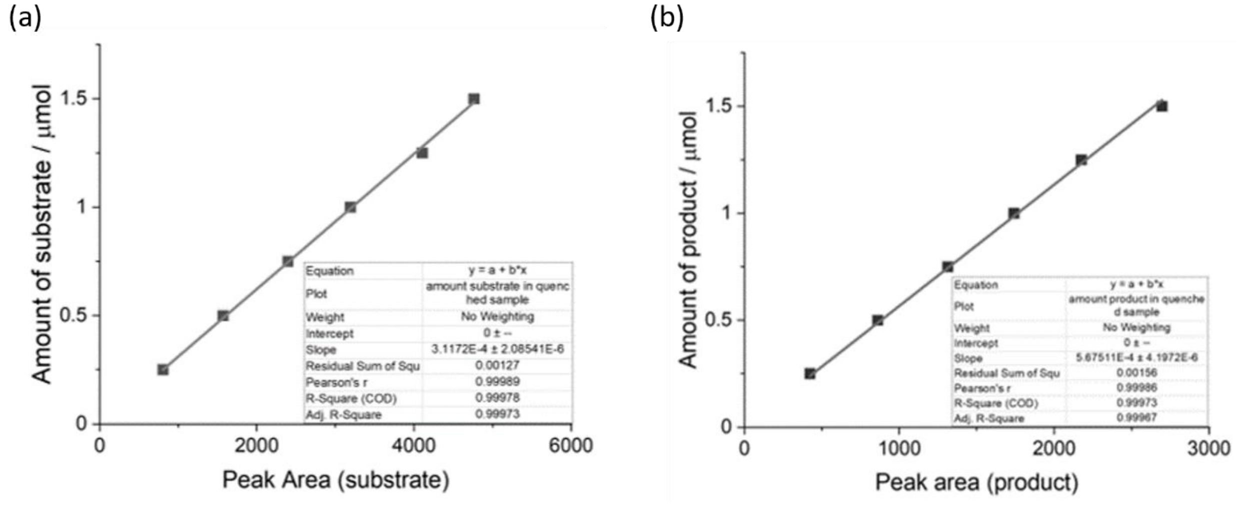

Figure S4. Calibration plots for (a) substrate and (b) product for peak area by HPLC analysis.

CATALYTIC ACTIVITY DATA

Table S2. First order rate constants for **3a**-**3g**.

**Substituent**	**First order rate constant (k)/ s^{-1}**					
	**rep 1**	**rep 2**	**rep 3**	**rep 4**	**Average**	**error**
***m*-CF_3**	0.0009	0.0011	0.0015		0.0012	0.0003
***m*-Cl**	0.0069	0.0064	0.0068		0.0067	0.0002
***m*-F**	0.0057	0.0053	0.0051		0.0054	0.0003
**H**	0.0092	0.0096	0.0092	0.0094	0.0094	0.0002
***m*-Me**	0.014	0.012	0.010		0.012	0.002
***p*-Me**	0.012	0.011	0.010		0.011	0.0009
***m*-NMe_2**	0.0083	0.0086	0.0082		0.0083	0.0002

CONTROL EXPERIMENTS

There was no conversion of 6,7-dimethoxy-1-methyl-3,4-dihydroisoquinoline to the corresponding product under reaction conditions in the absence of a catalyst.

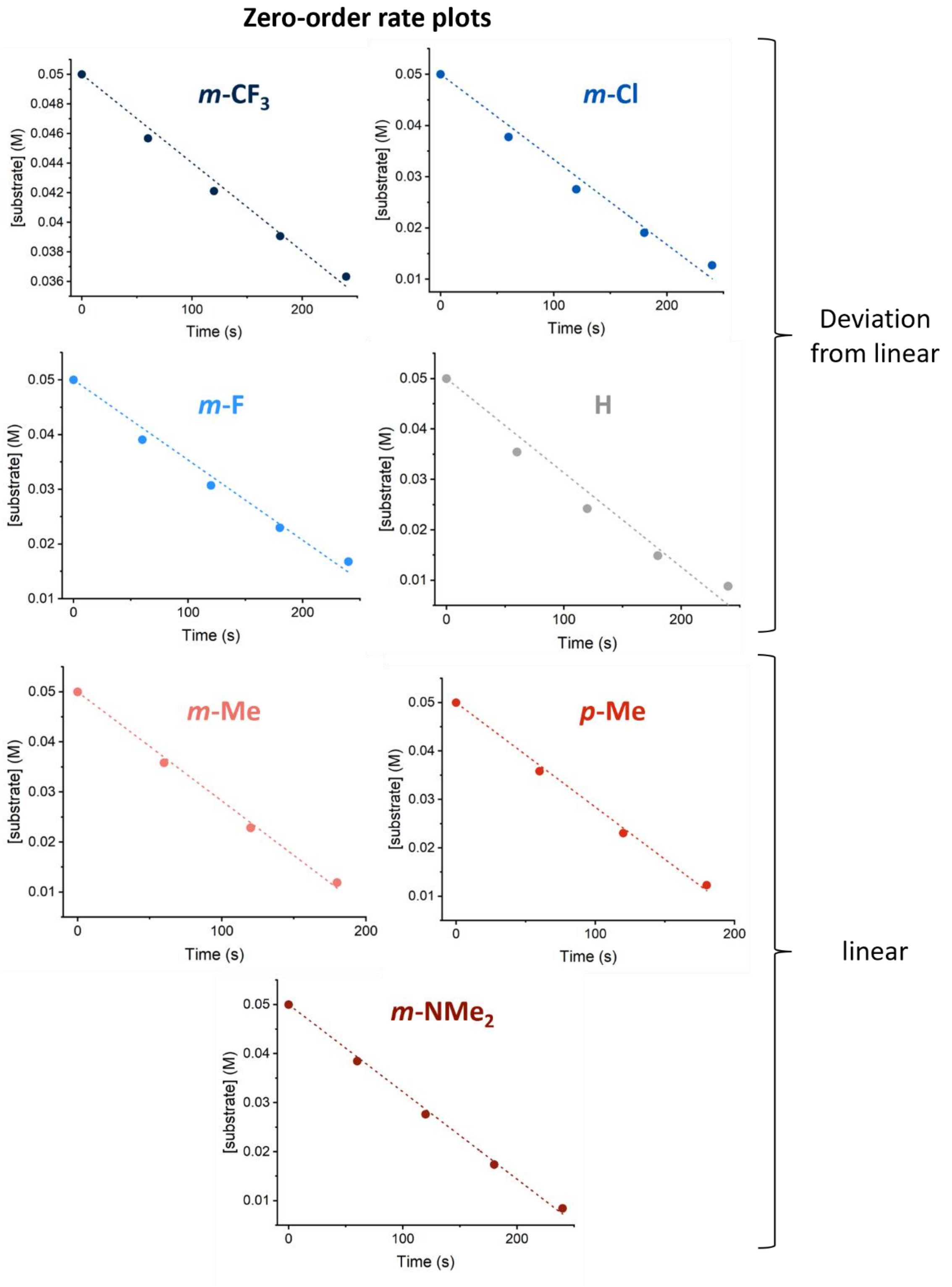

Figure S5. Plots of the concentration of substrate against time for the reaction catalyzed by **3a**-**3g**. The zero-order rate constants are derived from the gradient of the linear least squares fit, indicated by a dashed line.

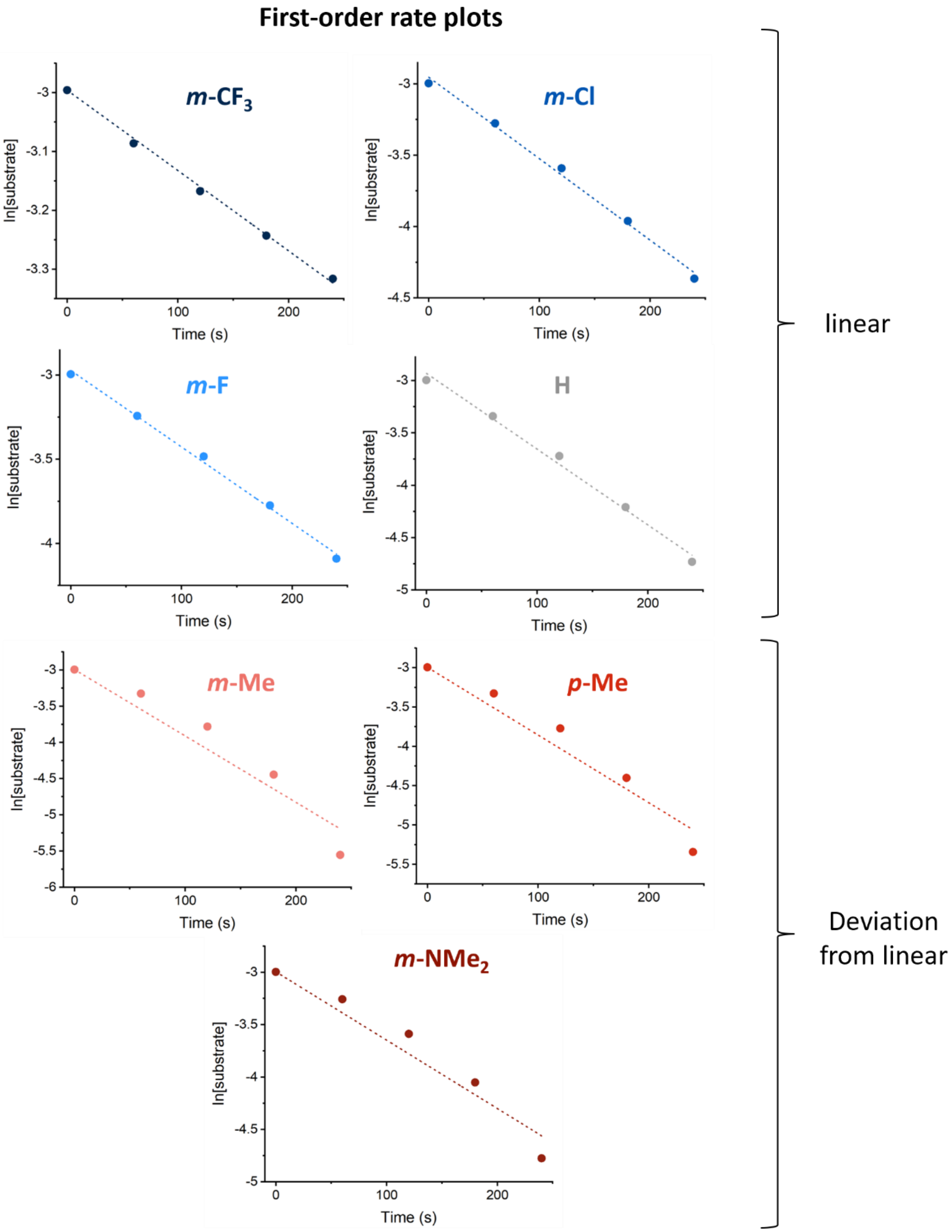

Figure S6. Plots of ln(concentration of substrate) against time for the reaction catalyzed by **3a**-**3g**. The first-order rate constants are derived from the gradient of the linear least squares fit, indicated by a dashed line.

8. ALTERNATIVE HAMMETT PLOTS

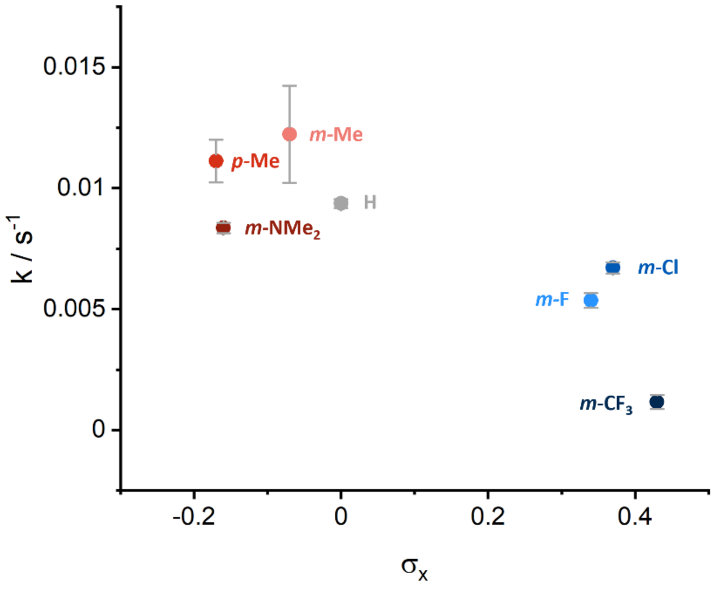

Figure S7. Plot of the first-order rate constants for **3a-3g** against Hammett parameter σ_x, where σ_x represents either σ_m or σ_p as appropriate.

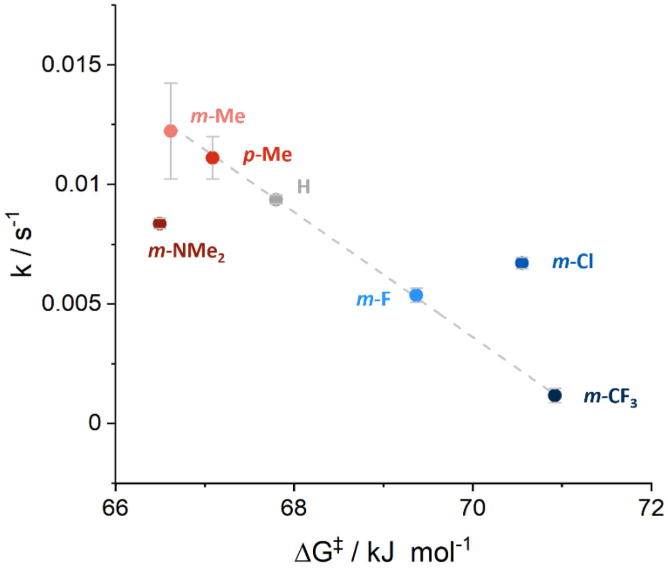

Figure S8. Plot of the first-order rate constants for **3a-3g** against $\Delta G^{\ddagger}$ calculated from variable temperature NMR experiments.

9. SPECTROSCOPIC EXPERIMENTS TO IDENTIFY THE SPECIES PRESENT IN AQUEOUS SOLUTION

EXPERIMENT A:

Complex **3d** was dissolved in a 8:2 ratio of D_2O:MeOH-d_4 and a ^1H NMR spectrum recorded (20% proportion of methanol was necessary for achieving the required concentration). An excess of silver hexafluorophosphate was added, the sample mixed vigorously and left to stand for 30 mins. A second ^1H NMR spectra was then recorded of the supernatant. Some small, but distinctive, changes were observed between the spectra, notably a downfield shift of the signals corresponding to the protons of the pyridine ring (inset, **Figure S10**).

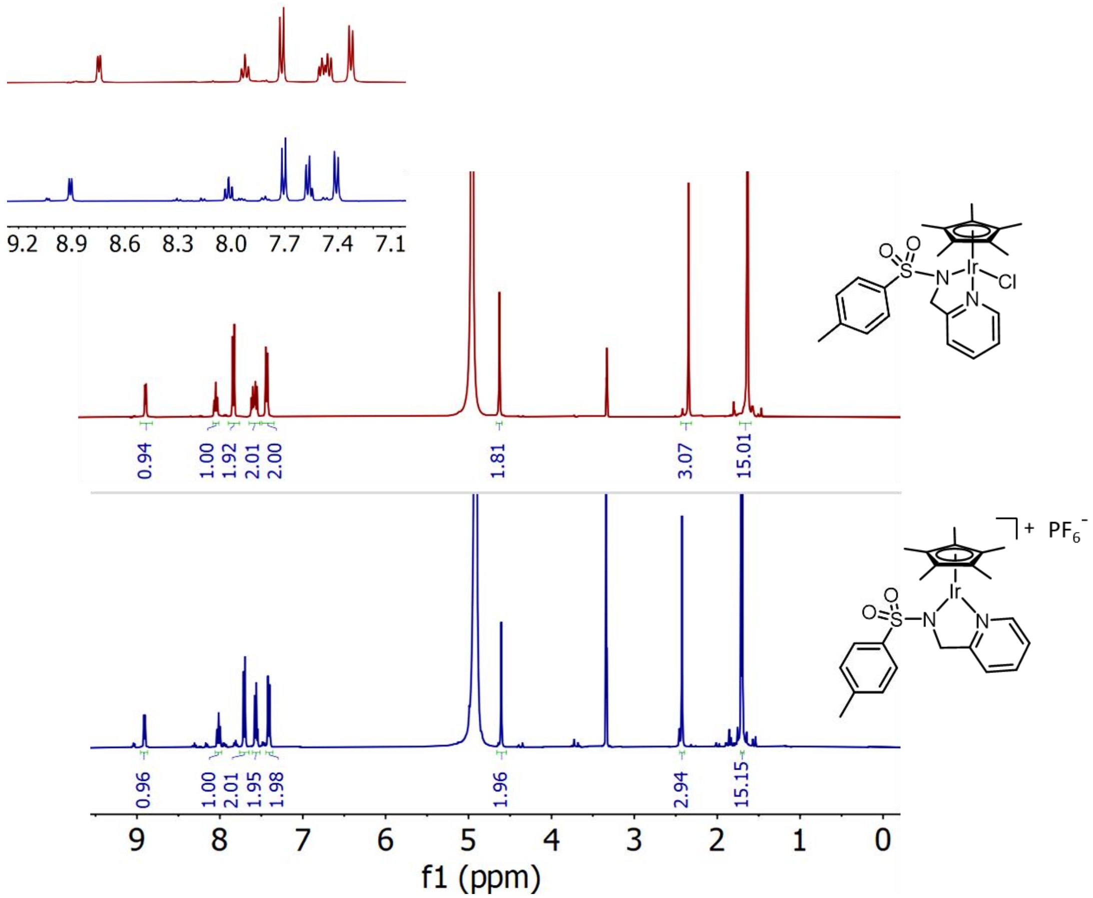

Figure S9. ^1H NMR spectra of complex **3d** in 8:2 D_2O:MeOH-d_4 before (red spectrum; top) and after (blue spectrum; bottom) addition of silver hexafluorophosphate. The inset shows resonances in the aromatic region where downfield shifts in the resonances corresponding to protons of the pyridine ring are observed.

EXPERIMENT B:

Complex **3d** was dissolved in a 8:2 ratio of D_2O:MeOH-d_4 and a ^1H NMR spectrum recorded. Increasing amounts of NaCl were then added to the sample and ^1H NMR spectra recorded for 10 mM NaCl, 50 mM NaCl and saturated NaCl solutions. All spectra show only one set of signals and the small shifts observed in the resonances are consistent with the gradual increase in ionic strength.

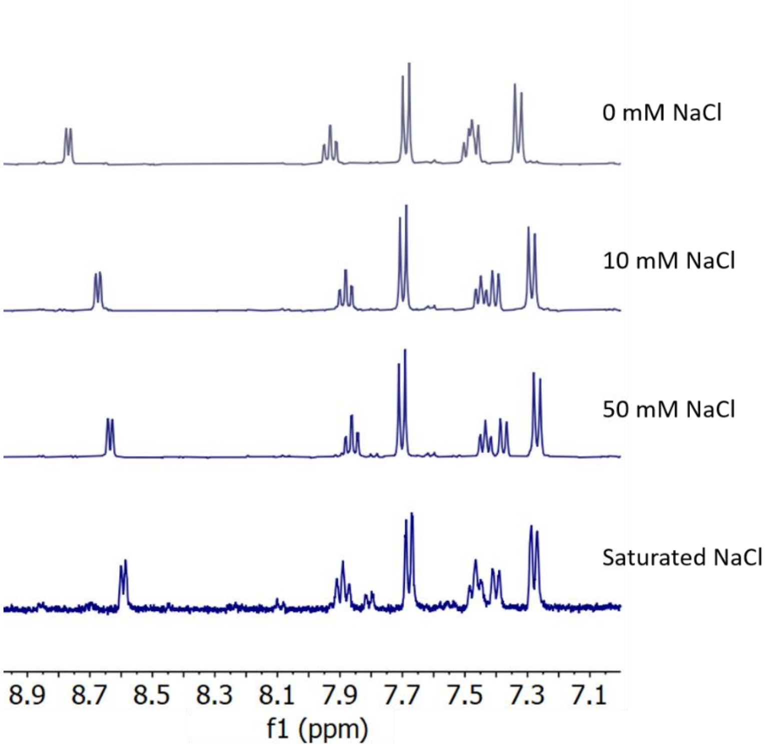

Figure S10. ^1H NMR spectra of complex **3d** between 7.1-8.9 ppm at increasing concentrations of NaCl.

10. X-RAY CRYSTALLOGRAPHY

Crystals were obtained from the final solutions for Experiment A (complex **3d** in 8:2 D_2O:MeOH-d_4 following addition of $AgPF_6$; **Figure S11**, left) and Experiment B (complex **3d** in 8:2 D_2O:MeOH-d_4 saturated with NaCl; **Figure S11**, right) described above.

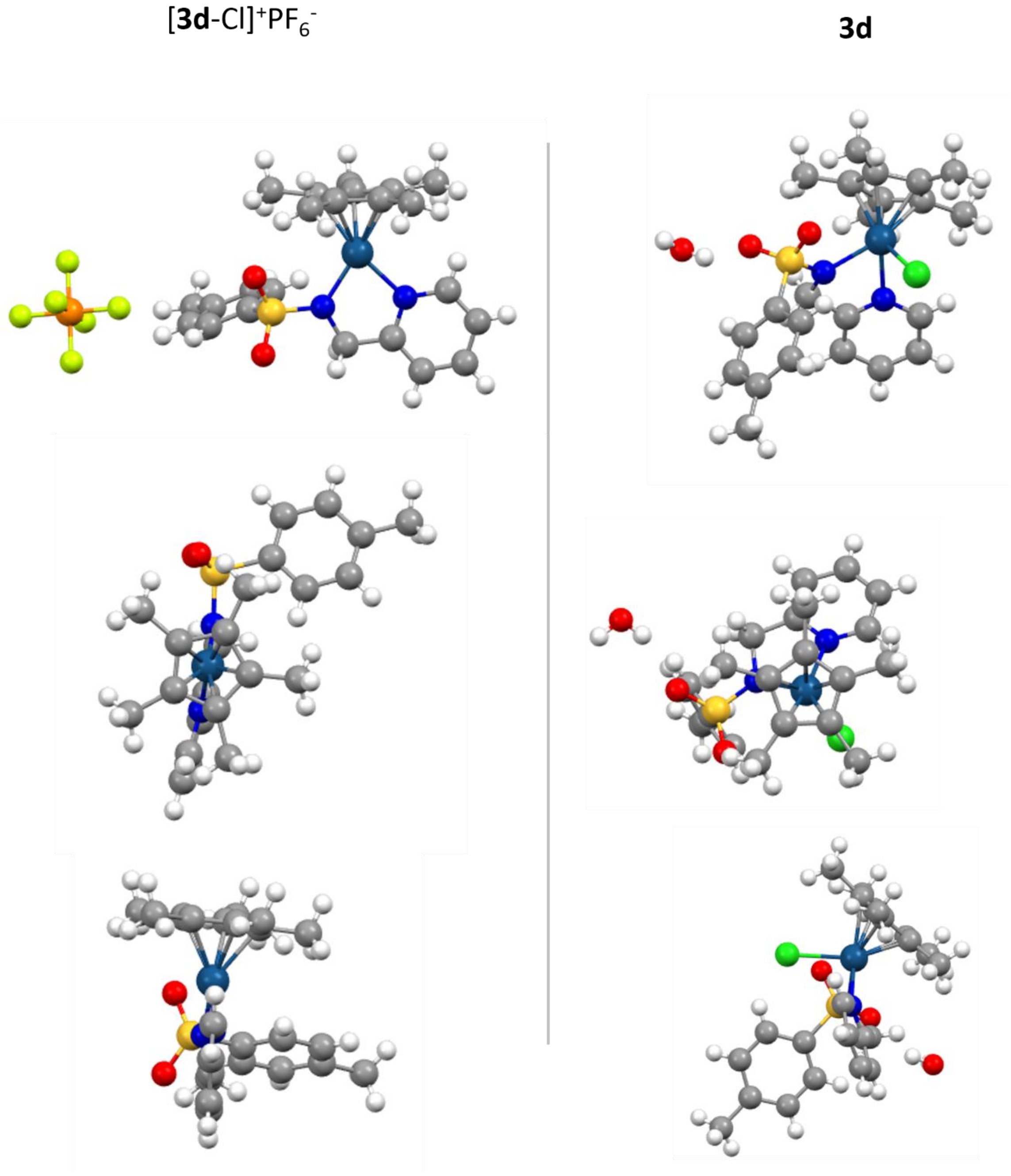

Figure S11. X-ray crystal structures of the crystals derived from Experiment A ([**3d**-Cl]$^+PF_6^-$;left) and Experiment B (**3d** from saturated NaCl; right)

Experiment A: [**3d**-Cl]$^+$$PF_6^-$

Data collected, solved and refined by Dr. Adrian C. Whitwood

Refinement Special Details: The PF_6^- anion was disordered and modelled in two positions with refined occupancies of 0.619:0.381(16). Pairs of P-F bonds were restrained to be equal (e.g. P1A-F1A and P1B-F1B) as were equivalent 1-3 F-F bond distances (e.g. F1A...F2A and F1B...F2B). The ADPs of several pairs of disordered atoms were constrained to be equal (P1A & P1B, F2A & F2B, F4A & F4B, F5A & F5B, F6A & F6B).

CSD Deposition Number	2321077-2321077
Empirical formula	$C_{23}H_{28}F_6IrN_2O_2PS$
Formula weight	733.70
Temperature/K	110.00(10)
Crystal system	monoclinic
Space group	$P2_1/n$
a/Å	10.2664(18)
b/Å	13.878(2)
c/Å	18.119(3)
α/°	90
β/°	101.388(16)
γ/°	90
Volume/Å^3	2530.7(7)
Z	4
ρ_{calc}g/cm^3	1.926
μ/mm^{-1}	12.215
F(000)	1432.0
Crystal size/mm^3	0.17 × 0.12 × 0.01
Radiation	Cu Kα (λ = 1.54184)
2Θ range for data collection/°	9.204 to 134.13
Index ranges	$-8 \leq h \leq 12$, $-15 \leq k \leq 16$, $-21 \leq l \leq 21$
Reflections collected	13826
Independent reflections	4480 [R_{int} = 0.0551, R_{sigma} = 0.0458]
Data/restraints/parameters	4480/27/347
Goodness-of-fit on F^2	1.047
Final R indexes [I>=2σ (I)]	R_1 = 0.0636, wR_2 = 0.1501
Final R indexes [all data]	R_1 = 0.0730, wR_2 = 0.1561
Largest diff. peak/hole / e Å^{-3}	3.84/-4.17

Experiment B: **3d**

Data collected, solved and refined by Dr. Adrian C. Whitwood

Refinement Special details: Non-merohedral twin modelled with two components 0.6478:0.3522(10).

CSD Deposition Number	2321077-2321078
Empirical formula	$C_{46}H_{58}Cl_2Ir_2N_4O_5S_2$
Formula weight	1266.38
Temperature/K	109.95(10)
Crystal system	monoclinic
Space group	$P2_1$
a/Å	13.3163(2)
b/Å	8.82242(15)
c/Å	19.8323(4)
α/°	90
β/°	96.5342(16)
γ/°	90
Volume/Å^3	2314.79(7)
Z	2
ρ_{calc}g/cm^3	1.817
μ/mm^{-1}	13.272
F(000)	1244.0
Crystal size/mm^3	0.168 × 0.022 × 0.011
Radiation	Cu Kα (λ = 1.54184)
2Θ range for data collection/°	7.614 to 134.136
Index ranges	$-15 \leq h \leq 15$, $-10 \leq k \leq 10$, $-21 \leq l \leq 23$
Reflections collected	11077
Independent reflections	11077 [R_{int} = ?, R_{sigma} = 0.0391]
Data/restraints/parameters	11077/37/566
Goodness-of-fit on F^2	1.053
Final R indexes [I>=2σ (I)]	R_1 = 0.0429, wR_2 = 0.1037
Final R indexes [all data]	R_1 = 0.0452, wR_2 = 0.1050
Largest diff. peak/hole / e Å^{-3}	1.46/-0.94
Flack parameter	-0.022(10)

11. ^{1}H AND ^{13}C NMR SPECTRA

Ligand **a**

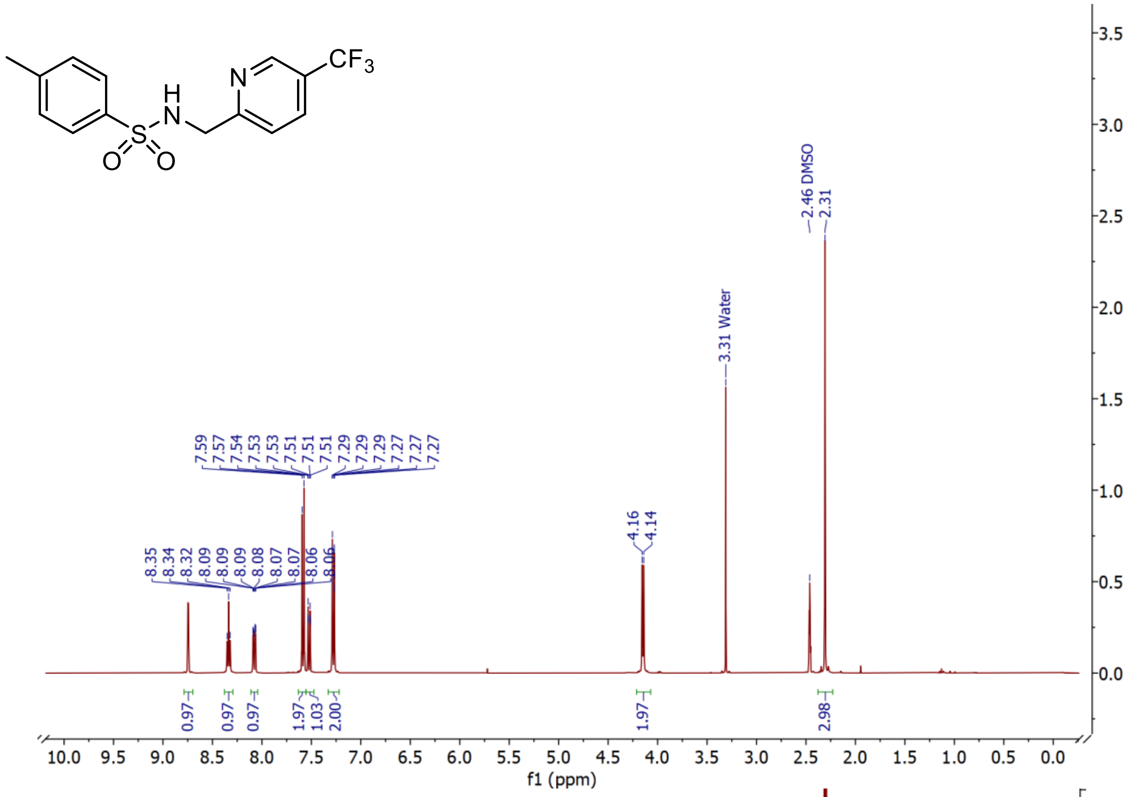

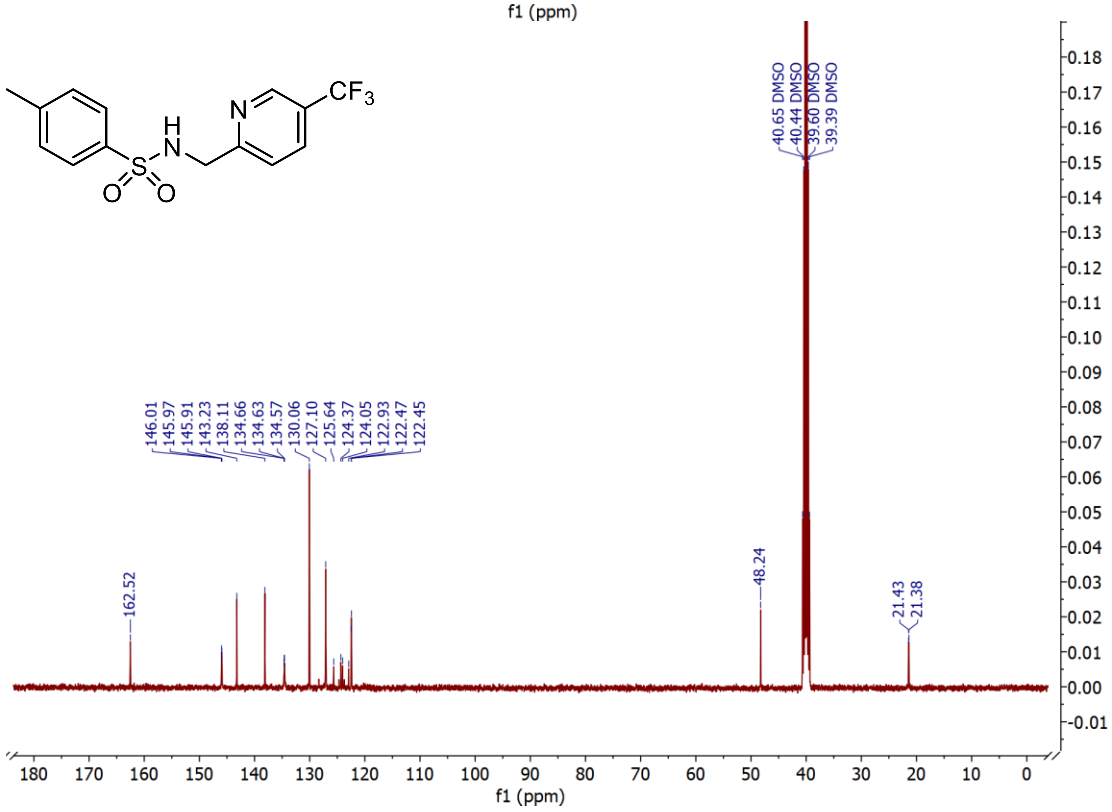

Ligand **b**

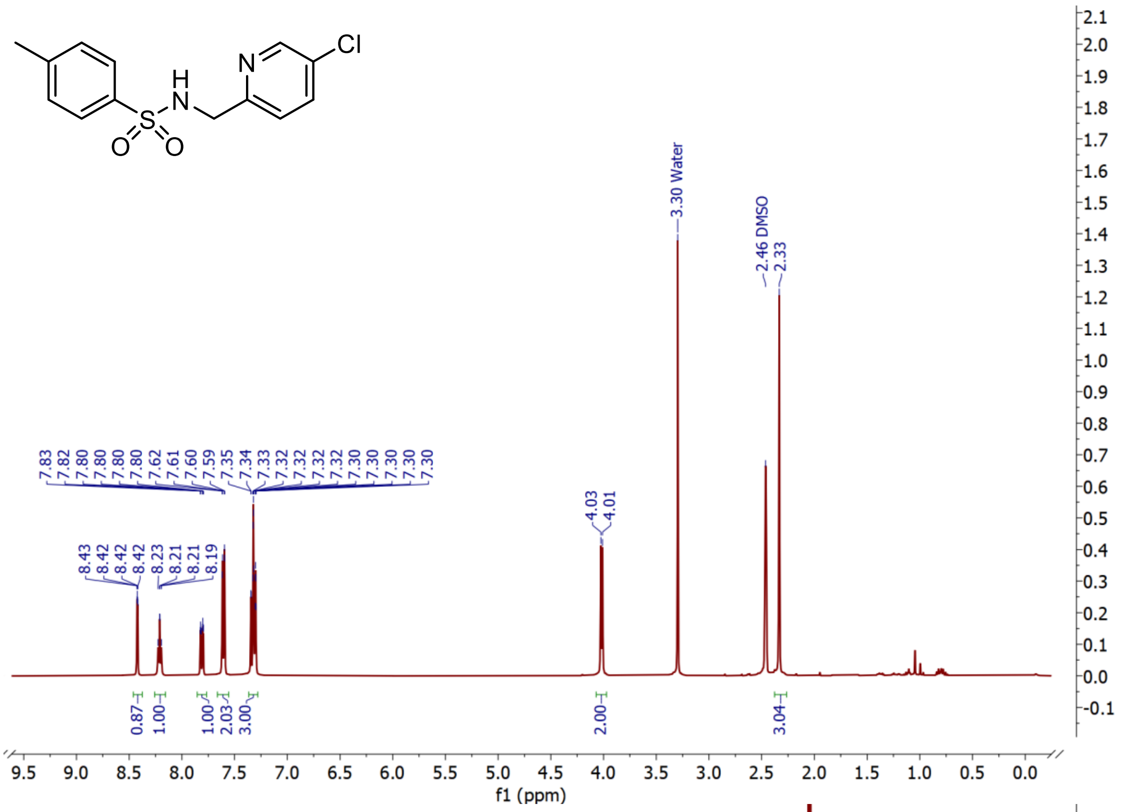
Cl
N
H
N
S
O
O
3.30 Water
2.46 DMSO
2.33
4.03
4.01
8.43 8.42 8.42 8.42 8.23 8.21 8.21 8.19
7.83 7.82 7.80 7.80 7.80 7.80 7.62 7.61 7.60 7.59 7.35 7.34 7.33 7.32 7.32 7.32 7.32 7.30 7.30 7.30 7.30 7.30
0.87
1.00
1.00
2.03
3.00
2.00
3.04
f1 (ppm)

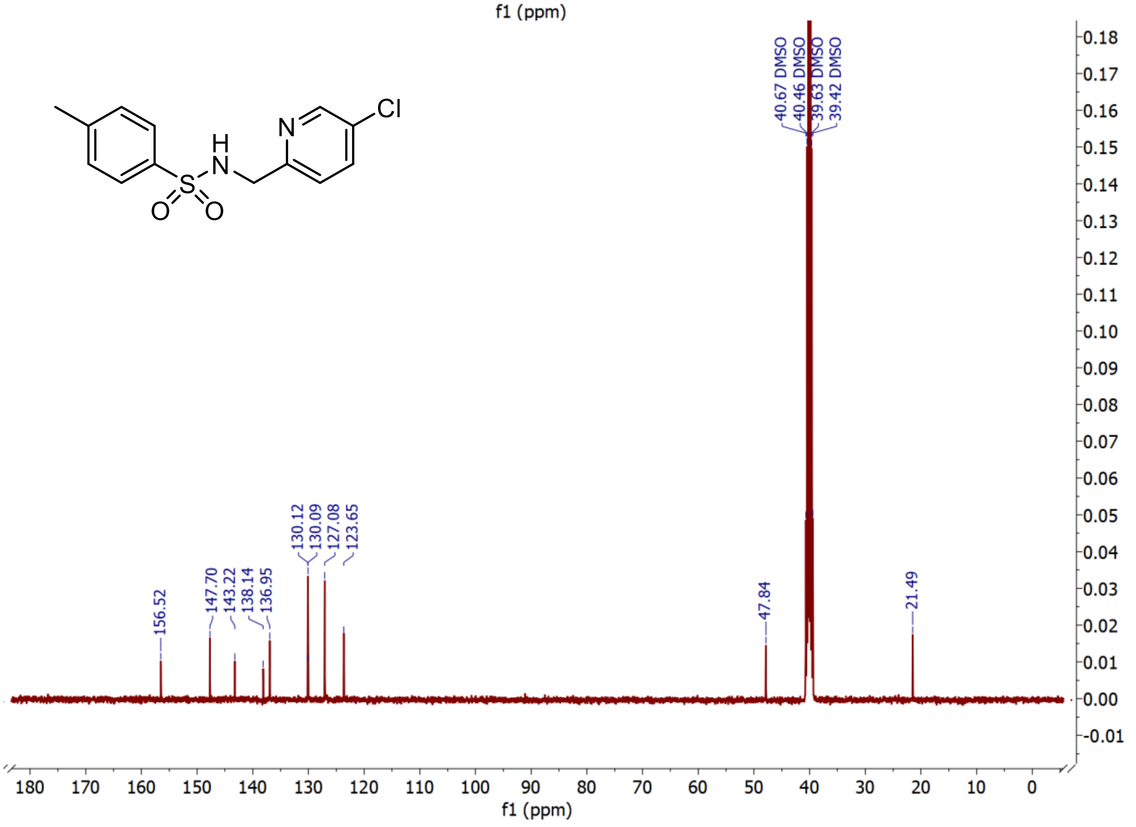
Cl
N
H
N
S
O
O
40.67 DMSO
40.46 DMSO
39.63 DMSO
39.42 DMSO
156.52
147.70
143.22
138.14
136.95
130.12
130.09
127.08
123.65
47.84
21.49
f1 (ppm)

Ligand **c**

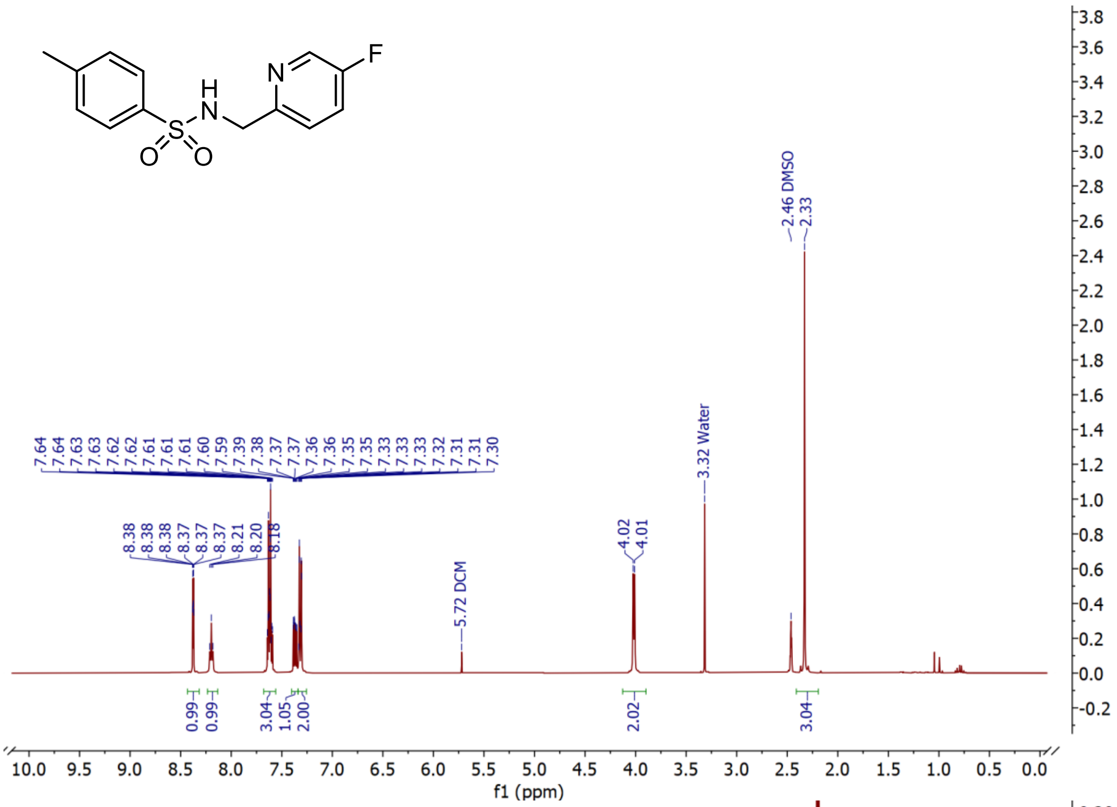

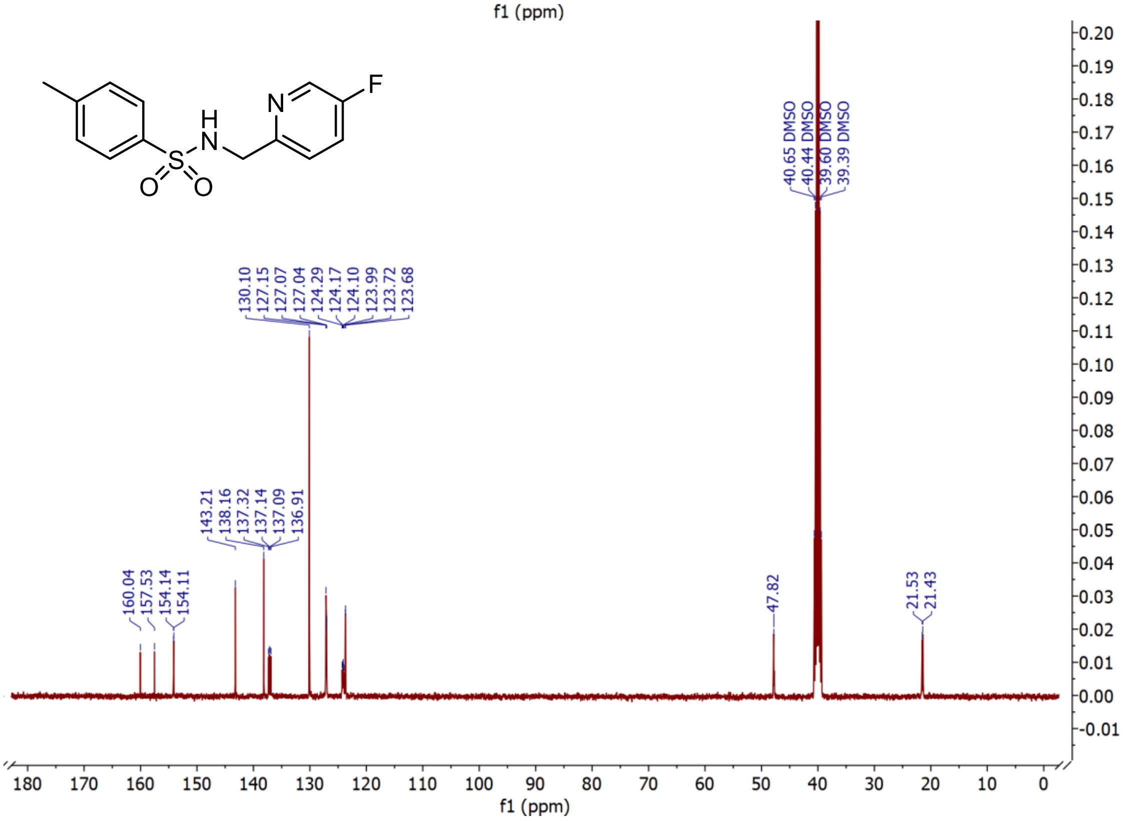

Ligand **d**

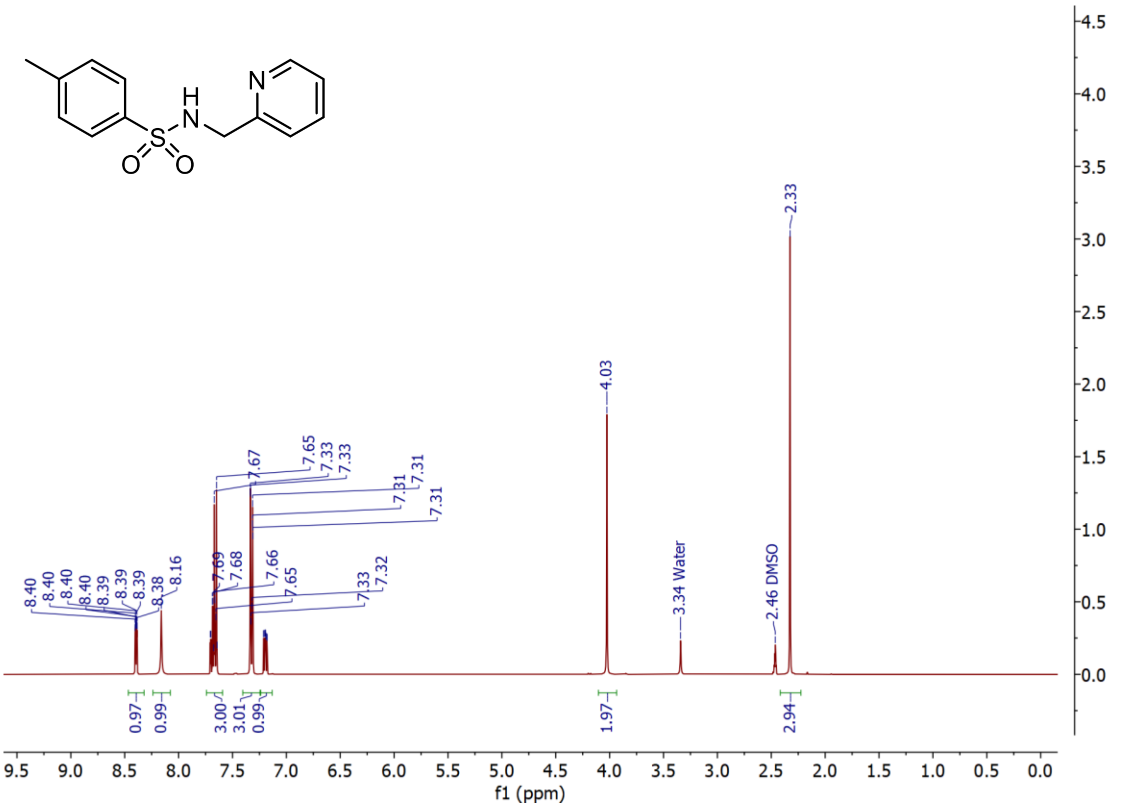

8.40
8.40
8.40
8.40
8.39
8.39
8.39
8.38
8.16
7.69
7.68
7.67
7.66
7.65
7.65
7.33
7.33
7.33
7.32
7.31
7.31
7.31
4.03
3.34 Water
2.46 DMSO
2.33
0.97
0.99
3.00
3.01
0.99
1.97
2.94
f1 (ppm)

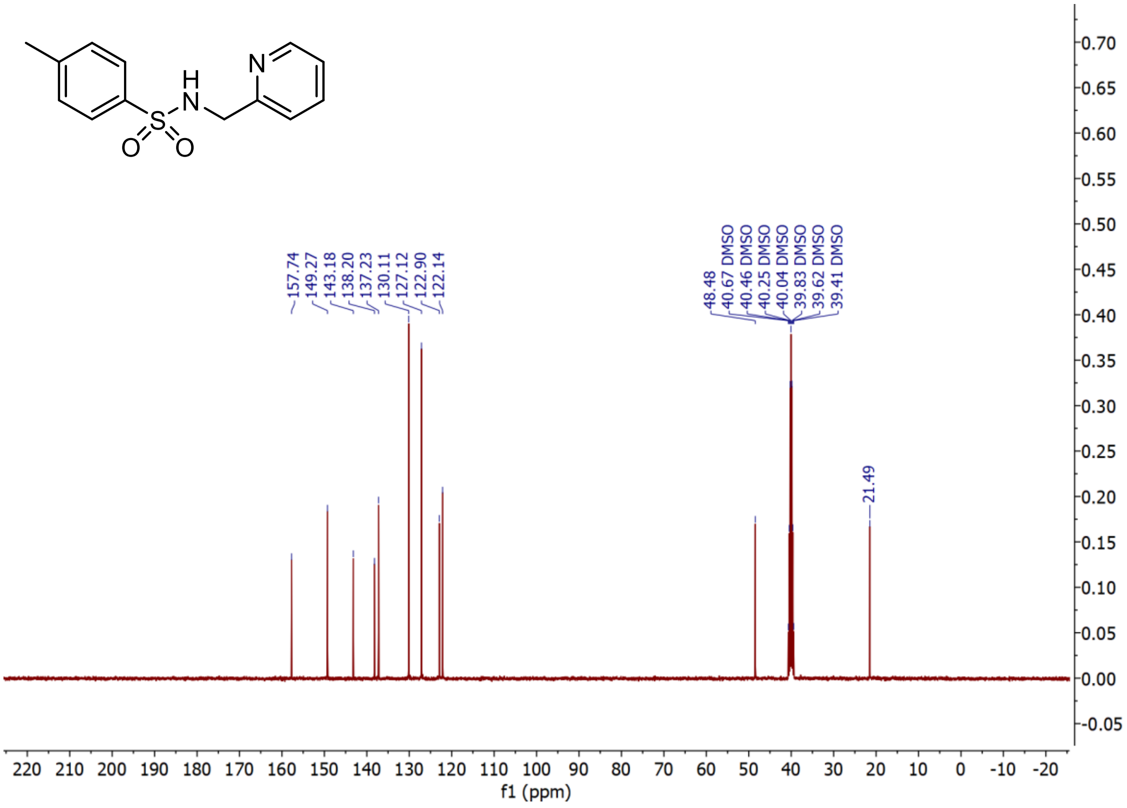

157.74
149.27
143.18
138.20
137.23
130.11
127.12
122.90
122.14
48.48
40.67 DMSO
40.46 DMSO
40.25 DMSO
40.04 DMSO
39.83 DMSO
39.62 DMSO
39.41 DMSO
21.49
f1 (ppm)

Ligand **e**

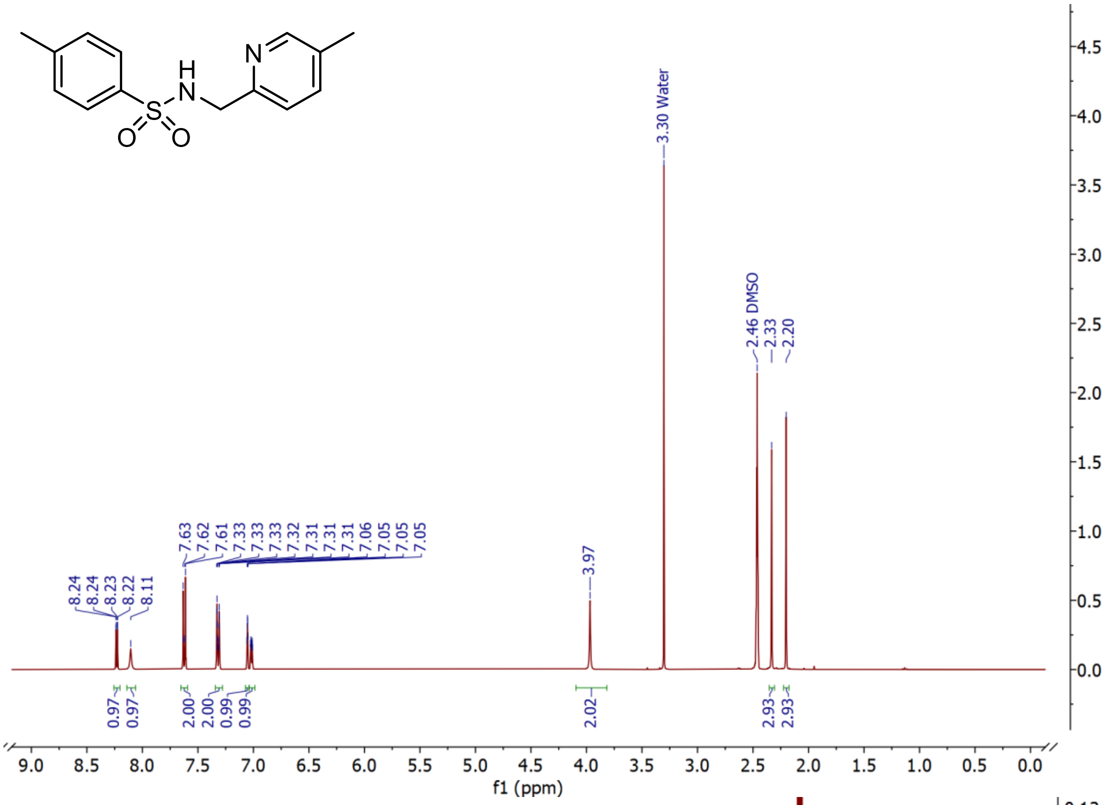

8.24
8.24
8.23
8.22
8.11
7.63
7.62
7.61
7.33
7.33
7.33
7.32
7.31
7.31
7.31
7.06
7.05
7.05
7.05
3.97
3.30 Water
2.46 DMSO
2.33
2.20
0.97
0.97
2.00
2.00
0.99
0.99
2.02
2.93
2.93
f1 (ppm)

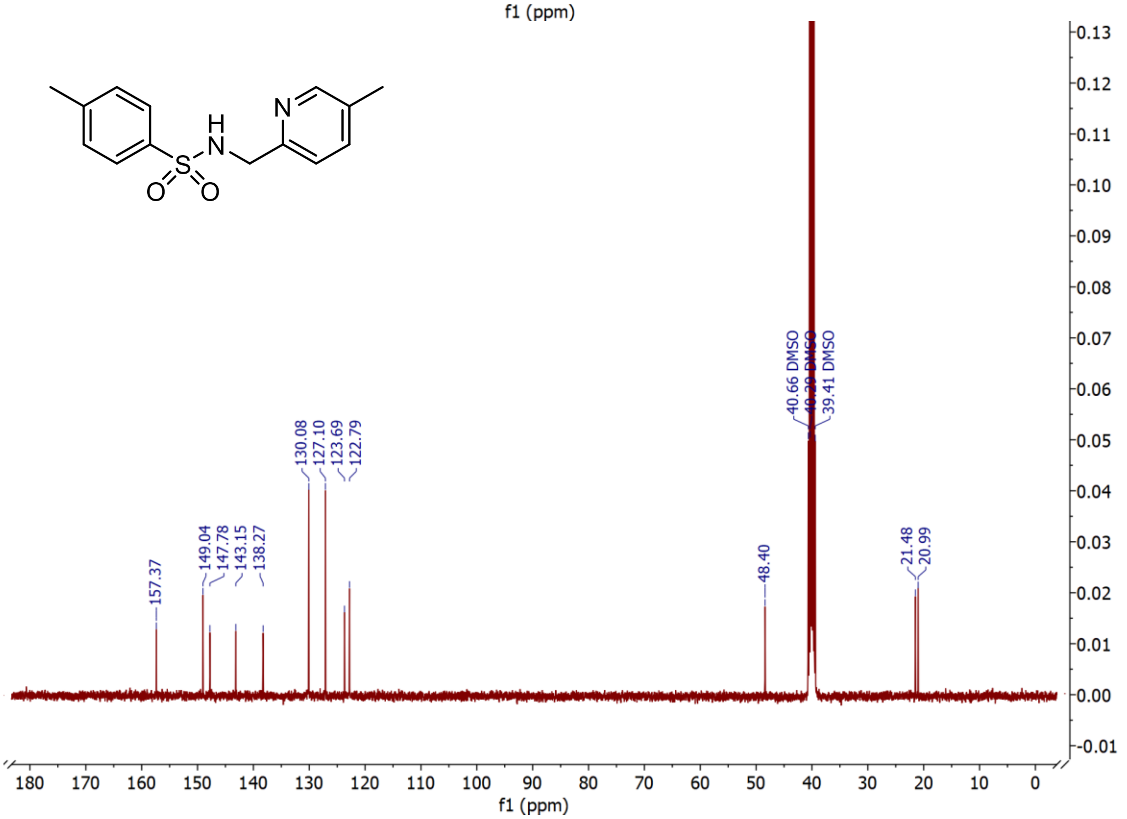

157.37
149.04
147.78
143.15
138.27
130.08
127.10
123.69
122.79
48.40
40.66 DMSO
39.41 DMSO
21.48
20.99
f1 (ppm)

Ligand **f**

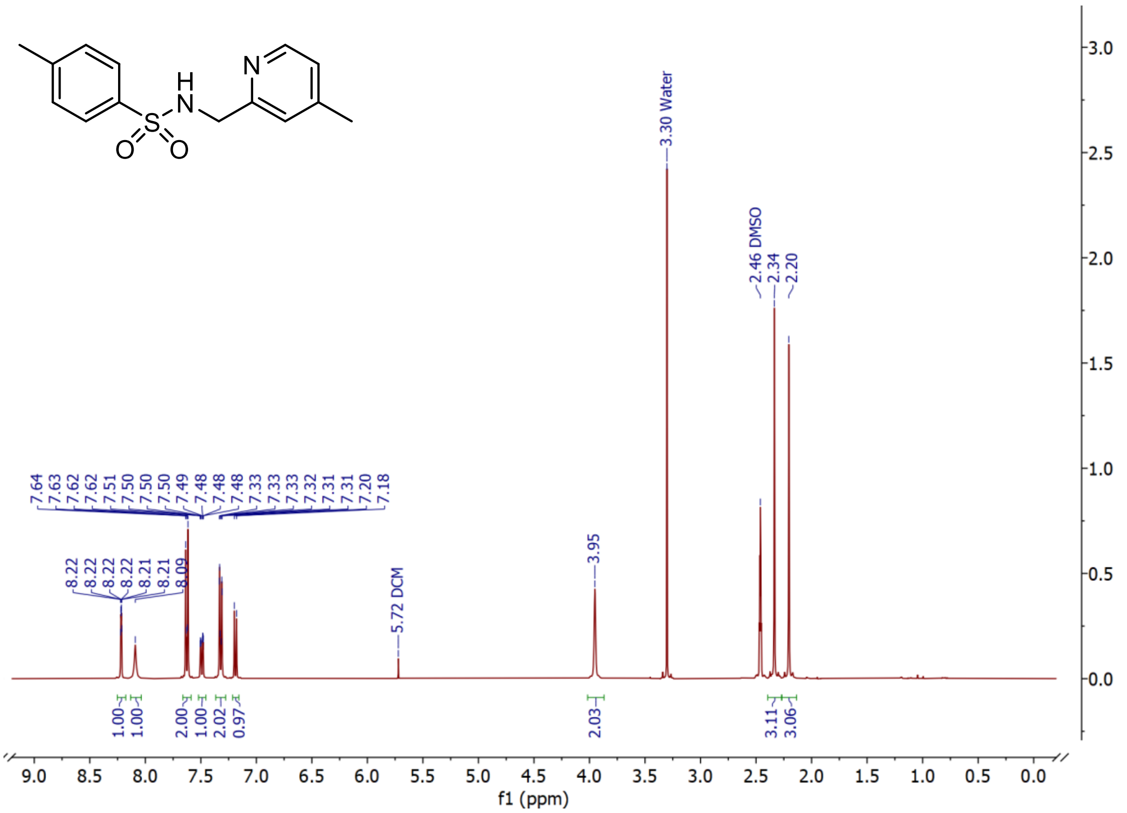

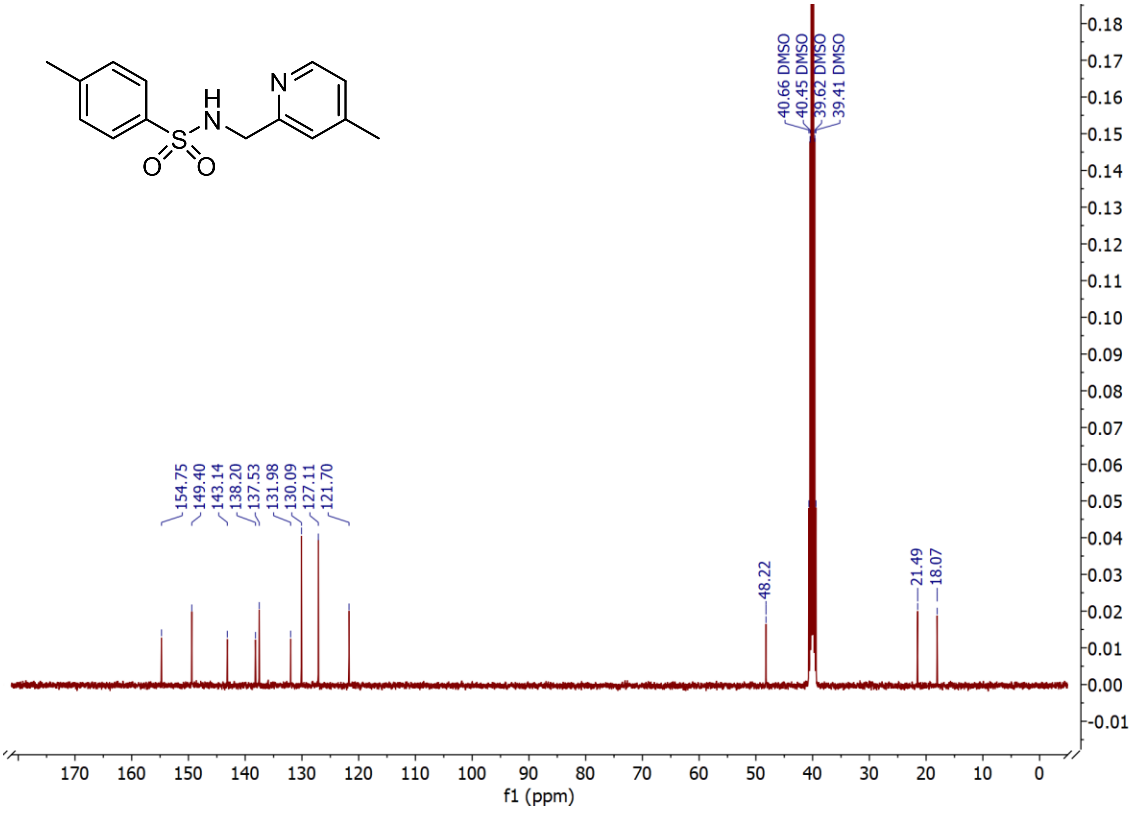

Ligand **g**

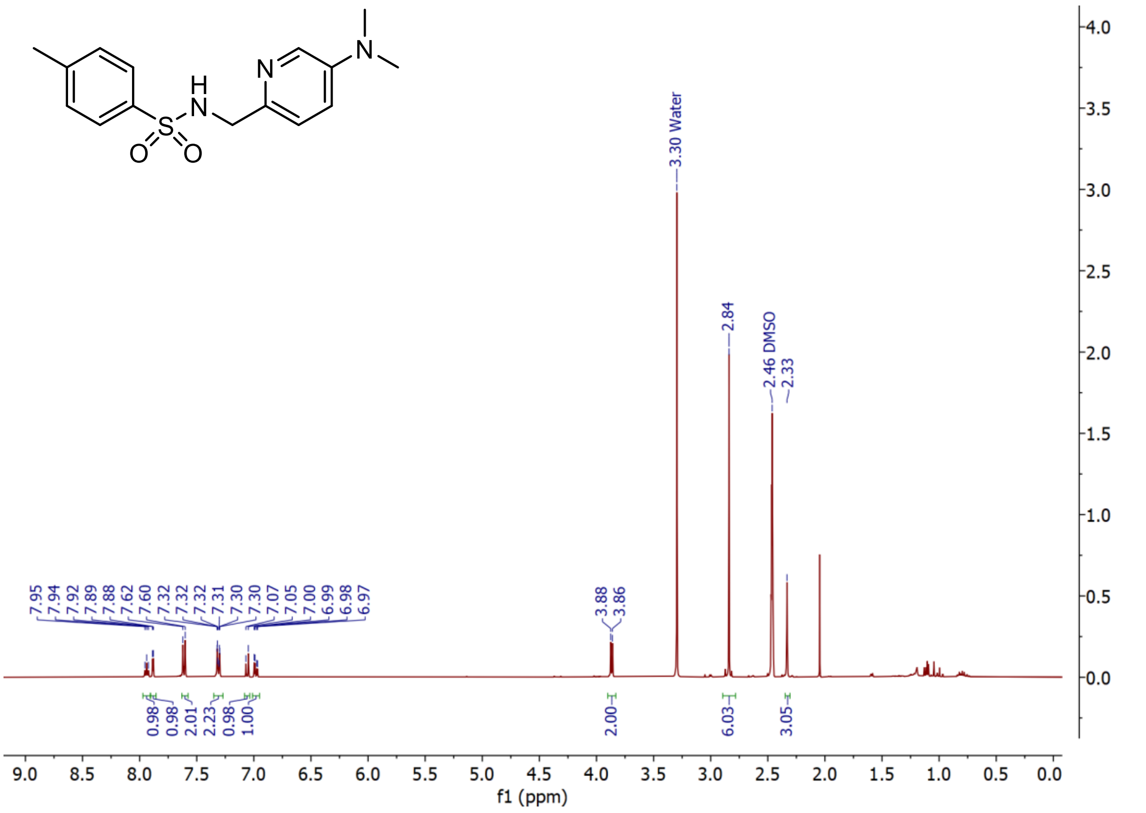

H
N
O
S
O
N
N
7.95
7.94
7.92
7.89
7.88
7.62
7.60
7.32
7.32
7.32
7.31
7.30
7.30
7.07
7.05
7.00
6.99
6.98
6.97
3.88
3.86
3.30 Water
2.84
2.46 DMSO
2.33
0.98
0.98
2.01
2.23
0.98
1.00
2.00
6.03
3.05
f1 (ppm)

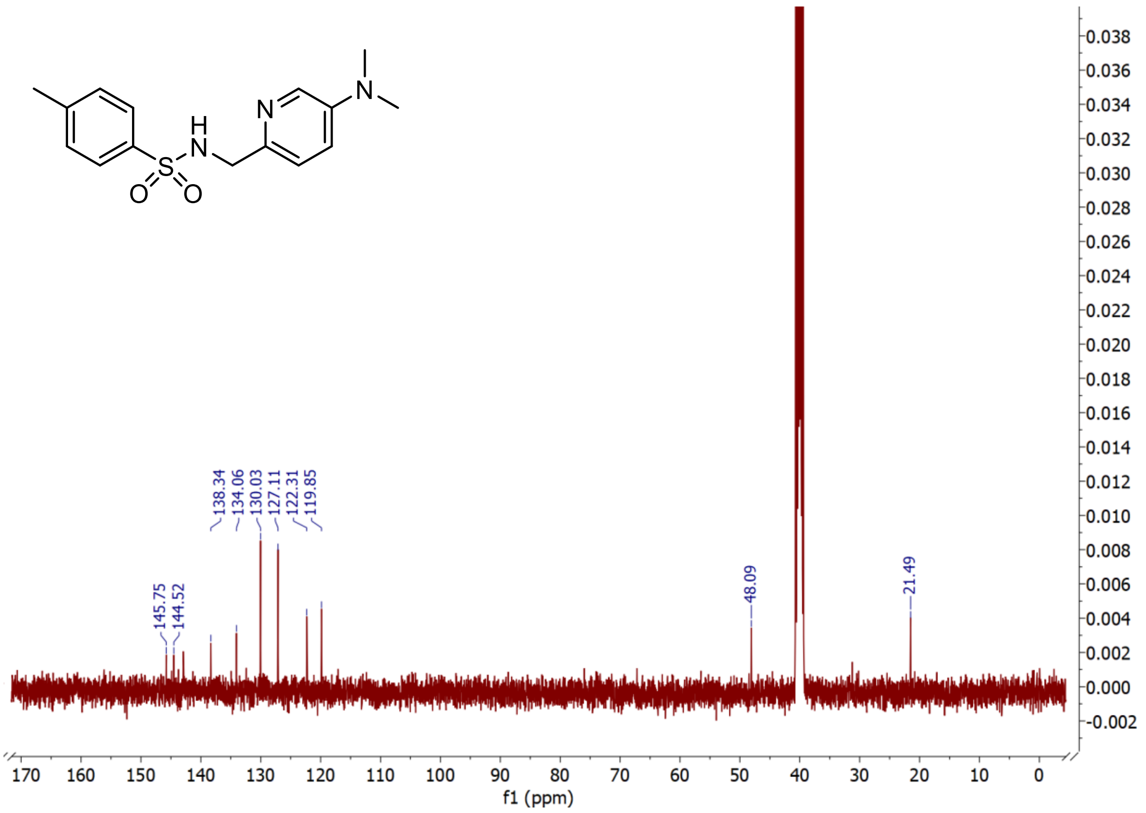

H
N
O
S
O
N
N
145.75
144.52
138.34
134.06
130.03
127.11
122.31
119.85
48.09
21.49
f1 (ppm)

[Cp*IrCl$_2$]$_2$

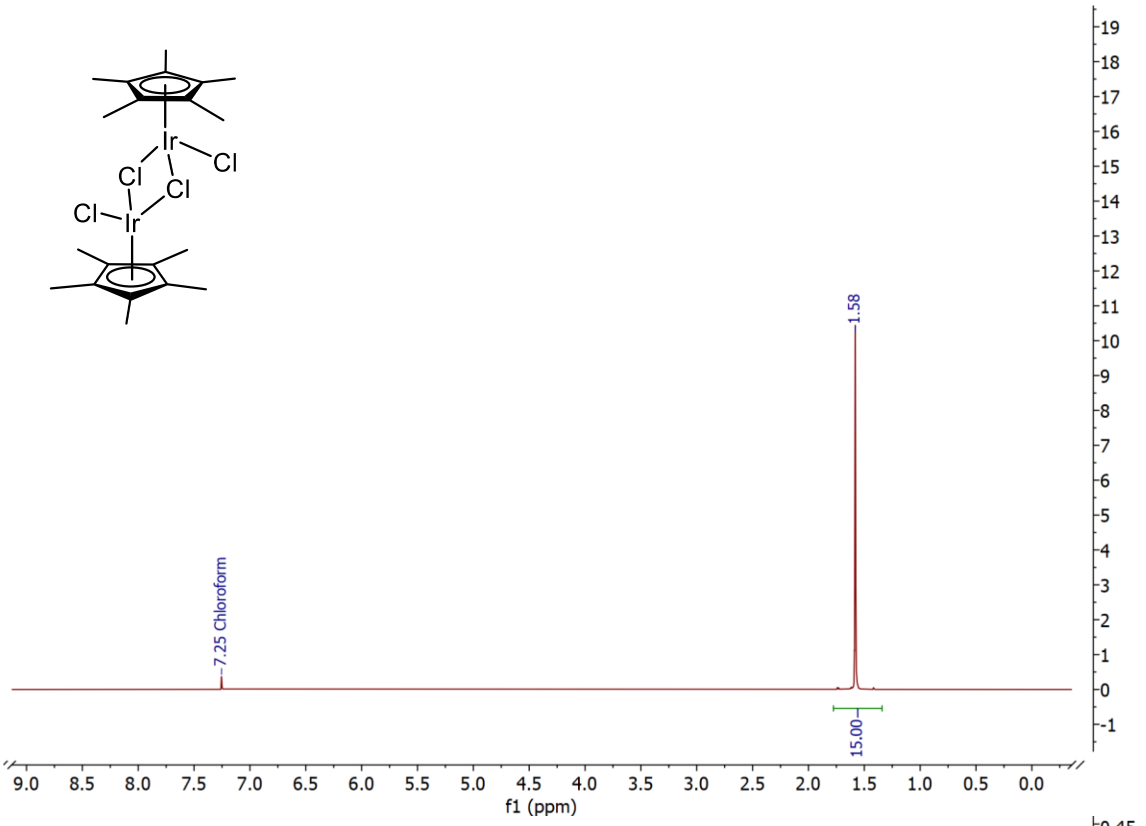
Cl
Ir
Cl
Cl
Cl
Ir
7.25 Chloroform
1.58
15.00
9.0 8.5 8.0 7.5 7.0 6.5 6.0 5.5 5.0 4.5 4.0 3.5 3.0 2.5 2.0 1.5 1.0 0.5 0.0
f1 (ppm)
19 18 17 16 15 14 13 12 11 10 9 8 7 6 5 4 3 2 1 0 -1

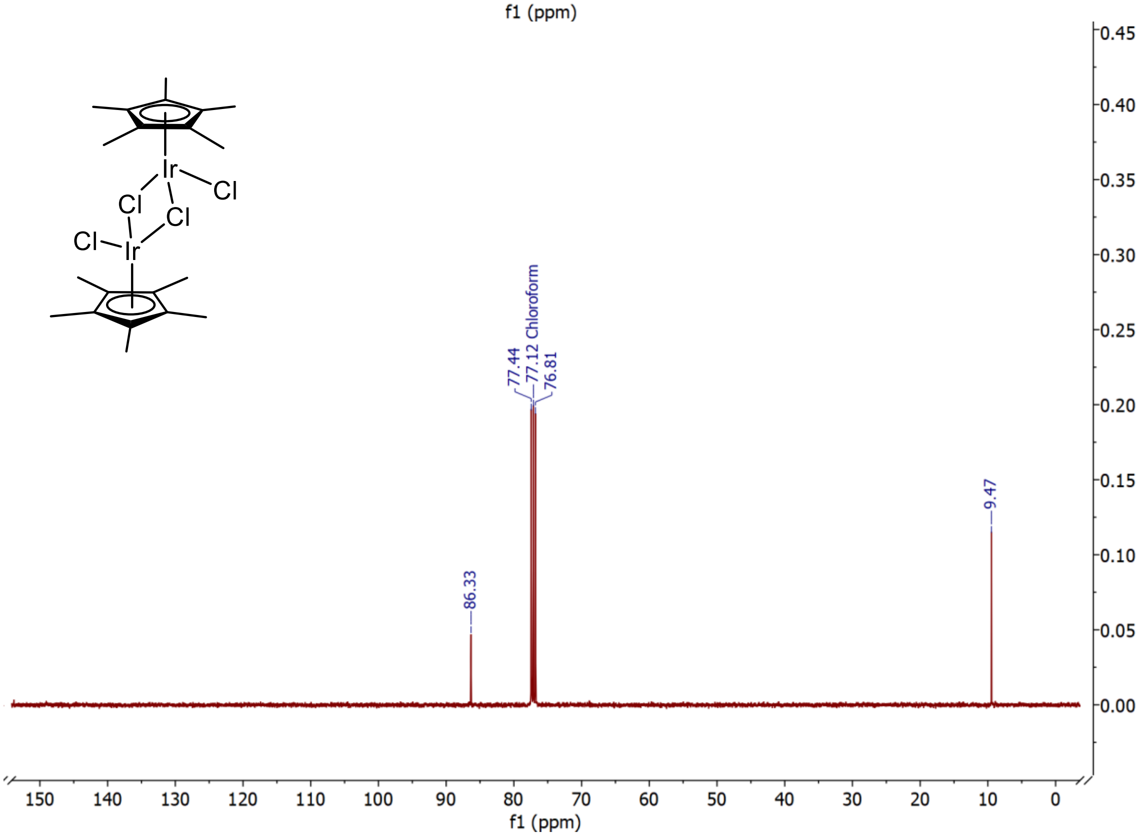
Cl
Ir
Cl
Cl
Cl
Ir
77.44
77.12 Chloroform
76.81
86.33
9.47
150 140 130 120 110 100 90 80 70 60 50 40 30 20 10 0
f1 (ppm)
0.45 0.40 0.35 0.30 0.25 0.20 0.15 0.10 0.05 0.00

Complex **3a**

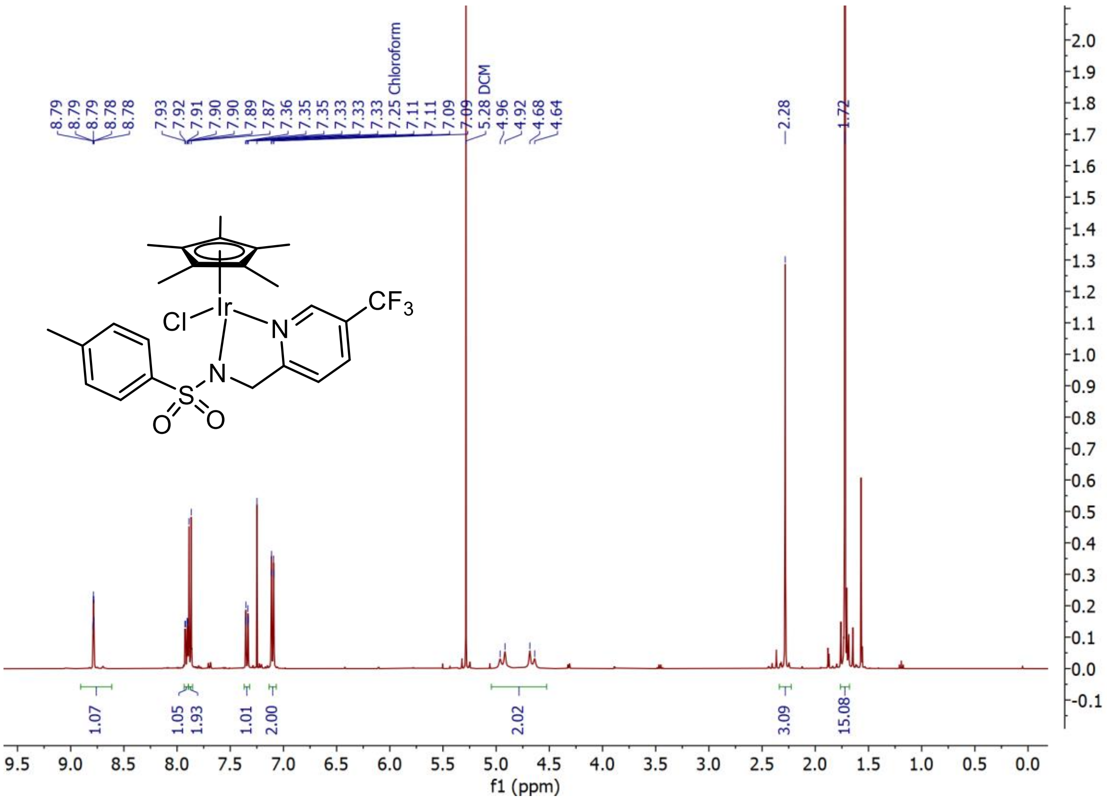

8.79
8.79
8.79
8.78
8.78
7.93
7.92
7.91
7.90
7.90
7.89
7.87
7.36
7.35
7.35
7.33
7.33
7.33
7.25 Chloroform
7.11
7.11
7.09
7.09
5.28 DCM
4.96
4.92
4.68
4.64
2.28
1.72
CF3
Cl
Ir
N
N
S
O
O
1.07
1.05
1.93
1.01
2.00
2.02
3.09
15.08
f1 (ppm)

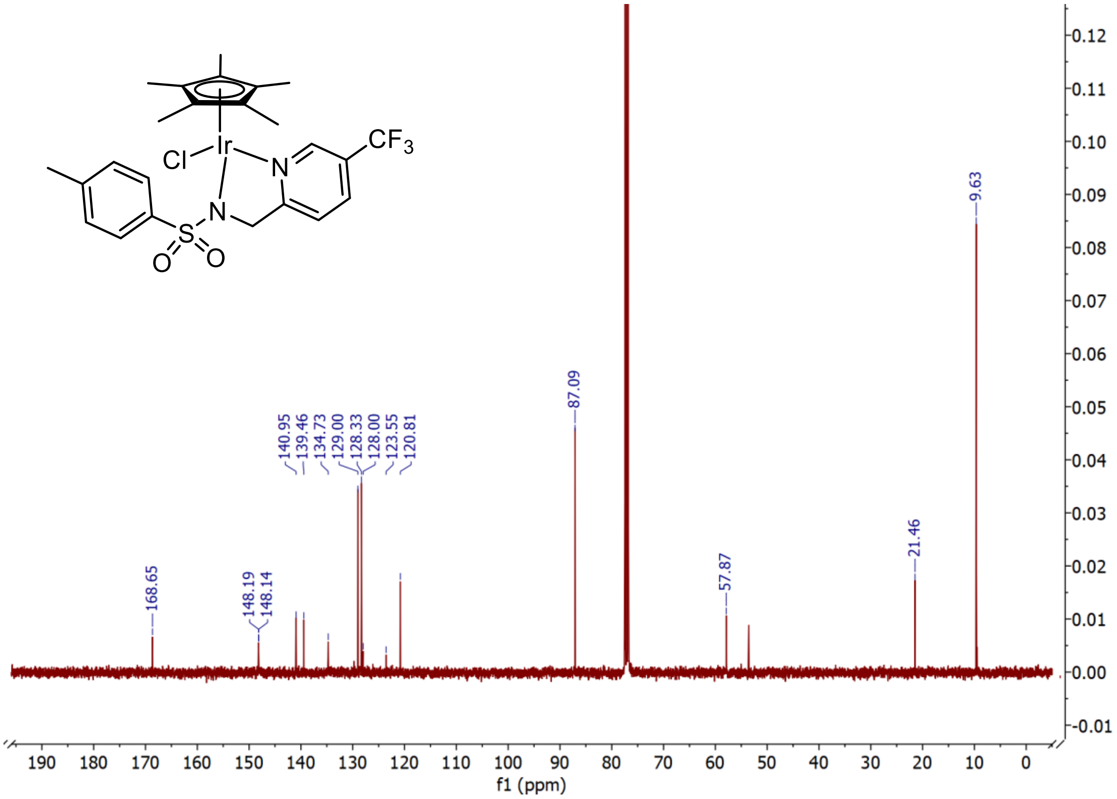

168.65
148.19
148.14
140.95
139.46
134.73
129.00
128.33
128.00
123.55
120.81
87.09
57.87
21.46
9.63
CF3
Cl
Ir
N
N
S
O
O
f1 (ppm)

Complex **3b**

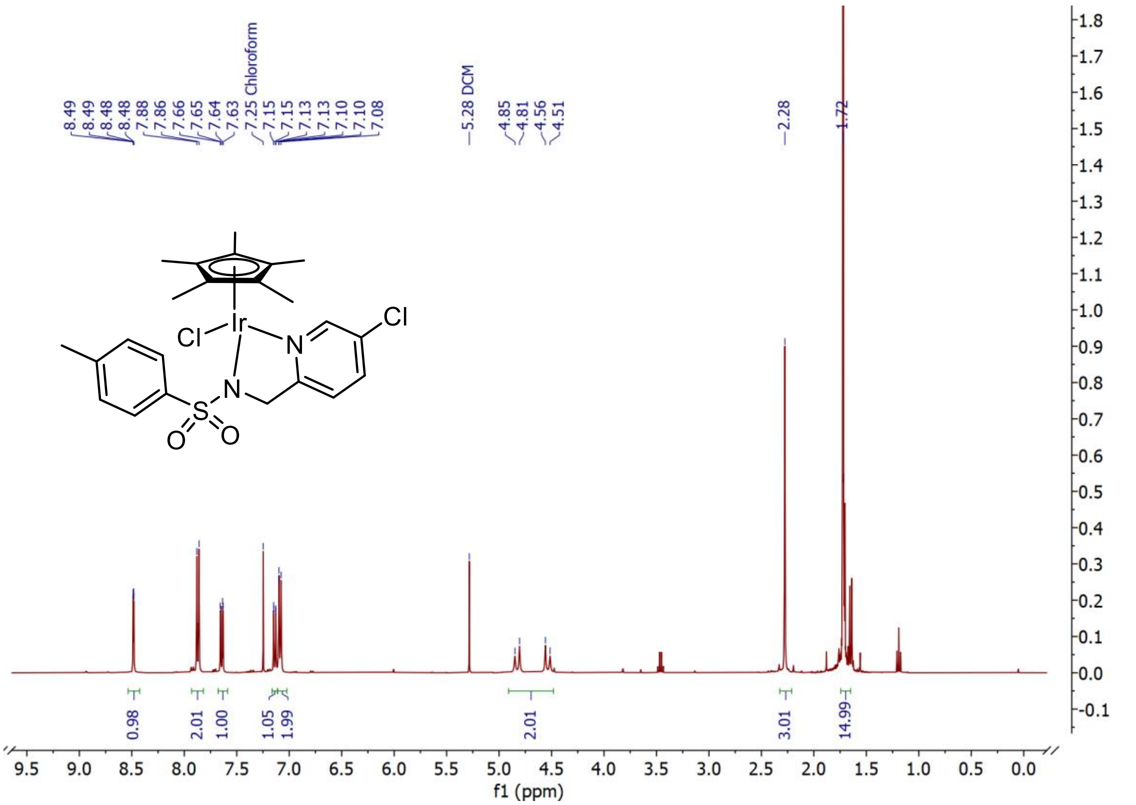

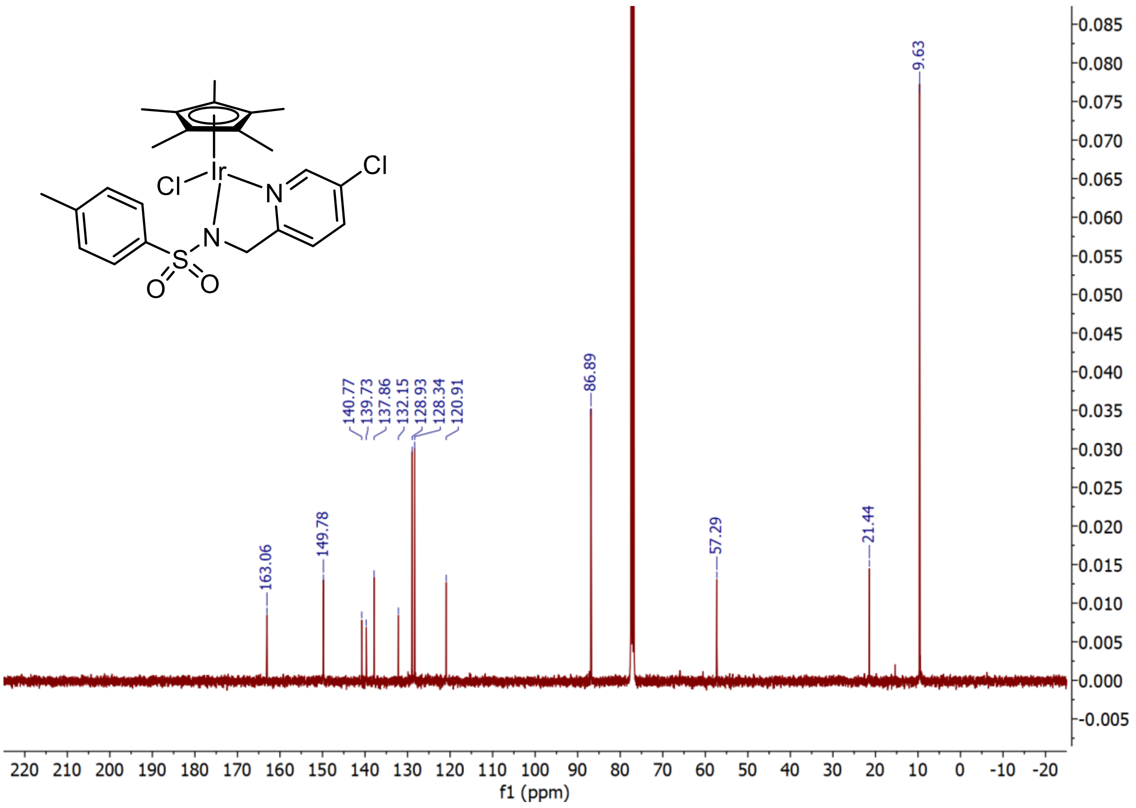

Complex **3c**

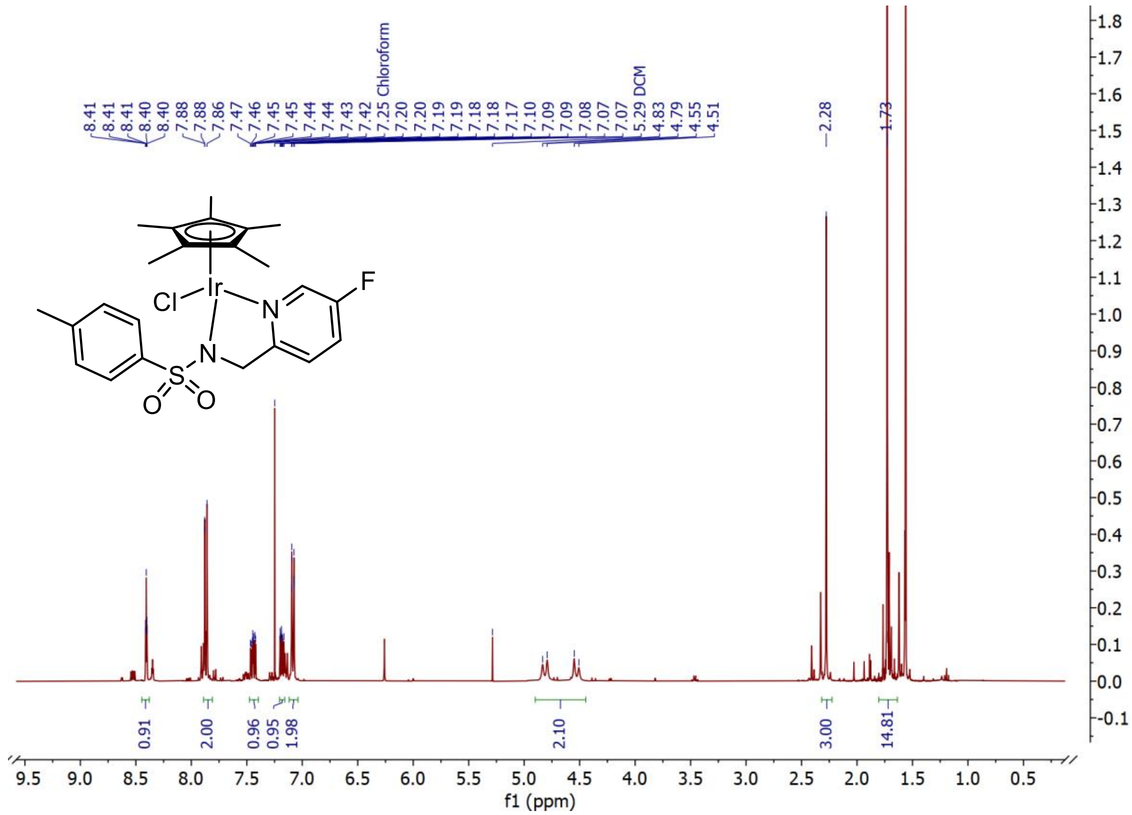

8.41
8.41
8.41
8.40
8.40
7.88
7.88
7.86
7.47
7.46
7.45
7.45
7.44
7.44
7.43
7.42
7.25 Chloroform
7.20
7.20
7.19
7.19
7.18
7.18
7.17
7.10
7.09
7.09
7.08
7.07
7.07
5.29 DCM
4.83
4.79
4.55
4.51
2.28
1.73
Cl
Ir
N
F
N
S
O
O
0.91
2.00
0.96
0.95
1.98
2.10
3.00
14.81
f1 (ppm)

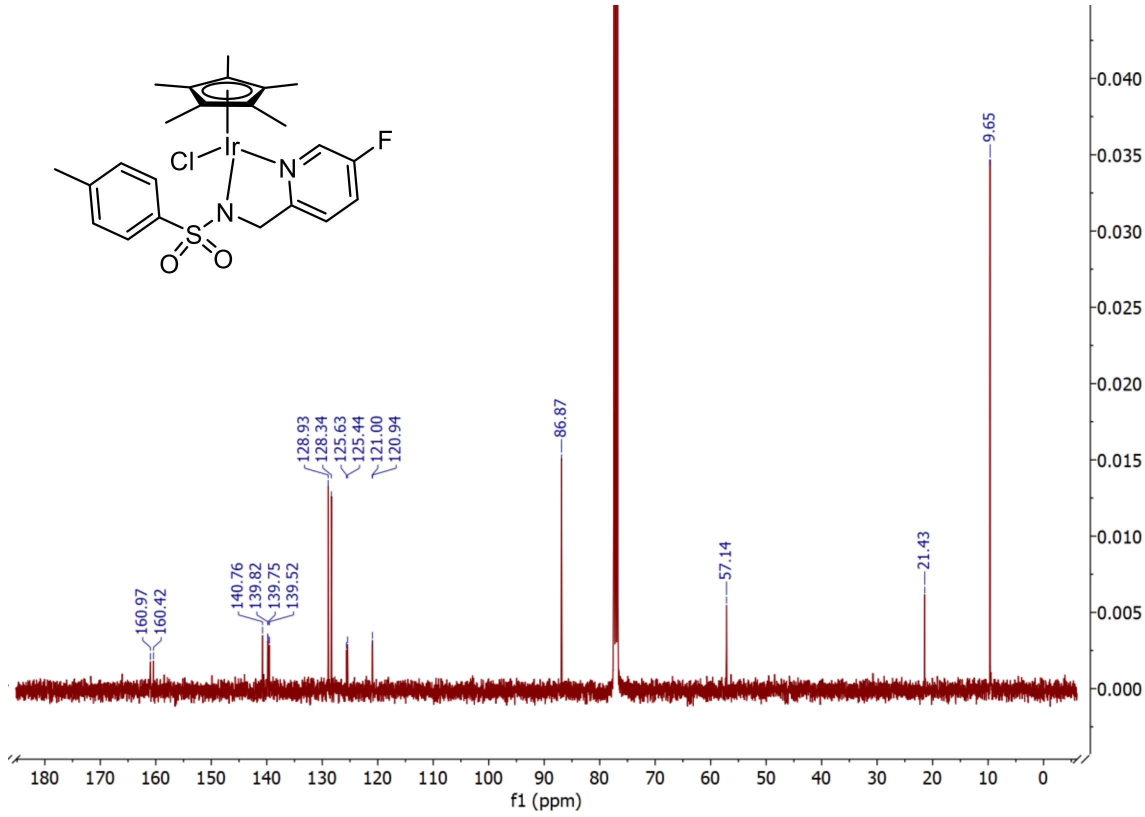

Cl
Ir
N
F
N
S
O
O
160.97
160.42
140.76
139.82
139.75
139.52
128.93
128.34
125.63
125.44
121.00
120.94
86.87
57.14
21.43
9.65
f1 (ppm)

Complex **3d**

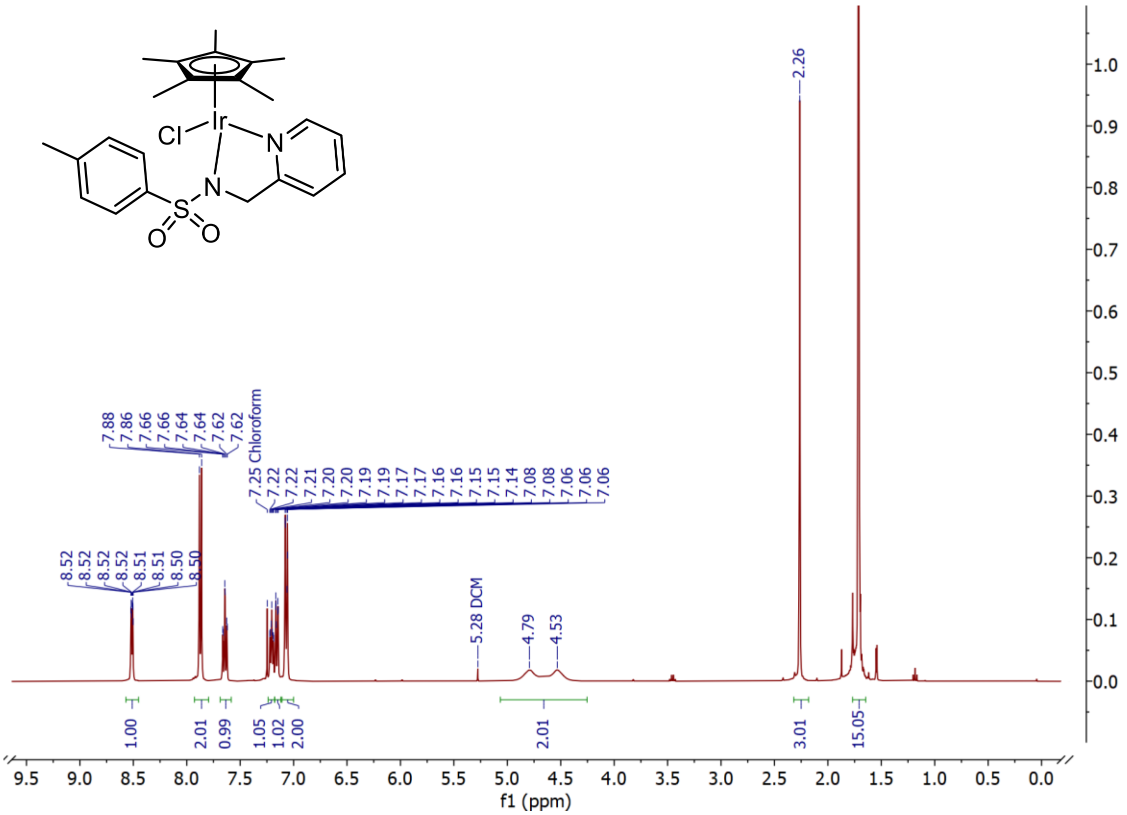
Cl
Ir
N
N
S
O
O
7.88
7.86
7.66
7.66
7.64
7.64
7.62
7.62
7.25 Chloroform
7.22
7.22
7.21
7.20
7.20
7.19
7.19
7.17
7.17
7.16
7.16
7.15
7.15
7.14
7.08
7.08
7.06
7.06
7.06
8.52
8.52
8.52
8.52
8.51
8.51
8.50
8.50
5.28 DCM
4.79
4.53
2.26
1.00
2.01
0.99
1.05
1.02
2.00
2.01
3.01
15.05
f1 (ppm)

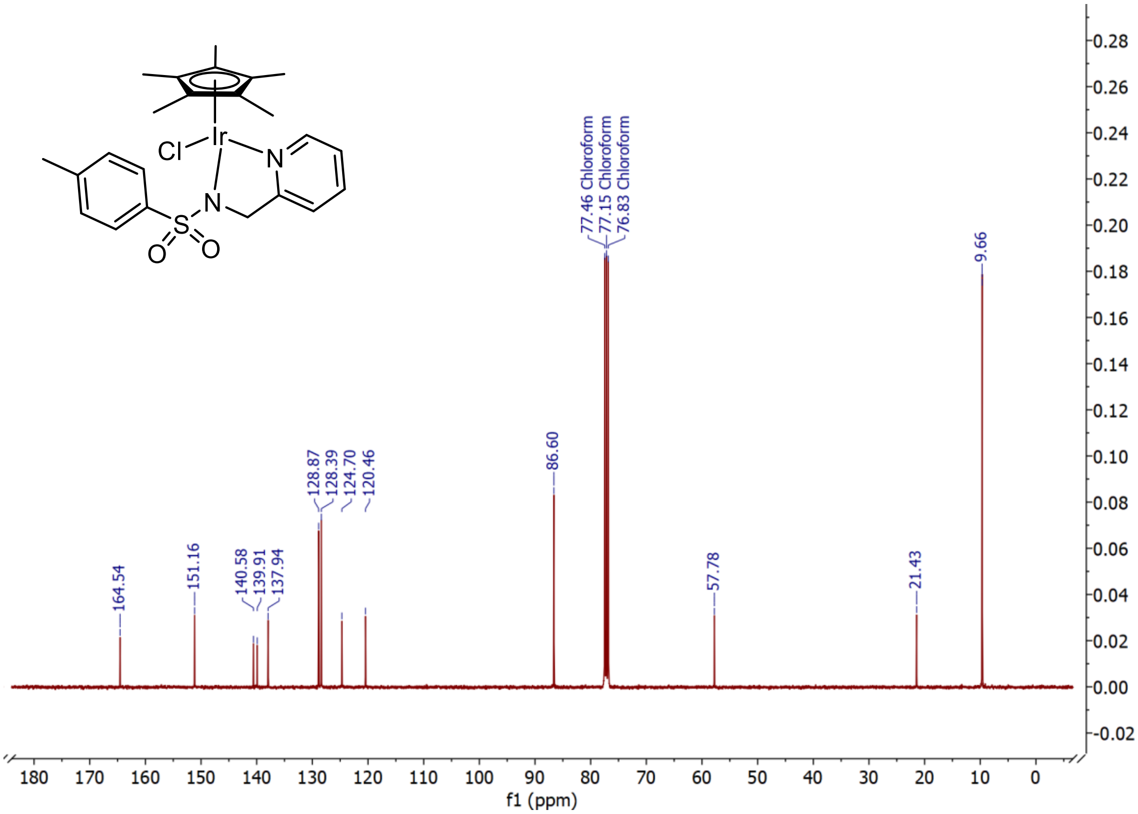
Cl
Ir
N
N
S
O
O
77.46 Chloroform
77.15 Chloroform
76.83 Chloroform
9.66
86.60
128.87
128.39
124.70
120.46
164.54
151.16
140.58
139.91
137.94
57.78
21.43
f1 (ppm)

Complex **3e**

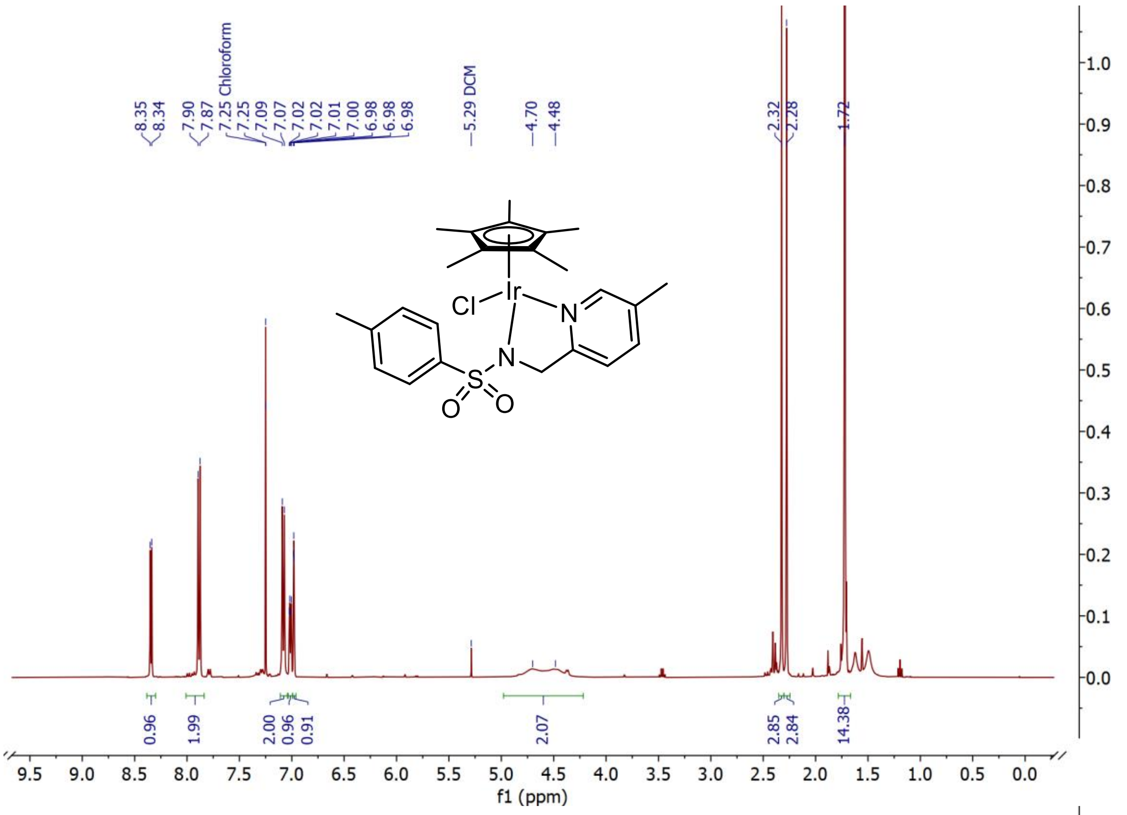
8.35
8.34
7.90
7.87
7.25 Chloroform
7.25
7.09
7.07
7.02
7.02
7.01
7.00
6.98
6.98
6.98
5.29 DCM
4.70
4.48
2.32
2.28
1.72
Cl
Ir
N
N
S
O
O
0.96
1.99
2.00
0.96
0.91
2.07
2.85
2.84
14.38
f1 (ppm)

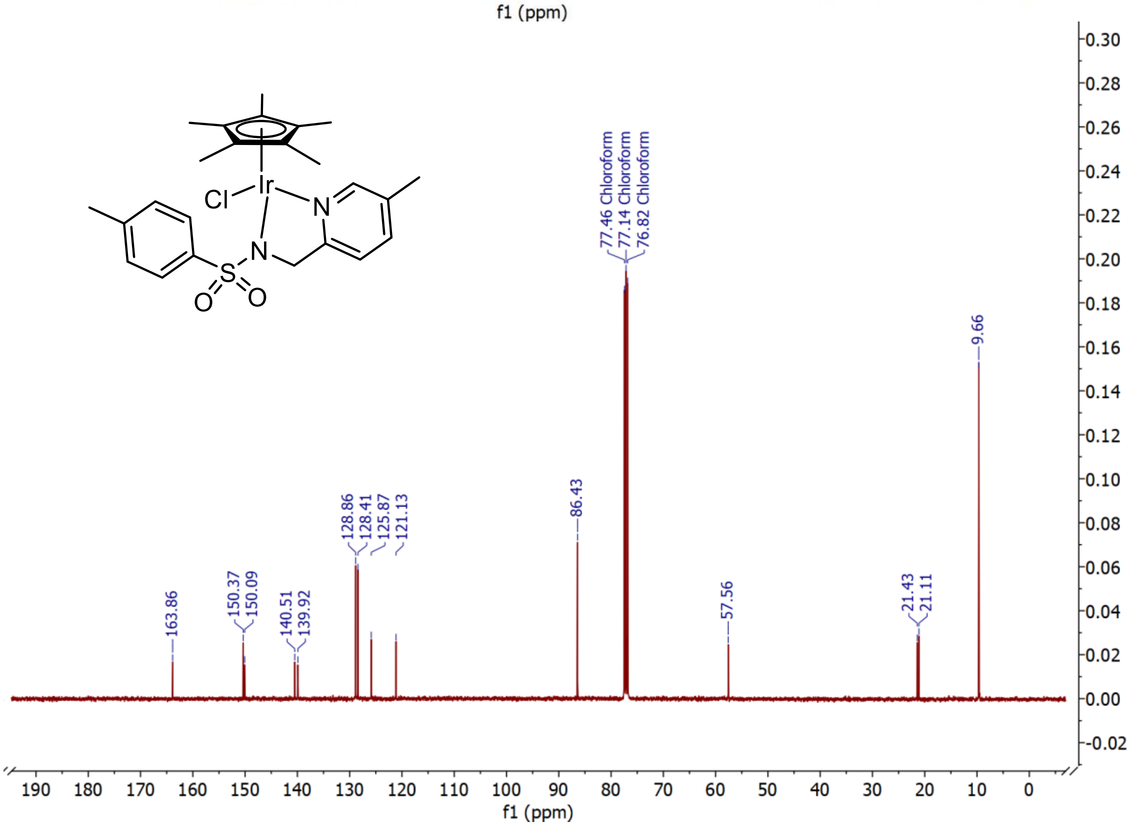
Cl
Ir
N
N
S
O
O
77.46 Chloroform
77.14 Chloroform
76.82 Chloroform
9.66
86.43
128.86
128.41
125.87
121.13
150.37
150.09
140.51
139.92
163.86
57.56
21.43
21.11
f1 (ppm)

Complex **3f**

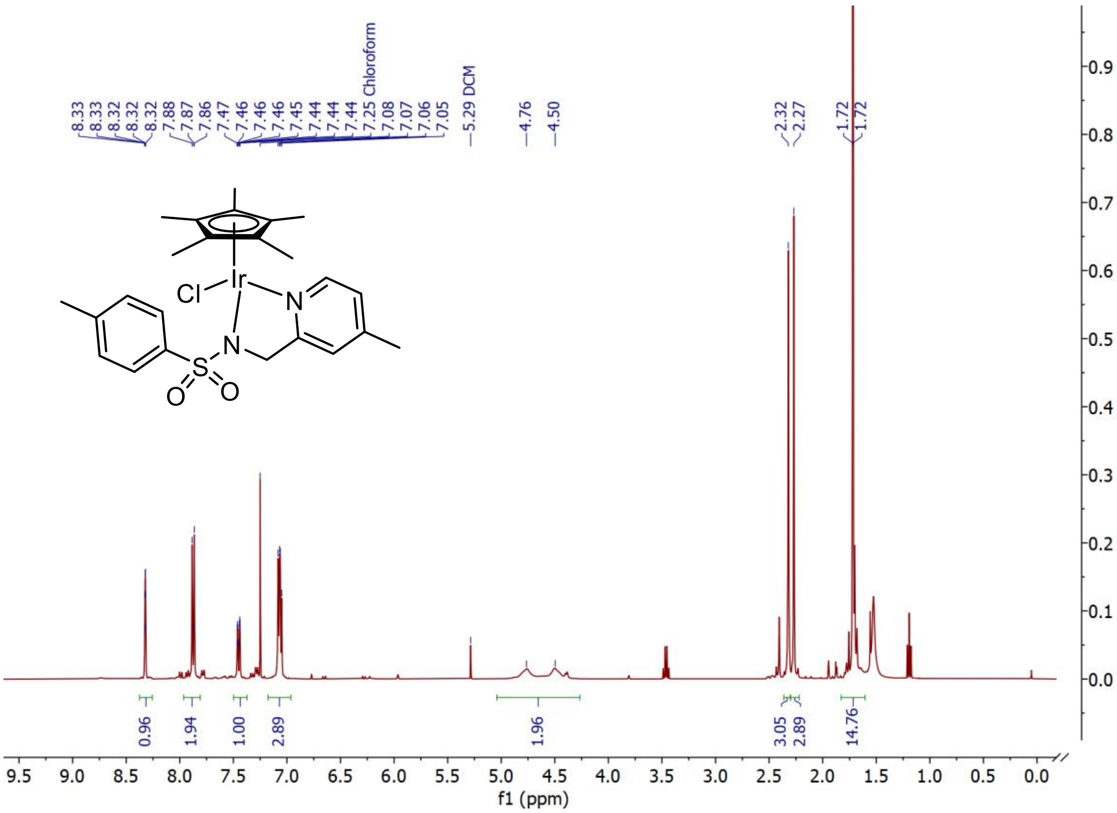

8.33
8.33
8.32
8.32
8.32
7.88
7.87
7.86
7.47
7.46
7.46
7.46
7.45
7.44
7.44
7.44
7.44
7.25 Chloroform
7.08
7.07
7.06
7.05
5.29 DCM
4.76
4.50
2.32
2.27
1.72
1.72
0.96
1.94
1.00
2.89
1.96
3.05
2.89
14.76
f1 (ppm)
Cl
Ir
N
N
S
O
O

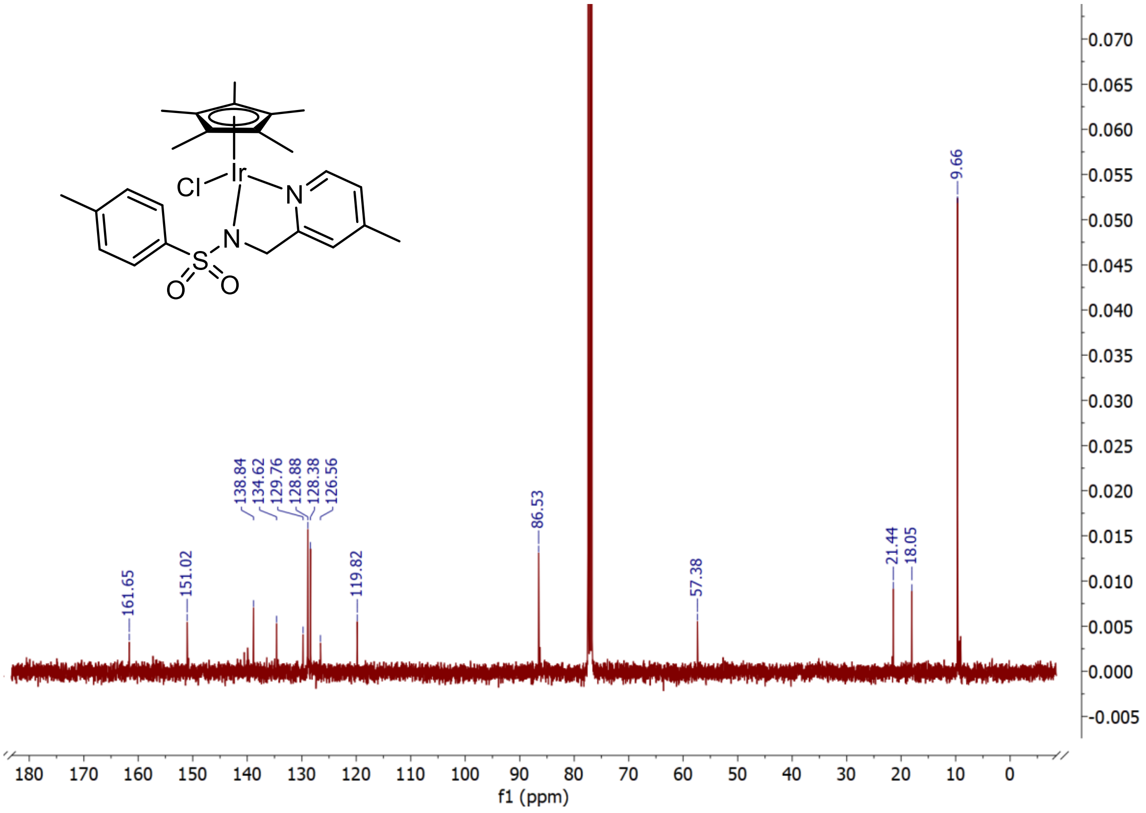

161.65
151.02
138.84
134.62
129.76
128.88
128.38
126.56
119.82
86.53
57.38
21.44
18.05
9.66
f1 (ppm)
Cl
Ir
N
N
S
O
O

Complex **3g**

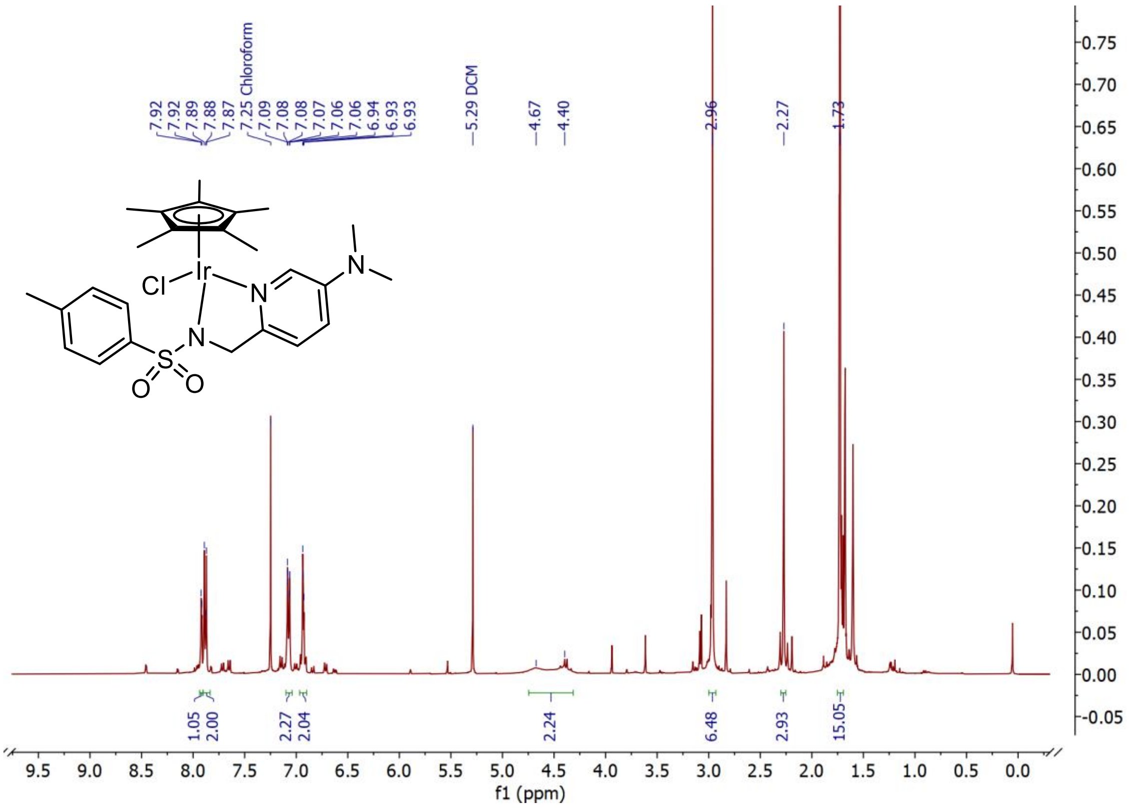

7.92
7.92
7.89
7.88
7.87
7.25 Chloroform
7.09
7.08
7.08
7.07
7.06
7.06
6.94
6.93
6.93
5.29 DCM
4.67
4.40
2.96
2.27
1.73
1.05
2.00
2.27
2.04
2.24
6.48
2.93
15.05
f1 (ppm)
Cl
Ir
N
N
N
S
O
O

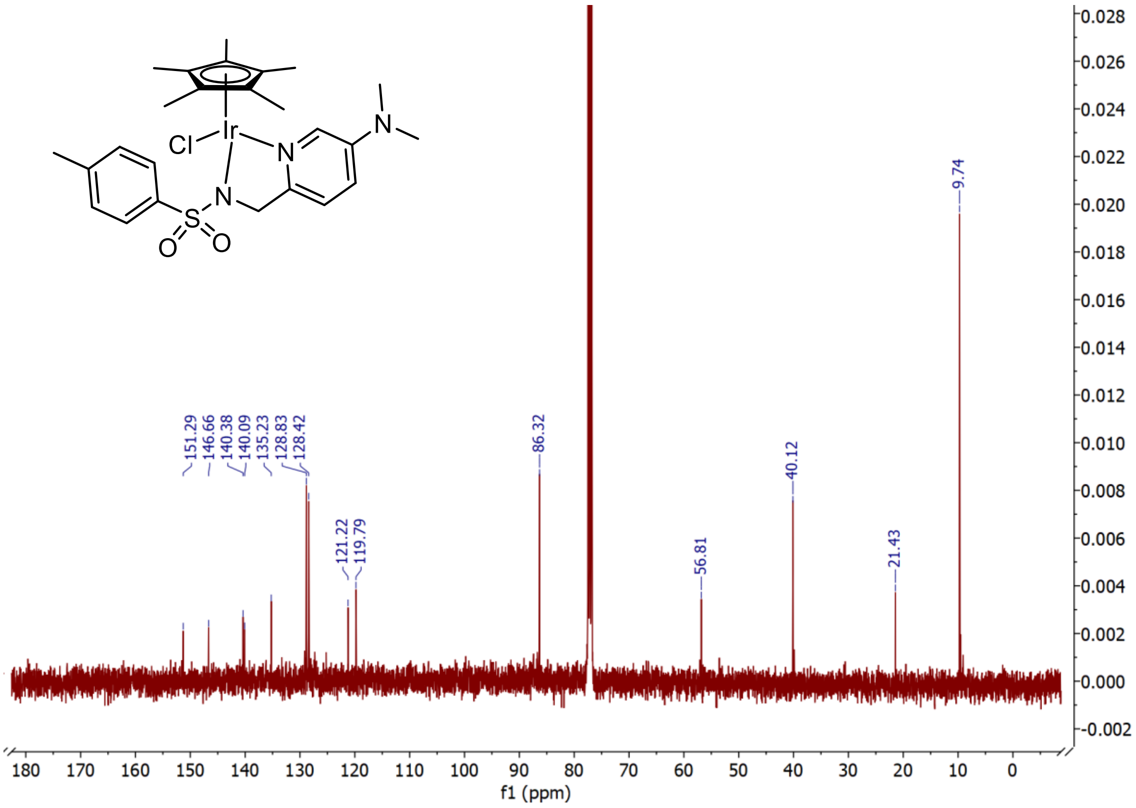

151.29
146.66
140.38
140.09
135.23
128.83
128.42
121.22
119.79
86.32
56.81
40.12
21.43
9.74
f1 (ppm)
Cl
Ir
N
N
N
S
O
O

12. REFERENCES

(1) Raines, D. J.; Clarke, J. E.; Blagova, E. V.; Dodson, E. J.; Wilson, K. S.; Duhme-Klair, A.-K. Redox-Switchable Siderophore Anchor Enables Reversible Artificial Metalloenzyme Assembly. *Nat. Catal.* **2018**, *1* (September), 680–688. https://doi.org/10.1038/s41929-018-0124-3.

(2) Li, M.; Zhang, S.; Zhang, X.; Wang, Y.; Chen, J.; Tao, Y.; Wang, X. Unimolecular Anion-Binding Catalysts for Selective Ring-Opening Polymerization of O-Carboxyanhydrides. *Angew. Chem. Int. Ed.* **2021**, *60*, 6003–6012. https://doi.org/10.1002/anie.202011352.

(3) Ball, R. G.; Graham, W. A. G.; Heinekey, D. M.; Hoyano, J. K.; McMaster, A. D.; Mattson, B. M.; Michel, S. T. Synthesis and Structure of [(η-C5Me5)Ir(CO)]2. *Inorg. Chem.* **1990**, *29*, 2023–2025. https://doi.org/10.1021/ic00335a051.

(4) Gasparro, F. P.; Kolodny, N. H. NMR Determination of the Rotational Barrier in N,N-Dimethylacetamide. *J. Chem. Educ.* **1977**, *54*, 258–261.
